# Beneficial Endophytic Bacteria-*Serendipita indica* Interaction for Crop Enhancement and Resistance to Phytopathogens

**DOI:** 10.3389/fmicb.2019.02888

**Published:** 2019-12-19

**Authors:** Alejandro del Barrio-Duque, Johanna Ley, Abdul Samad, Livio Antonielli, Angela Sessitsch, Stéphane Compant

**Affiliations:** Bioresources Unit, Center for Health and Bioresources, AIT Austrian Institute of Technology, Tulln, Austria

**Keywords:** *Serendipita indica*, bacterial endophytes, symbiosis, *Mycolicibacterium*, tripartite interactions, biocontrol, fungal stimulation

## Abstract

*Serendipita* (=*Piriformospora*) *indica* is a fungal endophytic symbiont with the capabilities to enhance plant growth and confer resistance to different stresses. However, the application of this fungus in the field has led to inconsistent results, perhaps due to antagonism with other microbes. Here, we studied the impact of individual bacterial isolates from the endophytic bacterial community on the *in vitro* growth of *S. indica*. We further analyzed how combinations of bacteria and *S. indica* influence plant growth and protection against the phytopathogens *Fusarium oxysporum* and *Rhizoctonia solani*. Bacterial strains of the genera *Bacillus, Enterobacter* and *Burkholderia* negatively affected *S. indica* growth on plates, whereas *Mycolicibacterium, Rhizobium, Paenibacillus* strains and several other bacteria from different taxa stimulated fungal growth. To further explore the potential of bacteria positively interacting with *S. indica*, four of the most promising strains belonging to the genus *Mycolicibacterium* were selected for further experiments. Some dual inoculations of *S. indica* and *Mycolicibacterium* strains boosted the beneficial effects triggered by *S. indica*, further enhancing the growth of tomato plants, and alleviating the symptoms caused by the phytopathogens *F. oxysporum* and *R. solani*. However, some combinations of *S. indica* and bacteria were less effective than individual inoculations. By analyzing the genomes of the *Mycolicibacterium* strains, we revealed that these bacteria encode several genes predicted to be involved in the stimulation of *S. indica* growth, plant development and tolerance to abiotic and biotic stresses. Particularly, a high number of genes related to vitamin and nitrogen metabolism were detected. Taking into consideration multiple interactions on and inside plants, we showed in this study that some bacterial strains may induce beneficial effects on *S. indica* and could have an outstanding influence on the plant-fungus symbiosis.

## Introduction

In all terrestrial plants and natural ecosystems, multipartite interactions take place between plants and different kinds of microbes (Hardoim et al., 2015). While some bacteria can have a negative impact on the surrounding microflora (Barea et al., 2005; Deveau et al., 2018), other bacteria are known as boosting colonization and establishment of endophytic fungi such as in the case of mycorrhiza (van Overbeek and Saikkonen, 2016), acting therefore as helper bacteria (Frey-Klett et al., 2007). Some of these bacteria are also plant growth-promoting rhizobacteria (PGPR), capable of increasing plant growth and resistance against fungal pathogens by mobilizing nutrients, production of siderophores, auxins, ACC-deaminase, polyamines as well as antibiosis and chitinolytic compounds (Whipps, 2001; Glick, 2014). Most of the helper bacteria were isolated from the rhizosphere. However, some could thrive as endophytes in their host plants, crossing from the root surfaces to the inner plant tissues (Brader et al., 2017). These bacteria are particularly important for the development of biostimulant products, as those microbes with the ability to colonize roots are more prepared to survive and exert beneficial effects on the plant (Compant et al., 2005). Bacteria can further penetrate fungal hyphae and establish symbiosis, playing sometimes critical roles for the survival of the fungal hosts (Bertaux et al., 2005; Bonfante and Desirò, 2017; Guo et al., 2017). Fungal hyphae provide a nutrient-rich niche for bacterial growth (Linderman, 1992; Boer et al., 2005; Scherlach et al., 2013). The hyphae-associated bacteria may reciprocally promote hyphal development and root colonization through the supply of vitamins, nitrogen, phosphorus, sugars, and secondary metabolites to the fungal partner or by increasing ATP production (Hildebrandt et al., 2006; Ghignone et al., 2012), as exemplified by the mycorrhizal helper bacteria of arbuscular mycorrhizal fungi (AMF) (Frey-Klett et al., 2007). However, bacteria with suppressive effects on fungi (e.g. secretion of antifungal compounds, mycophagy) have also been widely reported (Bonfante and Anca, 2009; Kobayashi and Crouch, 2009). Understanding and exploiting the association between different kinds of beneficial microorganisms, together with the management and engineering of the plant microbiome, may lead to improving soil fertility, crop productivity, and biological control of plant pathogens (Berg, 2009; Collinge et al., 2019; Compant et al., 2019).

An example of beneficial fungi interacting with symbiotic bacteria is *Serendipita indica*, previously known as *Piriformospora indica* (Varma et al., 1999). It is a well-known root endophytic fungus that mimics the capabilities of AMF, but in contrast to AMF it can be cultured axenically (Varma et al., 1999). The fungus improves crop yield and confers resistance against biotic and abiotic stresses by triggering induced systemic resistance (ISR), boosting antioxidant capacity, mobilizing nutrients, and manipulating the hormone levels of the plant (Franken, 2012; Gill et al., 2016). Interestingly, it has been demonstrated that this fungus hosts an endobacterium, *Rhizobium radiobacter* (Sharma et al., 2008), which has endophytic as well as plant growth-promoting properties, although its functional role is not yet fully understood (Glaeser et al., 2016). Only few studies have demonstrated *in vitro* that particular bacterial strains can be detrimental for the growth of *S. indica* (Varma et al., 2013) or in contrast have stimulatory effects, like strain WR5 of *Azotobacter chrococcum*, which enhanced mycelial growth and sporulation of *S. indica in vitro* (Bhuyan et al., 2015). In the last few years, some researchers have developed co-inoculations (microbial consortia) of fungi and bacteria, searching synergisms between two beneficial microbes (Artursson et al., 2006; Collinge et al., 2019). Special focus has been laid on the combination of *S. indica* with PGPR. Kumar et al. (2012) detected increased plant growth by combining *S. indica* and pseudomonad R81. However, combinations of *S. indica* and bacteria have not always been successful (Sarma et al., 2011). Similarly, several combinations of different biocontrol agents have not improved the effect exerted by the most efficacious one, indicating no synergistic but more likely antagonistic interactions (Xu et al., 2011). This suggests that more research is needed in the exploration of compatible combinations. We hypothesized, nevertheless, that several bacterial taxa or strains can positively interact with the beneficial fungus *S. indica* and that these bacterial-fungal interactions could be exploited for crop enhancement and resistance against different phytopathogens.

The aim of this study was to identify bacterial taxa that stimulate the growth and effects of the beneficial fungus *S. indica* and to select the most promising “fungus-conducive” bacteria for dual-inoculation with *S. indica*. The effect of these combinations on plant growth and biocontrol activity against fungal pathogens was further assessed. In particular, we aimed at researching the tripartite interactions between *S. indica*, various bacterial strains and the plant pathogens *F. oxysporum* or *R. solani*. We further explored potential mechanisms involved using genome analysis of most promising bacterial endophytes showing positive interaction with *S. indica*.

## Materials and Methods

### *Serendipita indica* Cultivation

*Serendipita* (=*Piriformospora*) *indica* strain DSM 11,827 was provided by Pr. Philipp Franken and obtained from the “Deutsche Sammlung für Mikroorganismen und Zellkulturen,” Braunschweig, Germany (Varma et al., 1999). The fungus was maintained at −80°C in sterile Potato Dextrose Broth (PDB) (Carl Roth, Germany) amended with 25% glycerol and grown on Potato Dextrose Agar (PDA) plates or in liquid culture containing *Aspergillus* complete medium (Pontecorvo et al., 1953).

To produce inoculum, roughly fifty 2-mm agar plugs from a 2-week old culture of *S. indica* grown on PDA were transferred to 250 ml Erlenmeyer flasks containing 100 ml of *Aspergillus* CM and incubated for 3 weeks under constant shaking (150 rpm) at 26 ± 1°C. Mycelium and spores were collected by centrifugation (4,500 rpm, 5 min) and the remaining pellet was washed 3–5 times with sterile phosphate-buffered saline (PBS) of pH 6.5. The mixture of mycelium and spores, resuspended in PBS, was ground with a homogenizer Ultra Turrax T25 (IKA^®^, Staufen, Germany) for 3 min in intervals of 30 s. The number of spores + mycelium fragments was estimated with a hemocytometer (NanoEnTek, Seoul, Korea) and the viability of the CFU confirmed by plating on PDA. Final concentrations were adjusted with PBS.

### Plant Assay for Isolation of Endophytic Bacteria

An agricultural soil was sampled in a field located in Meires, Lower Austria, Austria (48°46′43.8″N; 15°17′23.4″E) at a depth of 5–15 cm and stored at 4°C. To create an isolated microsystem, one potato tuber (*Solanum tuberosum* L. cv. Romina; NÖ. Saatbaugenossenschaft, Austria) and four tomato seeds (*Solanum lycopersicum* L. cv. Moneymaker; Austrosaat, Vienna, Austria) were grown in closed Magenta boxes (Sigma-Aldrich) containing ~200 g of the agricultural soil. The plants were kept in greenhouse with a Day/Night temperature of 22/21°C, a relative humidity 50/35% and a 12 h light/dark photoperiod. Plants were watered weekly with 20 ml of water. After 4 weeks, potato and tomato plants were inoculated by drenching a mixture of spores + fragmented mycelium of *S. indica* at 10^4^ CFU/g of soil. Control treatment was mock-inoculated with PBS. To mitigate a possible big shift in the bacterial community caused by *S. indica*, an additional treatment with low-concentrated (10^2^ CFU/g). *S. indica* inoculum was included for potato plants. In total five treatments were prepared with three replicates (boxes) per treatment (Supplementary Figure 1).

### Isolation of Endophytic Bacteria

Three weeks after inoculation, 1.5 g of potato and 0.2 g of tomato roots were harvested from each magenta box to isolate endophytic bacteria. For this, roots were rinsed abundantly with tap water, then surface-sterilized with 70% ethanol for 10 s followed by 2.5% sodium hypochlorite for 3 min and then rinsed 3 times with sterile water. For each sample, 100 μl of the final wash were plated in triplicates on Nutrient Agar No2 (NA) (Sigma-Aldrich, St. Louis, USA) until 6 days of incubation at 26°C to confirm the surface sterilization. To isolate bacteria, sterile roots, macerated in 5 ml of sterile 0.85% NaCl, were smashed with a mortar and pestle and homogenized by vortexing for 30 s at maximum speed. Smashed roots from each sample were 10-fold diluted on PBS and 100 μl from each dilution were plated on NA and further incubated at 26°C for 6 days. Based on visual differences in morphology, single colonies were randomly picked only from the most diluted cultures to avoid contaminations. Selected bacteria were further purified by repeating streaking on NA plates and all the recovered isolates were stored at −80°C in Nutrient Broth (NB) (Difco, Detroit, MI) supplemented with 25% glycerol. At least 100 isolates were obtained from every treatment with potato plants, and 50 with tomato plants.

### DNA Isolation and 16S rRNA Gene Sequencing

Bacterial DNA of each isolate was extracted using the UltraClean^®^ Microbial DNA Isolation Kit (QIAGEN, Venlo, Netherlands) according to the manufacturer's instructions. The 16S rRNA genes were amplified using the primers 8F (5′-AGAGTTTGATCCTGGCTCAG-3′) (Weisburg et al., 1991) and 1520R (5′-AAGGAGGTGATCCAGCCGCA-3′) (Edwards et al., 1989). A conventional PCR amplification of 20 μl PCR reaction mix containing 2.5 mM MgCl_2_, 0.2 mM dNTPs, 0.3 mM of each primer, 1–2 μl of DNA template, 1 U HOT FIREPol^®^ DNA polymerase (Solis BioDyne) and 1 × PCR reaction buffer (Invitrogen) was carried out in a thermocycler peqSTAR 96X HPL (PEQLAB Biotechnologie GmbH). An initial denaturation step at 95°C for 5 min was followed by 30 cycles of denaturation at 95°C for 45 s, annealing at 54°C for 60 s and elongation at 72°C for 90 s, plus a final extension at 72°C for 10 min were performed. Sequencing of the PCR product was performed by LGC-Genomics (Teddington, UK) using the primers 8F and 1495r (5′-CTACGGCTACCTTGTTACGA-3′) (Lane, 1991). To remove duplicate sequences from the library, sequences were de-replicated and clustered at 100% similarity with the Avalanche NextGen Workbench (http://www.visualbioinformatics.com/bioinf/index.html). Strains showing the same partial 16S rRNA genes and the same phenotypic interaction with *S. indica* were removed. The identification of isolates was performed by BLAST search on a local installation of the complete NCBI's nt database (downloaded in October 2018), targeting the first more significant 50 hits. Taxonomic assignment of BLAST hits was then refined with the approach implemented in BlobTools (Laetsch and Blaxter, 2017). Sequence data are available at NCBI database and GenBank under the accession numbers MN180888–MN181366.

### Effect of Endophytic Bacteria on Mycelial Growth of *S. indica*

To determine the effect of bacteria on *S. indica*, the growth of the fungus in interaction with each endophytic bacterium was expressed in terms of hyphae expansion on agar plates. For this, bacteria were pre-cultured for 4 days on NA and the fungus for 2 weeks on PDA. To study the interaction between bacteria and *S. indica*, each bacterium was then streaked on 1 cm^2^ in the center of a Petri dish (9 cm Ø, containing 15 ml PDA) and a 0.5-cm^2^ agar plug of active *S. indica* mycelium was placed inverted over the streaked bacterium. As a control, *S. indica* was grown alone. All the co-cultures were replicated four times. Aiming a confirmation of results, some selected isolates were additionally co-cultured with *S. indica* under different nutrient conditions (a mixture 1:1 of PDA+NA), and under longer bacterial growth phase (9 days of bacterial preculture).

**Figure 1:**
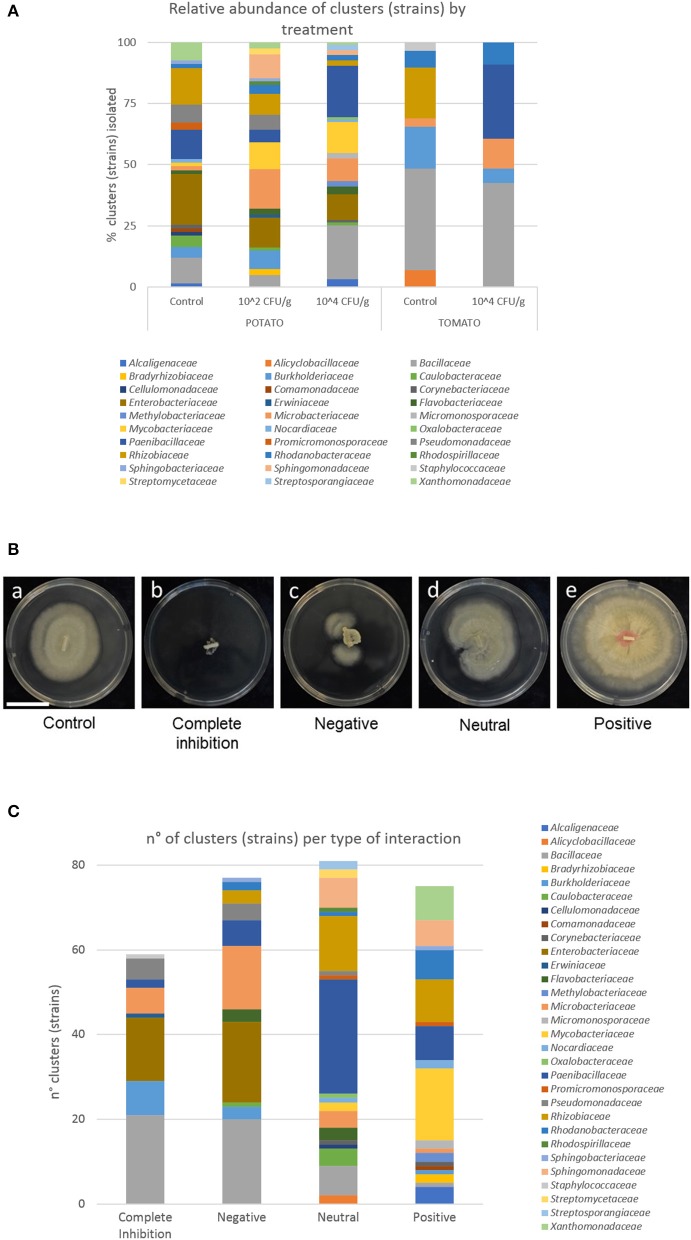
Isolates of bacteria and interaction with *S. indica*. **(A)** Relative abundance of bacterial families isolated from potato and tomato roots, and from plants pre-inoculated or not with *S. indica*. **(B)** Growth of the fungus alone (a) and in combination with bacteria completely inhibiting fungal growth (b), interacting negatively (c), in a neutral way (d), and stimulating the growth of the fungus (e). Bar corresponds to 3 cm. **(C)** Number of bacterial clusters (strains) and their phylogeny per type of interaction.

Plates were incubated at 26°C in darkness. After 13 days of dual-culturing, the surface of the plate covered with *S. indica* mycelium was measured with ImageJ 1.48 software (https://imagej.nih.gov/ij/) and the average measurement of the four replicated plates was employed to determine the type of interaction. The difference in growth between *S. indica* co-cultured with a bacterium (dual-cultured *S. indica*) and control was calculated as relative increase/decrease of dual-cultured *S. indica* growth respect to the control. To characterize the type of interaction between fungus and bacteria, an arbitrary scale was further established. When the growth of dual-cultured *S. indica* was reduced by more than 90% in respect to the control, the interaction was considered as a complete inhibition. A reduction between 90 and 20% was determined as a negative interaction. If the growth of dual-cultured *S. indica* was decreased or increased up to 20% respect to the control, a neutral interaction was established. An increment of dual-cultured *S. indica* growth larger than 20% defined a positive interaction (Figure 1B).

### Combining Selected Beneficial Endophytic Bacteria and *S. indica* for Tomato Growth Promotion

Since most of the isolates from one genus (*Mycolicibacterium*) stimulated *S. indica* growth, four isolates (P1-5, P1-18, P9-22, and P9-64) of this genus were selected for further experiments. With the aim of studying the effect of dual inoculations of *S. indica* and bacteria on tomato plants, a pot experiment was conducted in the greenhouse.

For the pot experiment, *S. indica* inoculum was produced as described above. In the case of bacteria, they were grown for 2.5 days in 10 ml bottom-rounded Falcon tubes containing 5 ml NB at 26°C and constant shaking (190 rpm). The cultures were centrifuged (4,600 rpm, 6 min, room T°C) and washed 3 times with sterile PBS to remove traces of media. Cell growth was determined by measuring the OD and CFU were estimated by standard serial dilution on NA.

Tomato seeds cv. Moneymaker were germinated for 4 days in a Whatman^®^ filter paper (110 mm Ø) at room temperature on Petri dishes. Germinated seeds were transferred to 50 ml falcon tubes containing 15 ml of PBS and either (i) bacterial cells (5 × 10^7^ CFU/ml), (ii) spores + hyphae fragments of *S. indica* (5 × 10^5^ CFU/ml), or (iii) a mixture of bacterial cells (5 × 10^7^ CFU/ml), and *S. indica* (5 × 10^5^ CFU/ml). Falcon tubes were maintained in a Tube Roller RS-TR5 (Phoenix Instrument GmbH, Garbsen, Germany) for 30 min. For control treatment, the seeds were immersed in 1 × PBS.

Two seeds per pot were then sown at 1 cm depth in pots (1 l capacity) containing the substrate “Fruhstorfer Erde Typ Nullerde” (Hawita Gruppe, Vechta, Germany). The experiment had 10 treatments, ± *S. indica* and ± individual bacterial strains with 14 replicates (7 pots × 2 plants per pot) for each treatment. The plants were grown in the greenhouse (with conditions described before) and watered twice a week with tap water. Plants were harvested 6 weeks after planting and shoot fresh weight and leaf area were measured (using ImageJ). A confirmatory experiment was repeated with a richer soil in which pots were filled with a mixture (1:1:1 v/v) of perlite, sand, and the substrate “Tonsubstrat ED63 Special” (Einheitserde, Germany). After harvesting, shoot fresh and dry weight (after oven-drying for 3 days at 70°C) were measured.

### *In vitro* Interaction Between Selected Bacteria and Plant Pathogens

In addition to determining the effect of selected bacteria on the growth of *S. indica* and tomato plants, they were also tested for their effects on pathogens such as *Fusarium oxysporum* f. sp. *lycopersici* Fol4287 (kindly provided by Maria E. Constantin, University of Amsterdam, Netherlands) (Di Pietro and Roncero, 1996) and *Rhizoctonia solani* AG-3 (kindly provided by Rosanna C. Hennessy, University of Copenhagen, Denmark). These fungi were maintained at −80°C in PDB amended with 25% glycerol. *In vitro* dual culture assays between these fungal pathogens and selected bacterial isolates were performed as described above for the beneficial fungus *S. indica*, but due to different growth rates, fungal preculture and final measurements were shortened to 3 days in case of *R. solani*, and 6 days for *F. oxysporum*.

### Tomato Protection Against *Fusarium oxysporum* and *Rhizoctonia solani* Using Multipartite Interaction

Biocontrol of *F. oxysporum* was evaluated by pot experiment in which plants were infected with the pathogen and with single or dual inoculations of *S. indica* and selected bacteria. Inoculum production of *S. indica* and bacteria and seed inoculation were carried out as described above. The seeds were planted in 1 l pots containing a mixture (1:1:1 v/v) of perlite, sand and the substrate “Tonsubstrat ED63 Special” (Einheitserde, Germany) (2 seeds per pot, 5 pots per treatment).

For production of *Fusarium*, spores were obtained according to van der Does et al. (2019). Briefly, an agar plug from a 6-day old PDA culture of *Fusarium* was transferred to a 250-ml Erlenmayer flask containing 100 ml minimal media (3% sucrose, 0.17% yeast nitrogen base without amino acids or ammonia, and 100 mM KNO_3_), and incubated for 5 days at 26°C, 190 rpm. Spores were filtered through a Miracloth filter (Millipore), washed twice with sterile 1 × PBS and diluted to a concentration of 10^7^ spores/ml.

Ten days after planting, tomato plants were infected with the solution of *F. oxysporum* described before according to the root dip method (Wellman, 1939). Seedlings were uprooted and trimmed leaving roughly 1 cm of root, to facilitate the penetration of *Fusarium*. Roots were placed for 30 min in the spore suspension of *Fusarium*, and directly repotted. Five weeks after inoculation, plant weight above the cotyledons was measured, and the extent of disease progression was scored according to de Lamo et al. (2018). Briefly, disease index was 0 = no symptoms, 1 = one brown vessel above the soil, 2 = one or two brown vascular bundles at the cotyledon level, 3 = at least three brown vessels and growth distortion, 4 = all vessels brown or the plant is small and wilted, 5 = dead plant.

The effect of dual-inoculation of *S. indica* and endophytic bacteria against the damping-off causative agent *R. solani* was analyzed, in parallel to *F. oxysporum* test, by a germination assay in closed boxes (Steri Vent Containers 107 × 94 × 96 mm, Duchefa Biochemie b.v, Haarlem, Netherlands). These boxes contained 120 g of a sterile (2 times, 121°C, 20 min) 1:4 mixture (w/w) of vermiculite (2–3 mm, Sigma-Aldrich) and distilled water. Tomato seeds cv. Moneymaker were surface-sterilized with 2.5% sodium hypochlorite for 5 min and rinsed 8 times with sterile water. Seeds were inoculated with either (i) *S. indica*, (ii) (x4) bacteria, or (iii) (x4) combination of fungus and bacteria as earlier described.

For this, *Rhizoctonia* was cultured on 1/5 PDA for 3 weeks. Five agar plugs of *Rhizoctonia* mycelium were placed in a row in the center of each box, and 2 rows of tomato seeds (5 seeds per row) were sown on both sides of the pathogen row at 2 cm distance. Both phytopathogen and seeds remained at 0.5 cm depth. Control was prepared with plugs of 1/5 PDA. Three replicated boxes were prepared per treatment and maintained in the greenhouse (see above). The number of germinated seedlings per box was monitored regularly for 4 months by scoring as follows: 1 = Plant germinated and no disease symptoms, 0.5 = Plant germinated, alive, with necrotic areas in leaves and stems, 0 = plant dead.

### Bacterial Genome Sequencing and Analysis

Bacterial genomic DNA from 4 selected *Mycolicibacterium* strains were isolated using a phenol-chloroform based protocol according to Samad et al. (2016). Concisely, cells were grown on NB for 3 days and collected by centrifugation. Each bacterial pellet was resuspended in lysis buffer (5 mM EDTA pH8, 50 mM Tris-Cl, 1% SDS, 0.5 M NaCl, 0.2 mg/ml Proteinase K) and incubated at 65°C overnight, 400 rpm. DNA was extracted 2 times using 1 volume of phenol-chloroform-isoamylalcohol (25:24:1) and collected by centrifugation. Genomic DNA was further cleaned with Amicon Ultra 0.5 mL 30K Centrifugal Filter Units (Millipore, Cork, Ireland) and re-suspended in water. Whole-genome shotgun sequencing was performed on an Illumina HiSeq (GATC Biotech, Konstanz, Germany), producing 2 × 150 bp reads.

Illumina reads were checked for the presence of PhiX using Bowtie 2 (v2.3.4.3) (Langmead and Salzberg, 2012) and adapters were removed with fastp (v0.19.5) (Chen et al., 2018). Sequence quality and length distribution were checked via FastQC^1^ (Andrews, 2010). Genome assembly was carried out with SPAdes v3.13.0 (Bankevich et al., 2012) and short (<500 bp), low-abundant (<2×) contigs filtered out. The presence of contaminant contigs was assessed using BlobTools and alien contigs were eventually removed. Genome assembly quality was then inferred using QualiMap v2.2 (Okonechnikov et al., 2015) and QUAST v5.0.0 (Gurevich et al., 2013) and genome completeness reconstruction was evaluated with BUSCO v3.0 (Waterhouse et al., 2017). Gene annotation was performed using Prokka v1.12 (Seemann, 2014) and NCBI Prokaryotic Genome Annotation Pipeline (PGAP). Contigs were further screened for the presence of antimicrobial resistance or virulence genes with ABRicate v0.8.10. The presence of plasmids was ascertained by using Mash v2.1 against the PLSDB database (Galata et al., 2018).

Functional annotation was performed using EggNOG 4.5 (Huerta-Cepas et al., 2015) and the ClassicRAST (Rapid Annotation using Subsystem Technology) web server (http://rast.nmpdr.org) (Aziz et al., 2008). Prediction of biosynthetic gene clusters and secondary metabolites was additionally carried out using antiSMASH version 4.0.2 (Weber et al., 2015). CAZy families were identified with dbCAN2 according to the DIAMOND database. A cutoff of E-Value of 1e-102 was set for the output. When a gene contained a CBM with other CAZy classes, the gene was classified as CBM. Protein annotation was based on the CAZy database (Cantarel et al., 2008; Lombard et al., 2013).

The Average Nucleotide Identity (ANI) analysis was further used to determine the relatedness between the assembled genomes and affiliated genomes available in NCBI database classified as *Mycobacterium* or *Mycolicibacterium*. For this, 169 genomes were downloaded using the script available at https://github.com/kblin/ncbi-genome-download and ANI was calculated with the pyani Python module available at https://github.com/widdowquinn/pyani, using BLAST (ANIb) and TETRA methods. Based on the ANI pair-wise values, a distance matrix representing ANI-divergence (defined as 100% ANI data) (Chan et al., 2012) was compiled to display a heat map and compute a dendrogram using the hierarchical clustering adopting the complete linkage algorithm, with the software Morpheus (https://software.broadinstitute.org/morpheus/).

The draft genome sequences for the *Mycolicibacterium* strains P1-5, P1-18, P9-22, and P9-64 are available at NCBI, BioProject PRJNA393298, with the DDBJ/ENA/GenBank accession numbers NPKT00000000, NPKR00000000, NPKP00000000, and NPKO00000000, respectively.

### Statistical Analysis

Statistical analysis of *in vitro* fungal growth, analysis of biomass and leaf area of samples from the growth enhancement experiments were performed in R 3.5.1 (R Core Team, 2019). Data distributions were checked using the fitdistrplus package (Delignette-Muller and Dutang, 2015) and linear or linear mixed-effects models (nlme R package) (Pinheiro et al., 2019), when applicable, were generated. After graphical verification of homogeneity assumption, ANOVA was applied on previously generated models, followed by pairwise comparisons (Tukey's method, *P* = 0.05) calculated using Estimated Marginal Means (emmeans R package) (Lenth, 2019). Quantitative data were processed with dplyr package (Wickham et al., 2019) and results visualized with boxplots using ggplot2 package (Wickham and Chang, 2016). The samples from the biocontrol experiment were analyzed with PRISM 8.0 (GraphPad). Concerning *Fusarium*, a non-parametric Mann-Whitney *U*-test was applied on the fresh weight and disease index data (de Lamo et al., 2018). The germination assay with *Rhizoctonia* was analyzed by one-way ANOVA and Tukey's test (*P* = 0.05).

## Results

### Isolation and Identification of Bacteria

In total, 479 isolates were recovered and identified at the genus level (Table 1). The most abundant families of isolates were *Bacillaceae* (21.09% of the total isolates), *Enterobacteriaceae* (13.78%), *Rhizobiaceae* (11.48%), and *Paenibacillaceae* (10.02%) (Table 1 and Supplementary Figure 2A). At the genus level, the overall top five genera were *Bacillus* (21.09% of isolates), *Enterobacter* (13.57%), *Paenibacillus* (8.56%), *Burkholderia* (7.52%), and *Agrobacterium* (6.05%). After de-replication with a 100% threshold, we obtained 260 different clusters (strains) with different 16S rRNA genes. The most abundant strains belonged to the families *Bacillaceae* (17.31%), *Paenibacillaceae* (15.0%), *Enterobacteriaceae* (11.15%), and *Microbacteriaceae* (9.23%). At the genus level, the most abundant strains belonged to *Bacillus* (17.31%), *Paenibacillus* (12.69%), *Enterobacter* (10.77%), and *Mycolicibacterium* (7.31%).

**Table 1:** Species identified by sequencing the 16S rRNA gene and number of isolates and clusters (strains) assigned to species.

**Order**	**Family**	**Genus**	**Species**	**Clusters**	**Isolates**	**Potato**			**Tomato**	
						**Ctrl**.	**10^**2**^ CFU**	**10^**4**^ CFU**	**Ctrl**.	**10^**4**^ CFU**
Caulobacteriales	*Caulobacteraceae*	*Brevundimonas*	*B. lenta*	1	2	1	0	1	0	0
			*B. vesicularis*	1	1	0	1	0	0	0
		*Caulobacter*	*C*. sp.	2	2	2	0	0	0	0
Rhizobiales	*Bradyrhizobiaceae*	*Bosea*	*B. thiooxidans*	1	1	0	1	0	0	0
		*Tardiphaga*	*T. robiniae*	1	1	0	1	0	0	0
	*Methylobacteriaceae*	*Methylobacterium*	*M. tardum*	2	2	0	0	2	0	0
	*Rhizobiaceae*	*Agrobacterium*	*A. rhizogenes*	5	12	2	3	0	7	0
			*A. tumefaciens*	5	17	3	7	2	5	0
		*Neorhizobium*	*N. galegae*	1	1	1	0	0	0	0
		*Rhizobium*	*R. alamii*	1	3	1	0	0	2	0
			*R. gallicum*	1	4	0	4	0	0	0
			*R. sullae*	1	1	1	0	0	0	0
			*R. tibeticum*	2	4	4	0	0	0	0
		*Shinella*	*S. zoogloeoides*	2	13	12	1	0	0	0
Rhodospirillales	*Rhodospirillaceae*	*Inquilinus*	*I. ginsengisoli*	1	1	0	1	0	0	0
Sphingomonadales	*Sphingomonadaceae*	*Novosphingobium*	*N. barchaimii*	1	1	0	1	0	0	0
		*Sphingobium*	*S. japonicum*	1	1	0	1	0	0	0
			*S*. sp.	1	1	0	1	0	0	0
		*Sphingomonas*	*S. asaccharolytica*	3	3	0	3	0	0	0
			*S. kyeonggiensis*	1	3	0	0	3	0	0
			*S. melonis*	1	1	0	1	0	0	0
			*S. paucimobilis*	1	3	0	3	0	0	0
			*S. pituitosa*	1	2	0	0	2	0	0
Burkholderiales	*Alcaligenaceae*	*Achromobacter*	*A*. sp.	4	5	1	0	4	0	0
	*Burkholderiaceae*	*Burkholderia*	*B. ambifaria*	8	35	3	10	0	22	0
			*B*. sp.	1	1	0	1	0	0	0
		*Paraburkholderia*	*P. phenazinium*	1	1	0	0	0	0	1
			*P. soli*	1	1	0	0	0	0	1
		*Ralstonia*	*R. pickettii*	1	1	0	1	0	0	0
	*Comamonadaceae*	*Variovorax*	*V. paradoxus*	1	1	1	0	0	0	0
	*Oxalobacteraceae*	*Massilia*	*M. haematophila*	1	1	0	0	1	0	0
Enterobacterales	*Enterobacteriaceae*	*Enterobacter*	*E. asburiae*	1	1	1	0	0	0	0
			*E. cloacae*	17	51	19	18	14	0	0
			*E. ludwigii*	9	11	2	6	3	0	0
			*E*. sp.	1	2	2	0	0	0	0
		*Lelliottia*	*L. amnigena*	1	1	1	0	0	0	0
	*Erwiniaceae*	*Pantoea*	*P. ananatis*	1	1	0	1	0	0	0
Pseudomonadales	*Pseudomonadaceae*	*Pseudomonas*	*P. brassicacearum*	3	9	8	1	0	0	0
			*P. graminis*	1	1	0	1	0	0	0
			*P. koreensis*	2	2	0	2	0	0	0
			*P. putida*	2	5	5	0	0	0	0
			*P*. sp.	1	3	0	3	0	0	0
Xanthomonadales	*Rhodanobacteraceae*	*Dyella*	*D. marensis*	2	2	0	2	0	0	0
		*Luteibacter*	*L. rhizovicinus*	4	10	1	1	2	6	0
		*Rhodanobacter*	*R. lindaniclasticus*	1	1	0	0	0	0	1
			*R*. sp.	2	2	0	0	0	0	2
	*Xanthomonadaceae*	*Stenotrophomonas*	*S. maltophilia*	8	12	8	2	2	0	0
Flavobacteriales	*Flavobacteriaceae*	*Chryseobacterium*	*C. daecheongense*	3	7	0	4	3	0	0
			*C. taeanense*	1	2	1	0	1	0	0
Sphingobacteriales	*Sphingobacteriaceae*	*Mucilaginibacter*	*M*. sp.	2	2	1	1	0	0	0
Corynebacteriales	*Corynebacteriaceae*	*Corynebacterium*	*C*. sp.	2	2	1	0	1	0	0
	*Mycobacteriaceae*	*Mycolicibacterium*	*M. frederiksbergense*	2	2	0	1	1	0	0
			*M. gilvum*	1	1	0	0	1	0	0
			*M. hodleri*	1	1	0	1	0	0	0
			*M. llatzerense*	3	4	1	2	1	0	0
			*M. moriokaense*	1	1	0	1	0	0	0
			*M. mucogenicum*	3	7	0	3	4	0	0
			*M. neoaurum*	4	4	0	1	3	0	0
			*M. pallens*	1	3	0	0	3	0	0
			*M. peregrinum*	2	2	0	0	2	0	0
			*M. smegmatis*	1	2	0	2	0	0	0
	*Nocardiaceae*	*Rhodococcus*	*R. erythropolis*	1	1	0	0	1	0	0
		*Nocardia*	*N. nova*	1	2	2	0	0	0	0
Micrococcales	*Cellulomonadaceae*	*Cellulomonas*	*C. hominis*	1	1	1	0	0	0	0
	*Promicromonosporaceae*	*Cellulosimicrobium*	*C. cellulans*	2	3	3	0	0	0	0
	*Microbacteriaceae*	*Leifsonia*	*L. shinshuensis*	9	14	0	3	4	0	7
			*L. soli*	1	1	0	0	1	0	0
			*L. xyli*	4	6	1	2	3	0	0
		*Lysinimonas*	*L*. sp.	3	3	0	2	0	1	0
		*Microbacterium*	*M. oxydans*	2	2	0	1	1	0	0
		*Rathayibacter*	*R. agropyri*	4	7	0	7	0	0	0
			*R. tritici*	1	1	0	0	1	0	0
Micromonosporales	*Micromonosporaceae*	*Micromonospora*	*M*. sp.	2	2	0	0	2	0	0
Streptomycetales	*Streptomycetaceae*	*Streptomyces*	*S. niveus*	1	1	0	1	0	0	0
			*S. mirabilis*	1	1	0	1	0	0	0
Streptosporangiales	*Streptosporangiaceae*	*Microbispora*	*M. rosea*	1	1	0	0	1	0	0
			*M*. sp.	1	1	0	0	1	0	0
Bacillales	*Alicyclobacillaceae*	*Tumebacillus*	*T. luteolus*	2	2	0	0	0	2	0
	*Bacillaceae*	*Bacillus*	*B. altitudinis*	4	4	0	1	0	0	3
			*B. aryabhattai*	3	3	0	0	3	0	0
			*B. cereus*	2	2	2	0	0	0	0
			*B. drentensis*	2	2	0	0	0	0	2
			*B. flexus*	1	1	0	0	0	1	0
			*B. megaterium*	5	7	3	0	3	1	0
			*B. mycoides*	1	2	0	0	0	1	1
			*B. niacini*	1	1	0	1	0	0	0
			*B. pumilus*	6	15	1	0	1	6	7
			*B. simplex*	3	5	0	0	2	2	1
			*B*. sp.	7	41	4	1	14	7	15
			*B. subtilis*	5	12	0	0	8	4	0
			*B. thuringiensis*	3	3	0	0	2	1	0
			*B. velezensis*	2	3	0	2	0	0	1
	*Paenibacillaceae*	*Brevibacillus*	*B. brevis*	1	1	1	0	0	0	0
		*Cohnella*	*C. plantaginis*	5	6	3	0	3	0	0
		*Paenibacillus*	*P. aceris*	1	1	0	0	0	0	1
			*P. agaridevorans*	1	1	0	0	1	0	0
			*P. alginolyticus*	2	2	0	0	2	0	0
			*P. amylolyticus*	1	1	0	0	0	0	1
			*P. anaericanus*	1	1	0	0	1	0	0
			*P. campinasensis*	1	2	0	2	0	0	0
			*P. chondroitinus*	1	1	0	0	1	0	0
			*P. elgii*	1	1	1	0	0	0	0
			*P. glycanilyticus*	1	1	0	0	1	0	0
			*P. graminis*	1	1	1	0	0	0	0
			*P. mucilaginosus*	1	1	0	0	1	0	0
			*P. polymyxa*	1	2	0	0	1	0	1
			*P. provencensis*	3	3	0	0	0	0	3
			*P*. sp.	3	4	2	0	2	0	0
			*P. stellifer*	1	1	0	0	0	0	1
			*P. taichungensis*	2	3	0	1	0	0	2
			*P. terrigena*	1	1	0	1	0	0	0
			*P. validus*	2	3	1	0	0	0	2
			*P. vulneris*	1	1	0	0	1	0	0
			*P. xylanexedens*	6	7	0	0	7	0	0
			*P. xylanilyticus*	1	3	0	3	0	0	0
	*Staphylococcaceae*	*Staphylococcus*	*S. warneri*	1	1	0	0	0	1	0
			Total	260	479	109	123	125	69	53

*Ctrl stands for control plants*.

Tomato roots were thinner than potato roots, thus fewer isolates were recovered from tomato plants (25.47% of the total isolates) using the sterilization procedure. After de-replication, only 15 strains were shared by tomato and potato plants (Supplementary Figure 2B). The bacterial community isolated from potato plants differed considerably to the tomato plants (Figure 1). Interestingly, strains belonging to *Enterobacteriaceae* (overall top second family) were only found in potato roots. Abundances of bacterial taxa isolated from the same plant species, but under different treatment, were more similar. Nevertheless, there were some differences between control plants and plants inoculated with *S. indica* (Table 1 and Figure 1A). Particularly, strains of the genus *Burkholderia* (9 strains, 36 isolates) were only found in control plants of tomato and potato, or in plants inoculated at 10^2^ CFU/g, but never in the treatments of plants heavily (10^4^ CFU/g) inoculated with *S. indica* (Table 1 and Supplementary Figure 2C).

### Interaction Between *S. indica* and Bacteria

From the whole assemblage of bacteria co-cultured *in vitro* with *S. indica*, similar number of strains were found in each type of interaction. Twenty percentage of the total strains were completely inhibitory, 26% negative, 28% neutral, and 26% positive for *S. indica* growth (Figure 1C), revealing that *S. indica* must coexist with antagonistic microbes, but also with synergistic ones, during the process of root colonization by the fungus.

*Bacillaceae, Enterobacteraceae*, and *Burkholderiaceae* were the most detrimental families for *S. indica* growth (Figure 1C). All the strains from *Enterobacteriaceae* displayed an inhibitory or negative interaction with the beneficial fungus. Similar results were obtained with members of *Bacillaceae*, especially strains of *B. subtilis, B. pumilus, B. velezensis*, and *B. thuringiensis*, although strains of *B. simplex* displayed a neutral effect to *S. indica* growth. In the *Burkholderiaceae* family, all the strains of *Burkholderia* completely inhibited *S. indica* growth or were strongly negative, while one strain of *Paraburkholderia* displayed positive interactions (Supplementary Figure 2D). Likewise, the great majority of *Leifsonia, Rathayibacter*, and *Pseudomonas* (95%) strains showed an inhibitory or negative interaction with *S. indica*.

The most abundant families showing a positive interaction were *Mycobacteriaceae* (22.67% of the total positive strains), *Rhizobiaceae* (13.33%), *Xanthomonadaceae* (10.67%), *Paenibacillaceae* (10.67%), and *Rhodanobacteraceae* (9.33%). Considering the type of interaction within a family, every strain of *Xanthomonadaceae*, and 17 out of 19 for *Mycobacteriaceae* stimulated *S. indica* growth. At the genus level, several strains classified as *Achromobacter* and *Sphingomonas* were further positive for *S. indica* growth, although some others were just neutral.

### Screening of Bacterial Strains for Further Experiments

**Figure 2:**
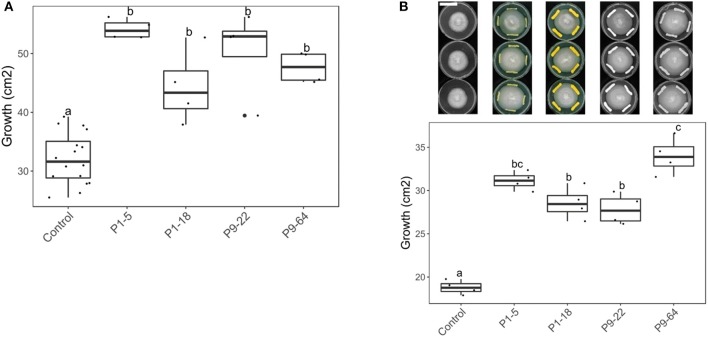
Effect of *Mycolicibacterium* strains on *S. indica* growth (in cm^2^). Co-cultured on PDA, with bacteria pre-cultured for 4 days. Same letters represent non-significantly different mean values, according to Tukey's test (*P* < 0.05), after ANOVA (*n* = 4). **(A)** With *Serendipita indica* and bacteria co-cultured at the same spot (direct contact). **(B)** Bacteria and fungus co-cultured at different spots. Bar in the picture corresponds to 4 cm.

Since *Mycobacteriaceae* was the family that contains more strains stimulating fungal growth, four isolates of the genus *Mycolicibacterium* (P1-5, P1-18, P9-22, and P9-64) recovered from potato roots were selected for further experiments. These isolates strongly stimulated *S. indica* growth when co-cultured on PDA and after 4 days of bacterial pre-culture (Figure 2A). The four isolates further stimulated *S. indica* growth on different growing media (PDA+NA) and bacterial growth phase (9 days preculture) (Supplementary Figure 3), except for P9-22 that did not significantly increase fungal growth on PDA+NA. To rule out the hypothesis that the stimulating effect of bacteria on fungal growth is due to stressed hyphae running away from the bacterium, we further confronted *S. indica* with the selected isolates, but growing on different zones of the Petri dish. Concomitantly, *S. indica* growth was also stimulated when the bacteria were streaked few centimeters away from the fungus (Figure 2B).

### Effect of Combined Inoculation of Endophytic Bacteria and *S. indica* on Tomato Growth

**Figure 3:**
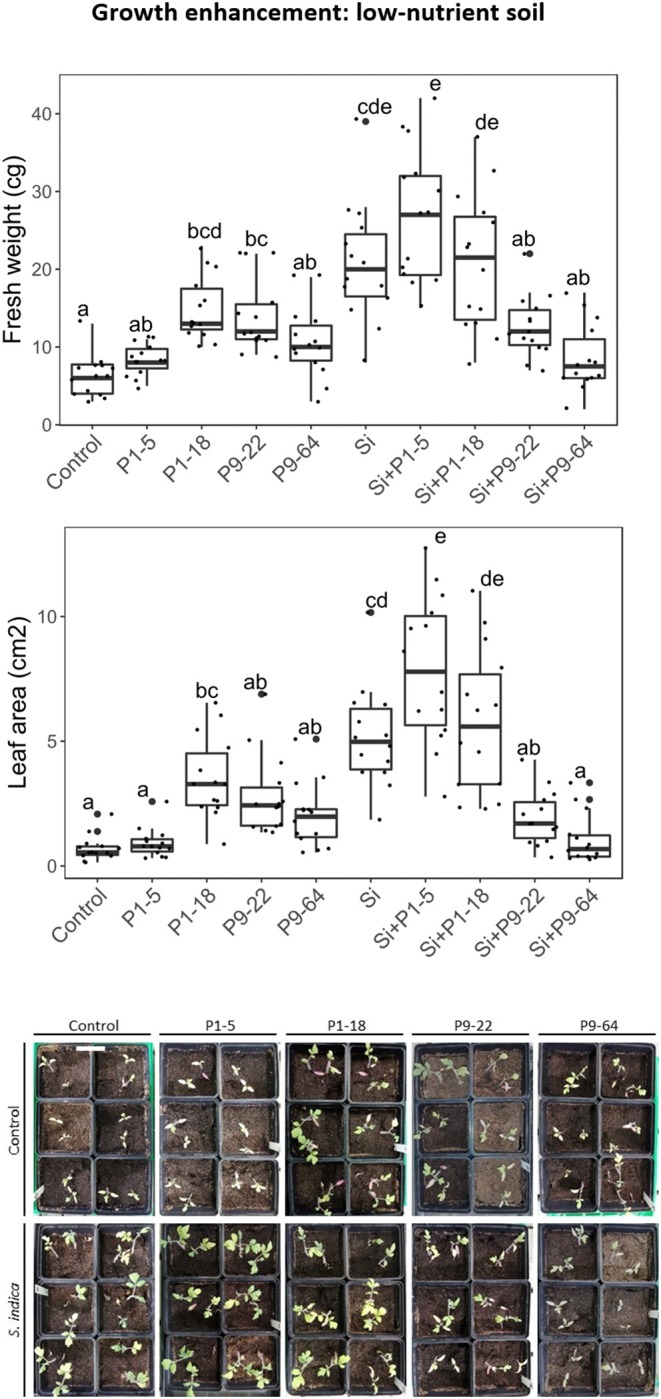
Effect of isolates alone or in combination with *Serendipita indica* on tomato plants under low-nutrient soil conditions. Same letters represent non-significantly different mean values, according to Tukey's test (*P* < 0.05) after ANOVA. *n* = 14. Bar in the picture corresponds to 6 cm.

**Figure 4:**
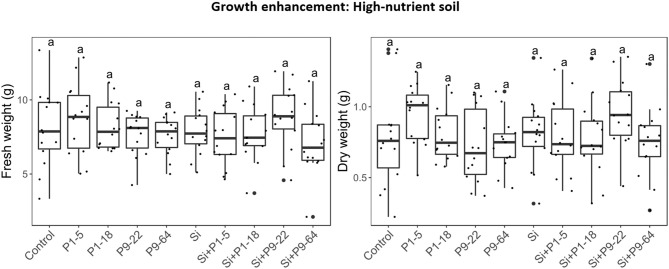
Effect of isolates alone or in combination with *Serendipita indica* on tomato plants under rich soil conditions. Same letters represent non-significantly different mean values, according to Tukey's test (*P* < 0.05) after ANOVA. *n* = 14.

To determine effects of selected *Mycolicibacterium* strains and *S. indica* on plants, tomato plants were inoculated with single or dual inoculations. The fresh weight in all the inoculated treatments was never lower than control (untreated) plants (Figure 3), therefore none of these microbes seemed detrimental or pathogenic for plant growth. Apart from the strain P1-5, single inoculations of bacteria increased plant growth, but only P1-18 and P9-22 increased shoot fresh weight significantly. Inoculation of plants with *S. indica* increased shoot fresh weight (3.3-fold). Dual inoculations of *S. indica*+P1-5 and *S. indica*+P1-18 further enhanced the beneficial effect triggered by *S. indica*, but it resulted significant only for leaf area measurements of plants inoculated with *S. indica*+P1-5. Contrarily, dual inoculations of *S. indica*+P9-22 and *S. indica*+P9-64 displayed lower performance than inoculation of *S. indica* alone. To confirm the plant growth promotion triggered by these microbes, this experiment was repeated in soil with high content of nutrients but in this experiment no differences were obtained between treatments (Figure 4).

### *In vitro* Interaction Between Selected Bacterial Strains and Fungal Pathogens

By *in vitro* dual-culturing, the effect of selected *Mycolicibacterium* strains on *Fusarium* and *Rhizoctonia* growth was studied. In contrast to the beneficial interaction observed between *S. indica* and the selected *Mycolicibacterium* strains, none of these bacteria stimulated *F. oxysporum* growth. Interestingly, some strains significantly reduced hyphal growth respect to the control (Supplementary Figure 4) under certain growth conditions. In the case of *Rhizoctonia solani*, none of the four bacteria restrained fungal growth *in vitro*. Contrarily, the strains P9-22 and P9-64 slightly stimulated fungal growth under certain growth conditions.

### Tomato Protection Against *Fusarium oxysporum* and *Rhizoctonia solani* Using Dual Inoculations

**Figure 5:**
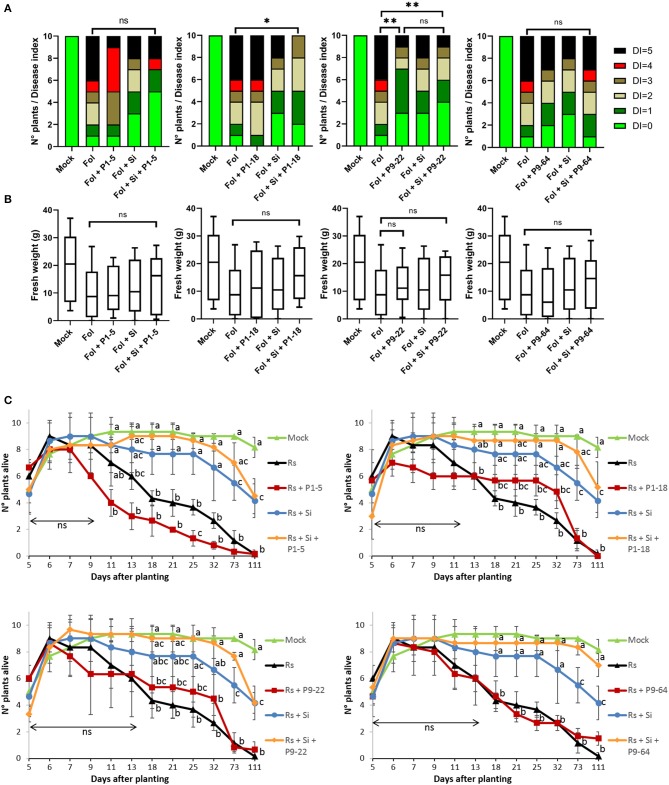
Effect of isolates in combination or not with *Serendipita indica* (Si) on tomato plants against fungal pathogens. **(A,B)** Biocontrol of *Fusarium oxysporum* (Fol). **(A)** Disease index (DI) score. **(B)** Fresh weight (FW). Boxes represent standard deviation with median. Whiskers represent the Min to Max of all the values. Analysis of FW and DI was performed using the non-parametric Mann–Whitney *U*-test (^*^P < 0.05, ^**^P < 0.01, ns = non significant; *n* = 10); **(C)** biocontrol of *Rhizoctonia solani* (Rs). Points represent mean values, and whiskers standard deviations. Same letters represent non-significantly different mean values, according to Tukey's test (*P* < 0.05) after ANOVA (*n* = 3). ns, non significant.

Tomato plants infected with the pathogen *F. oxysporum* (Fol) showed typical symptoms as leaf yellowing, necrotized vessels, wilting, and death (as described in van der Does et al., 2019). On average, fresh weights of *Fusarium*-treated plants were always reduced in comparison with mock-inoculated plants, although it was statistically significant uniquely for plants single-inoculated with P9-64 (Figure 5B). The extent of disease progression (i.e., yellowing, brown bundles, wilting) seemed to be alleviated when the plants were treated with the beneficial fungus *S. indica* but it was not statistically significant. Only during combined treatments of *S. indica*+P1-18 and *S. indica*+P9-22, the level of disease progression was significantly reduced (Figure 5A). Furthermore, single inoculation of P9-22 significantly reduced Fol symptoms to the same extent as the combination *S. indica*+P9-22.

Concerning the experiments with *Rhizoctonia*, the effect of seedlings germinated and alive was regularly monitored up to 111 days. The effects of the damping-off caused by *Rhizoctonia* first appeared 7 days after planting (dap), affecting only treatments in which *S. indica* was not inoculated (Figure 5C). Seedlings inoculated with *S. indica* as well as combinations of *S. indica* + bacteria were not affected by the pathogen until 11 dap. In contrast, single inoculation of *Mycolicibacterium* P1-5 accelerated the damping off caused by *Rhizoctonia* and the number of seedlings alive was significantly decreased 25 dap in comparison to *Rhizoctonia* control. In general, inoculation of seedlings with *S. indica* conferred resistance against the pathogen, but uniquely dual inoculations of *S. indica* + bacteria significantly maintained the number of seedlings alive for all the measurements from 18 to 111 dap, in comparison to *Rhizoctonia* control. The combinations of *S. indica*+P1-18 (at 73 dap) and *S. indica*+P9-64 (at 73 and 111 dap) further significantly increased the number of plants alive in comparisons to single inoculation of *S. indica*.

### Genome Analysis

#### Genomic Features, ANI and Phylogeny of Sequenced Strains

The genomes of the *Mycolicibacterium* strains P1-5, P1-18, P9-22, and P9-64 have a total of 5.47, 6.70, 6.79, and 7.34 Mb with an average G+C content of 65.95, 68.74, 66.89, and 66.27%, respectively. No evidence of plasmids was ascertained. The analysis of antimicrobial resistance or virulence genes detected few genes (Supplementary Table 1). In particular, strain P1-5 shows the presence of the *rbpA* gene, that can confer resistance to rifampin, and the strains P1-18 and P9-64 harbor the gene *tet(V)*, possibly involved in tetracycline resistance. The genomic features of the four genomes are summarized in Table 2. To determine the relatedness of the sequenced strains to genomes publicly available at the NCBI database, ANI was calculated with the BLAST algorithm (ANIb), and with tetranucleotide frequency correlation coefficients (TETRA). The maximum values of ANIb for the four sequenced genomes were only in the range of 80–90% (Supplementary Table 2) and therefore the proposed threshold of ≈95% ANIb as the putative boundary for species circumscriptions was not reached (Konstantinidis and Tiedje, 2005; Richter and Rosselló-Móra, 2009). Contrarily, the TETRA values for the isolates P1-5 and P9-22 reached the 99% threshold (Supplementary Table 2) required to support the species circumscription (Richter and Rosselló-Móra, 2009). However, since TETRA values > 99% should agree with ANIb > 95–96% (Richter and Rosselló-Móra, 2009), we did not assign species names to these isolates.

**Table 2:** Summary of the genomic features of the four *Mycolicibacterium* strains.

**Feature**	**P1-5**	**P1-18**	**P9-22**	**P9-64**
Length (bp)	5,470,684	6,702,551	6,794,647	7,340,553
G + C content (%)	65.95	68.74	66.89	66.27
Contigs	54	35	39	46
Total genes	5,292	6,515	6,609	7,061
Predicted CDS	5,213	6,430	6,522	6,974
rRNA number	2	2	3	2
tRNA number	51	54	54	51
miscRNA number	25	28	29	33
tmRNA number	1	1	1	1
GenBank accession	NPKT00000000	NPKR00000000	NPKP00000000	NPKO00000000

**Figure 6:**
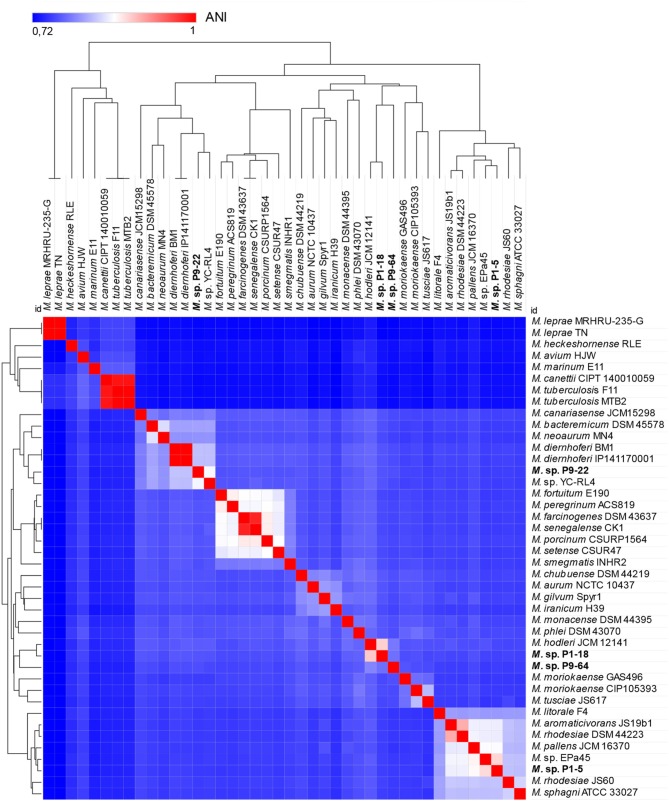
Heat map and dendrogram of average nucleotide identity (ANI) values amongst different strains of *Mycobacteriaceae* showing separation of two groups. The group on the left include common human pathogens and are included in the genus *Mycobacterium*, clade “*Tuberculosis-Simiae*.” The group on the right consists of species of the new genus *Mycolicibacterium*, clade “*Fortuitum-Vaccae*.” The four sequenced strains P1-5, P1-18, P9-22, and P9-64 are included in the group of *Mycolicibacterium* (right side).

The dendrogram computed with the distance matrix of pairwise ANI values showed two separated groups (Figure 6). One group represents the clade of slow-growing mycobacteria designated as “*Tuberculosis-Simiae”* and depicts the emended genus *Mycobacterium* (Gupta et al., 2018; Oren and Garrity, 2018). This group includes well-known human pathogens (Gupta et al., 2018), most notably *Mycobacterium leprae* and *Mycobacterium tuberculosis*, causative agents of leprosy and tuberculosis, respectively (Magee and Ward, 2012; Lory, 2014). The second group, in which the four sequenced bacteria are included, encompasses species from the clade “*Fortuitum-Vaccae*,” recently transferred to a new genus, *Mycolicibacterium* gen. nov. (Oren and Garrity, 2018).

#### Genes and Proteins Predicted to Stimulate *S. Indica* Growth

Analysis of the genomes revealed the four *Mycolicibacterium* strains contain numerous genes predicted to be involved in the stimulation of *S. indica* growth. Some vitamins and cofactors are indispensable for fungal growth (van Overbeek and Saikkonen, 2016), and since some vitamins like cobalamin (B12) can only be synthesized by bacteria (Ghignone et al., 2012; Danchin and Braham, 2017), we hypothesized that production of vitamins could be one of the key factors in the stimulation of fungal growth. We identified several genes involved in the synthesis of six vitamins of the vitamin B complex: cobalamin (B12), biotin (B7), thiamin (B1), riboflavin (B2), pyridoxin (B6), and folate (B9), as well as menaquinone and phylloquinone of the vitamin K complex in all the four genomes. Moreover, genes related to nitrogen metabolism such as those for nitrate and nitrite reductase, ammonification, ammonium transporters, and glutamine synthase were detected in all four genomes (Table 3 and Supplementary Table 3).

**Table 3:** Protein encoding genes predicted to be involved in fungal growth stimulation of strains P1-5, P1-18, P9-22, and P9-64 determined by RAST.

**Category**	**Subsystem**	***Myco***. **P1-5**		***Myco***. **P1-18**		***Myco***. **P9-22**		***Myco***. **P9-64**	
		**n^**°**^R**	**n^**°**^G**	**n^**°**^R**	**n^**°**^G**	**n^**°**^R**	**n^**°**^G**	**n^**°**^R**	**n^**°**^G**
Vitamins/Cofactors	Biotin (vitamin B7) biosynthesis	11	47	12	36	11	51	12	49
	Thiamin (vitamin B1) biosynthesis	8	11	12	12	11	16	10	11
	Menaquinone and Phylloquinone (vitamin K1 and K2) biosynthesis	7	13	6	10	7	12	8	12
	Cobalamin (vitamin B12) synthesis	12	11	24	23	13	12	24	21
	Heme and Siroheme biosynthesis	14	15	14	24	14	18	14	19
	Riboflavin (vitamin B2) metabolism	11	10	12	9	12	10	12	11
	Pyridoxin (vitamin B6) biosynthesis	7	10	7	16	8	20	7	16
	Folate (vitamin B9) biosynthesis	21	27	21	29	21	28	21	29
Cell wall/Secretion	Lipoprotein releasing			1	1				
	Amino acid and peptide ABC transporter	12	14	14	39	14	47	14	47
Nitrogen	Nitrate reductase	5	6	1	2	1	2	5	5
	Nitrite reductase	2	2	2	2	2	4	2	2
	Nitrate/Nitrite transporter	1	3	3	3	4	11	3	9
	Nitric oxide reductase	2	4			3	5	1	1
	Ammonium transporter	1	2	1	2	1	4	1	2
	Glutamine synthetase	2	4	3	6	3	8	4	9
	Glutamate synthase	3	5	3	7	3	8	3	5
Carbohydrates	Trehalose biosynthesis	12	9	10	13	9	11	10	12

*n°R, number of subsystem roles/proteins; n°G, number of protein encoding genes (peg)*.

#### Genes and Proteins Related to Plant Growth Promotion Traits

The RAST annotation and the functional annotation of proteins based on the eggNOG protein database detected various genes related to plant growth promotion (PGP) traits. In the genomes of the strains P1-18, P9-22, and P9-64, we identified key genes attributable to well-known plant growth-promoting compounds like siderophore synthesis and receptors as well as siderophore-interacting proteins, auxin biosynthesis and acetoin and butanediol metabolism (Table 4 and Supplementary Table 4). Regarding strain P1-5, the antiSMASH analysis detected a secondary metabolite cluster identified as mycobactin (Supplementary Table 5), a siderophore used by members of the genus *Mycobacterium* to shuttle free extracellular iron ions into the cytoplasm (McMahon et al., 2012). This cluster was also detected in the genomes of P9-22 and P9-64.

**Table 4:** Protein encoding genes predicted to be involved in plant growth promotion and resistance of strains P1-5, P1-18, P9-22, and P9-64 determined by RAST.

**Category**	**Subsystem**	***Myco***. **P1-5**		***Myco***. **P1-18**		***Myco***. **P9-22**		***Myco***. **P9-64**	
		**n^**°**^R**	**n^**°**^G**	**n^**°**^R**	**n^**°**^G**	**n^**°**^R**	**n^**°**^G**	**n^**°**^R**	**n^**°**^G**
Siderophore/Fe uptake	Siderophore receptors/transport			1	1	5	6	3	3
	Ferrous iron transporter	3	3	4	6	2	2	3	3
Phosphate solubilization	Phosphatase	2	3	3	5	4	6	4	6
	Pyrroloquinoline Quinone biosynthesis							5	5
Phosphate uptake and transport	Low-affinity inorganic phosphate transport system	1	1	1	1	1	1	1	1
	High-affinity phosphate transport system	8	12	8	13	8	12	8	14
	Phosphonate ABC transporter					3	3		
Plant hormone	Auxin biosynthesis			3	5	4	7	5	10
Polyamine	Putrescine/spermidine synthesis	4	3	4	10	8	8	6	11
	Putrescine/spermidine transport	4	4	8	20	7	7	8	23
Resistance to heavy metals	Cobalt-zinc-cadmium resistance	2	5	3	10	3	12	3	7
	Copper homeostasis/tolerance	6	9	8	11	6	12	6	14
	Arsenic resistance	4	9	4	15	4	11	4	10
	Mercury resistance and detoxification	1	2	2	3	1	1	2	5
	Chromium compounds resistance					2	2	1	1
	Uptake of selenate/selenite	2	4	5	11	3	6	5	12
VOC's PGP	Acetoin butanediol metabolism			5	9	6	9	6	12
Resistance to antibiotics	Fluoroquinolone resistance	4	5	4	4	4	4	4	4
	Beta-lactamase	3	9	3	9	3	10	3	10
	Oxalate catabolism			1	1			1	1
	4-hydroxybenzoate degradation			1	1				
Antibiosis compounds	Clavulanic acid biosynthesis	1	1	1	1				
	Chitinase & β-hexosaminidase	2	2	1	1	1	1	1	1
Resistance to oxidative stress	Peroxidase	5	6	6	9	6	8	6	8
	Catalase	1	4	1	10	1	3	1	6
	Superoxide dismutase	1	2	3	3	4	4	4	4
	Hydroperoxide reductase	4	6	3	6	5	7	3	7
	Glutathione-mediated detoxification	2	3	2	3	3	7	2	3
	Mycothiol	10	9	10	9	10	9	10	12
Heat/cold shock	Heat shock protein/chaperone	15	17	15	18	15	18	15	19
	Cold shock protein	2	3	2	2	2	3	2	3
Salt tolerance	Choline/betaine uptake and biosynthesis	7	7	13	33	11	18	10	23
	Ectoine biosynthesis					4	4		
	K+/Na+ transport	15	22	19	27	19	27	19	28
	Trehalose biosynthesis	12	9	10	13	9	11	10	12
Protection from UV radiation and oxidative stress	Mycosporine synthesis			4	4				
	Carotenoids	9	13						

*n°R, number of subsystem roles/proteins; n°G, number of protein encoding genes (peg)*.

The four genomes, and especially P9-64, contain also genes involved in phosphate solubilization (Table 4 and Supplementary Table 4). These genomes encode phosphatases and pyrroloquinoline quinone biosynthesis (*pqqE* genes) that catalyzes the synthesis of gluconic acid, considered as one of the major organic acids responsible for mineral phosphate solubilization (Wagh et al., 2014; Liu et al., 2016). Phosphate solubilization was additionally confirmed *in vitro* (data not shown). Furthermore, inorganic phosphate transport and uptake may be facilitated by low- and high-affinity phosphate transport systems, detected in these genomes. Similarly, iron is an essential nutrient for plant nutrition that is mainly absorbed by plants as ferrous iron (Morrissey and Guerinot, 2009). Genes coding for ferrous iron transporters are present in the four genomes analyzed, contributing to the provision of iron to the plants. Polyamines are phytohormone-like compounds with biological activity in processes like plant growth, development, and stress mitigation (Niemi et al., 2002; Kuznetsov et al., 2006). Furthermore, we identified several proteins involved in the transport and synthesis of the polyamines spermidine and putrescine in all genomes. Moreover, the strains P1-18 and P9-64 contain the gene *acdS* for 1-aminocyclopropane-1-carboxylate (ACC) deaminase (Supplementary Table 6), that enhances plant growth by lowering plant ethylene levels (Glick, 2014).

#### Genes and Proteins Related to Stress Tolerance

The four genomes encode genes implicated in protection against diverse stresses (Table 4 and Supplementary Table 4), and this protection may indirectly lead to growth promotion (Liu et al., 2016). We further found genes involved in resistance to heavy metals and metalloids including cobalt, zinc, cadmium, copper, arsenic, mercury, chromium, and selenite. We also identified numerous proteins and compounds that protect the cell from oxidative stress: peroxidases, catalases, hydroperoxide reductases, superoxide dismutases, glutathione S-transferases, and mycothiol (Newton et al., 2008). The four genomes also encode domain proteins of rhodanese (Supplementary Table 6), an enzyme that detoxifies cyanide (Cipollone et al., 2008), and nitrilases and cyanide hydratases, enzymes with critical roles in plant-microbe interactions for defense, nitrogen utilization, detoxification, and synthesis of plant hormones (Howden and Preston, 2009). Strain P1-18 further contains genes involved in the synthesis of mycosporines (Table 4), secondary metabolites considered to be amongst the strongest natural absorbers of UV radiation and with antioxidative capacities (Oren and Gunde-Cimerman, 2007).

The four strains might potentially confer tolerance to salt stress as they possess several copies of the genes encoding choline dehydrogenase and betaine aldehyde dehydrogenase (Table 4 and Supplementary Table 4), required to produce glycine betaine, one of the most important solutes to face osmolarity fluctuations (Nau-Wagner et al., 2012). The genomes contain also several genes involved in trehalose biosynthesis, a sugar with a protective effect under salt and drought stress (Garg et al., 2002). The strain P9-22 further harbors genes for ectoine (Tables 4, 5), an osmolyte that helps organisms survive extreme osmotic stress (Bernard et al., 1993). Complementarily, all strains also contain various K^+^ transport and Na^+^/H^+^ antiporters that contribute to resist hyperosmotic stress (Liu et al., 2016).

**Table 5:** Distribution and number of biosynthetic gene clusters (BGCs) predicted by antiSMASH analysis.

**Cluster type**	***Myco*.**	***Myco*.**	***Myco*.**	***Myco*.**	**Description**
	**P1-5**	**P1-18**	**P9-22**	**P9-64**	
Cf_saccharide	6	7	4	4	Possible saccharide cluster
Terpene	1	2	2	2	Terpene
Cf_fatty_acid	4	1	2	3	Possible fatty acid cluster
Cf_putative	64	83	93	99	Putative cluster of unknown type
T1pks	4	3	1	3	Type I Polyketide synthase (PKS)
T3pks	2	2	1		Type III Polyketide synthase
Otherks	2				Other Polyketide synthase
T1pks-cf_saccharide-nrps	1	1	1		Type I PKS/saccharide/nrps
T1pks-nrps	1	1	2	2	Type I PKS/saccharide
Nrps		1	2	2	Non-ribosomal peptide synthetase
Arylpolyene		1			Aryl polyene cluster
Bacteriocin	1	1	2	1	Bacteriocin cluster
Ectoine			1		Ectoine cluster
Other	2	5	3	4	Other secondary metabolite protein

#### Resistance to Antibiotics and Production of Antibiotic Compounds

Genes for antibiotic resistance may protect the plant against other pathogenic microbes. The four genomes encode the enzymes β-lactamases, that provide multi-resistance to β-lactam antibiotics such as penicillins (Neu, 1969), genes involved in resistance to the bactericide fluoroquinolone, and genes encoding multidrug resistance proteins (Table 4 and Supplementary Table 6). P1-18 and P9-64 have genes involved in the degradation of oxalate, a compound secreted by fungi to promote their growth and colonization of substrates (Dutton and Evans, 1996), which might contribute to plant defense against pathogenic fungi.

Similarly, we identified several genes involved in the production of antibiotics compounds (Tables 4, 5 and Supplementary Tables 4–6), including bacteriocins, clavulanic acid (Reading and Cole, 1977), aminoglycosides antibiotics (Davies and Wright, 1997) and type IV pili, a bacterial virulence mechanism that appears operational during pathogenesis of fungal hosts (Dörr et al., 1998). Polyketides are secondary metabolites that have antimicrobial properties, including the mycotoxins produced by fungi (Huffman et al., 2010). We identified in the four genomes several enzymes involved in the synthesis of polyketides (Table 5 and Supplementary Tables 5, 6). Among the biosynthetic gene clusters (BGCs) characterized as polyketides synthases (PKS) by antiSMASH analysis, several have been identified as alkylresorcinols, phenolic lipids with the ability to inhibit bacterial and fungal growth (Stasiuk and Kozubek, 2010). Others BGCs characterized as PKS were identified as the antibiotics rifamycin, FK520 (ascomycin), ansamitocin and tetrocarcin A, and as the siderophore griseobactin (Patzer and Braun, 2010) (Supplementary Table 5). However, the percentage of gene match was very low in comparison to their homologs BGCs, suggesting that these BGCs might encode novel antibiotic and siderophore biosynthetic pathways (de Los Santos-Villalobos et al., 2018). Other antibiotic BGCs detected by antiSMASH with antifungal properties, although with low percentage of gene match, include galbonolides (Fauth et al., 1986) identified in all the genomes, bacillomycin (Gu et al., 2017) in the genomes of P9-22 and P9-64, angucycline Sch 47554 (Basnet et al., 2006), pimaricin (Aparicio et al., 2016) in P1-18, and fengycin (Vanittanakom et al., 1986) in P1-5. We further identified proteins involved in the synthesis of phenazines (Supplementary Table 6), heterocyclic compounds that have been shown to control a wide range of plant pathogenic fungi (Chin-A-Woeng et al., 2003) and to elicit ISR (Pierson and Pierson, 2010).

In addition, these bacteria contain genes encoding enzymes involved in the degradation of the fungal cell-wall, like β-hexosaminidases and chitooligosacharide deacetylases, which have been proved to degrade chitin and chitooligosaccharides (Barber and Ride, 1989; Zhao et al., 2010). Furthermore, genes encoding chitinases are present in the genome of P1-5 (Tables 4, 6) and these enzymes can potentially contribute to biocontrol of fungal pathogens by disruption of fungal cell walls (Whipps, 2001).

**Table 6:** Plant and microbe cell-wall polysaccharide degrading enzymes (CE and GH classes) of strains P1-5, P1-18, P9-22, and P9-64 based on genome analysis.

**CAZy family**	**Substrate**	**Annotation**	**EC number**	**Copy number**			
				**P1-5**	**P1-18**	**P9-22**	**P9-64**
CE1	Polysaccharide	diacylglycerol O-acyltransferase	2.3.1.20	7	8	9	10
		esterase	3.1.1.-				
CE4	Chitooligosaccharide	chitooligosaccharide deacetylase	3.5.1.-	1	2	2	2
		allantoinase	3.5.2.5				
CE5	Cutin	cutinase	3.1.1.74	6	1	9	
CE9	Polysaccharides	N-acetylglucosamine 6-phosphate deacetylase	3.5.1.25	1	1	1	1
CE14	Polysaccharides	N-acetyl-1-D-myo-inosityl-2-amino-2-deoxy-α-D-glucopyranoside deacetylase	3.5.1.89	2	2	2	2
GH1	Cellulose	β-glucosidase	3.2.1.21		1	1	2
	Pectin (rhamnogalacturonan I)	β-galactosidase	3.2.1.23				
GH2	Pectin (rhamnogalacturonan I)	β-glucuronidase	3.2.1.31	2	1	2	2
		β-galactosidase	3.2.1.23				
GH3	Cellulose	β-glucosidase	3.2.1.21	1	3	3	1
GH4	Polysaccharides	α-glucosidase	3.2.1.20	1	2	3	4
	Hemicellulose (galactomannan)	α-galactosidase	3.2.1.22				
GH5	Hemicellulose (xylan)	endo-β-1,4-xylanase	3.2.1.8	1	2	3	2
	Glycosphingolipids	endoglycoceramidase	3.2.1.123				
GH6	Cellulose	endo-β-1,4-glucanase	3.2.1.4	1	1	1	1
GH8	Cellulose	cellulase	3.2.1.4	1		1	
GH13	Polysaccharides	α-amylase	3.2.1.1	5	7	5	6
		α-glucosidase	3.2.1.20				
		trehalose synthase	5.4.99.15				
		α-1,4-glucan: phosphate α-maltosyltransferase	2.4.99.16				
GH15	Polysaccharides	glucoamylase	3.2.1.3	3	3	2	2
		α,α-trehalase	3.2.1.28				
GH16	Polysaccharides	endo-1,3-β-glucanase	3.2.1.39	6	2	1	3
GH17	Chitin	chitinase	3.2.1.14	1			
GH23	Peptidoglycans	peptidoglycan lyase	4.2.2.1	3	2	3	4
GH27	Hemicellulose (galactomannan)	α-galactosidase	3.2.1.22		1		1
GH38	Oligosaccharides	α-mannosidase	3.2.1.24	1	1	1	1
GH39	Cellulose	cellulase	3.2.1.4		3		1
	Pectin (rhamnogalacturonan I)	β-galactosidase	3.2.1.23				
GH57	Polysaccharides	1,4-α-glucan branching enzyme	2.4.1.18	1	1	1	1
GH65	Polysaccharides	trehalose phosphorylase	2.4.1.64	3	2	2	3
		maltose phosphorylase	2.4.1.8				
GH77	Polysaccharides	4-α-glucanotransferase	2.4.1.25	2	2	1	1

*The numbers refer to the number of genes found in each CAZy family*.

#### CAZy Analysis

We identified roughly 140 putative genes encoding carbohydrate-active enzymes (CAZy) in each genome analyzed (Table 7). CAZy were distributed unevenly among the six CAZy families and no enzymes belonged to the class polysaccharide lyases (PL). In the classes carbohydrate esterases (CE) and glycoside hydrolases (GH), we identified several plant cell-wall degrading enzymes (PCWDEs) related genes in all the genomes analyzed (Table 6 and Supplementary Table 7). The PCWDEs identified have the potential to degrade many plant cell-wall polymers, including cellulase, hemicellulose, pectin, and cutin. In addition, P1-18 encodes proteins involved in lignin degradation (Supplementary Table 6). Many other genes related to enzymes involved in the degradation of different plant intracellular polysaccharides were detected (Table 6), suggesting that the strains harbor traits for endophytic colonization.

**Table 7:** Genes related to carbohydrate-active enzymes (CAZymes) in strains P1-5, P1-18, P9-22, and P9-64.

**Strain**	**CBM**	**CE**	**GH**	**GT**	**PL**	**AA**
P1-5	4	18	46	76	0	0
P1-18	4	15	43	72	0	3
P9-22	5	25	54	59	0	1
P9-64	4	17	49	71	0	2

*CBM, Carbohydrate-Binding Module; CE, Carbohydrate Esterase; GH, Glycoside Hydrolase; GT, Glycosyl Transferase; PL, Polysaccharide lyases; AA, Auxiliary Activity*.

## Discussion

In our study, we confirm that the endophyte *S. indica* coexists with communities of deleterious, neutral and beneficial bacteria inside roots as revealed by *in vitro* assays, and there seems to be an ecological balance among these microbial communities (Varma et al., 2012). *Bacillaceae, Enterobacteraceae*, and *Burkholderiaceae* were the most detrimental families for *S. indica* growth. These results are congruent with the widely reported antifungal properties of several strains of *Bacillus* and *Burkholderia* (Compant et al., 2005; de Los Santos-Villalobos et al., 2018). In contrast, many *Rhizobiaceae* strains representing different species and genera stimulated *S. indic*a growth *in vitro*. Intriguingly, it has been confirmed that *S. indica* hosts an endobacterium of *Rhizobium radiobacter* inside its hyphae and the bacterium increases host fitness (Sharma et al., 2008; Glaeser et al., 2016). Hence, it might be that this interaction is not specific and several *Rhizobium* relatives may promote *S. indica* growth. It is not known whether different rhizobia can colonize the fungus internally and it also remains unclear if the isolated *Rhizobiaceae* strains, rather than stimulating the fungus directly, stimulate the activity of the endobacterium of *S. indica* leading to improved growth. Likewise, several isolates identified as *Paenibacillus* exhibited a positive or neutral interaction with *S. indica*. These results are in line with Hildebrandt et al. (2006) who demonstrated that the bacterium *Paenibacillus validus* stimulates growth of the AMF *Glomus intraradices*. Moreover, one of the few endofungal bacteria detected in the *Serendipita* (= *Sebacina*) *vermifera* complex belongs to *Paenibacillus* (Sharma et al., 2008), suggesting a possible synergistic interaction between some strains of *Paenibacillus* and *Serendipita*. The fact that rhizobia and *Paenibacillus* inoculants are frequently applied as biofertilizers (Sessitsch et al., 2002; Grady et al., 2016), suggests that strains of these taxa could be further tested for application jointly with *S. indica*.

Most striking were the effects of *Mycolicibacterium* strains, which highly stimulated *S. indica* growth. This genus is only poorly understood regarding its interaction with plants, although some strains have been tested as bioinoculants owing to plant growth promotion effects (Egamberdiyeva, 2007). The predicted traits from the genomes of the four *Mycolicibacterium* strains revealed the presence of many genes responsible for vitamin production, which are potentially relevant for supplying vitamins to *S. indica* and thereby enhancing its growth. It has been reported that endosymbionts of mycorrhizal fungi are important for the provision of vitamin B12 (cobalamin) to their host (Ghignone et al., 2012), and vitamin B1 (thiamin) is implicated in the growth-promoting effect of *Pseudomonas fluorescens* on the ectomycorrhizal fungus *L. bicolor* (Deveau et al., 2010). Moreover, it has been described that *S. indica* possesses biotroph-associated genomic adaptations, such as lacking genes related to nitrogen metabolism and therefore suffering from some metabolic deficiencies. Congruent with this, *S. indica* barely grows on nitrate, but shows good growth on ammonium and glutamine as N source (Zuccaro et al., 2011). In accordance with these studies, we found several genes coding for nitrate and nitrate reductase as well as amino acid and peptide ABC transporters (genes families of proteins that have undergone contraction in *S. indica* genome) in all four *Mycolicibacterium* genomes. The strains also encode ammonium transporters and glutamine synthase, an enzyme that plays an essential role in the metabolism of nitrogen by catalyzing the condensation of glutamate and ammonia to form glutamine. These genomic features might be involved in increasing supply of glutamine and ammonium to the fungus, complementing the predicted metabolic deficiencies. Furthermore, it has been identified that trehalose is involved in the stimulation of hyphal growth of mycorrhizal fungus (Duponnois and Kisa, 2006; Hildebrandt et al., 2006). The *Mycolicibacterium* strains tested in this study encode genes for synthesis of trehalose, a compound that can be present as a disaccharide in the cytoplasm, but it is also present in the cell-wall glycolipids of Mycobacteria (Argüelles, 2000). The secretion of this sugar and the degradation of cell-wall glycolipids into oligosaccharides might also contribute to *S. indica* growth stimulation. To this point, there is no clear evidence, if only one, or a combination of the above-mentioned bacterial traits are responsible for the positive interactions with *S. indica*.

The four isolates contain many genes related to plant growth promotion traits. As predicted by the analysis of the genomes, strains P1-18, P9-22, and P9-64 enhanced plant growth, while strain P1-5 did not improve plant growth. A plausible reason is that, unlike the other strains, P1-5 lacks some of the most well-known genes involved in PGP, like those for biosynthesis of auxin, the volatile acetoin and ACC deaminase, besides harboring fewer number of genes involved in siderophore and phosphatases production as well as nitrilases, that have a potential role in the biosynthesis of indole-3-acetic acid (auxin) (Park et al., 2003). *S. indica* has been extensively shown to increase plant growth in different crops (reviewed in Franken, 2012). Consistent with this, in our study the inoculation of tomato plants with *S. indica* increased shoot fresh weight and leaf area.

Considering the PGP traits of the *Mycolicibacterium* strains tested in this study, a dual inoculation of *S. indica* with these bacteria can potentially increase plant growth. However, only dual inoculations of the strains P1-5 and P1-18 with *S. indica* moderately enhanced plant growth in comparison to single inoculations. These results are in agreement with Sarma et al. (2011) and Kumar et al. (2012), who observed plant growth promotion by combining pseudomonads and *S. indica*, and concomitantly with our results, the dual inoculation increased only slightly in comparison to single inoculations of each microbe. Interestingly, the strain P1-5 did not enhance plant growth when single-inoculated but was notably effective in plant growth promotion when applied in combination with *S. indica*. This synergistic effect can be ascribed to cooperation in the supply of phosphorus and nitrogen to the plant. These bacteria and *S. indica* encode genes involved in the provision of P to the plant, but it has been controversially discussed if, and how, *S. indica* supplies P to the plant. It has been shown that the fungus is able to solubilize phosphate from inorganic, but not from organic P sources (Ngwene et al., 2016), but also that *S indica* is not involved in the phosphate transfer to host plant (Achatz et al., 2010). The inconsistency of these results reveals how complex the interaction is, and the outcome might be dependent on abiotic factors, like adequate pH. In this regard, the combined effect of bacterial and fungal phosphatases and phosphate transport systems under certain pH levels triggered by the presence of both microbes might be the key factors to plant P uptake and growth promotion. Similarly, the fact that P1-5 and P1-18 possess genes for nitrate reductase, an enzyme which plays a key role in nitrate acquisition in plants (Gill et al., 2016), and for ferrous iron transport, might complement the nitrogen and iron supply to the host plant. Contrarily, co-inoculation of the strains P9-22 and P9-64 with *S. indica* displayed lower performance than single inoculations. These results coincide with Sarma et al. (2011), in which dual inoculation of *S. indica* and the pseudomonad R62 was more detrimental than R62 and *S. indica* inoculated singly. Some authors claim that the negative interaction might be due to niche competition for both space and nutrients as described by Whipps (2001). Similarly, this incompatibility might also be ascribed to alterations in the IAA (auxin) levels of the plants. Provision of low levels of IAA stimulate plant growth whereas high concentration of IAA may influence plant growth negatively (Sarwar and Frankenberger, 1994). *S. indica* can produce auxins, and these strains harbor the largest number of genes involved in auxin biosynthesis, therefore the dual inoculation might lead to auxin overproduction and the consequent imbalance of IAA levels, causing less growth promotion than each microbe inoculated singly.

Furthermore, our study reveals that the beneficial effect of the endophytes on plant growth is dependent on the cultivation substrate. Dual and single inoculation under nutrient-rich conditions did not exhibit shoot growth promotion 6 weeks after planting while on low-nutrient soil it did. Concomitant with our results, it has been shown that under certain nutrient conditions and plant stage *S. indica* and PGPR do not exert beneficial effects (Egamberdiyeva, 2007; Fakhro et al., 2010; Gill et al., 2016) and the outcome of fungal-bacteria interactions can be different from mutualistic to antagonistic. For example, Gorka et al. (2019) recently reported that the interaction between ectomycorrhiza and soil bacteria responded negatively to soil nitrogen application.

*S. indica* has been shown to confer resistance against several fungal pathogens (Waller et al., 2005; Qiang et al., 2012) including *Fusarium* (Deshmukh and Kogel, 2007; Sarma et al., 2011; Rabiey et al., 2015) and *Rhizoctonia* (Knecht et al., 2010), through activation of the antioxidant system (Prasad et al., 2013), defense related genes (such as *PR, LOX2*, and *ERF1*) (Zarea et al., 2012) and ISR. In agreement, *S. indica* always alleviated to some extent the symptoms caused by the fungal pathogens in these experiments. Bacteria employ many different mechanisms involved in biocontrol of fungal pathogens (Compant et al., 2005). Two of the most important mechanisms of biocontrol are the production of siderophores, depriving pathogenic fungi from iron acquisition (Kobayashi and Crouch, 2009), and production of antibiotics and fungal cell-wall degrading enzymes (Whipps, 2001). The genomes of *Mycolicibacterium* strains tested in this study encode genes for siderophore synthesis and receptors, as well as several antimicrobial compounds, including antibiotics, polyketides, phenolic lipids (alkylresorcinols), phenazines, and chitinolytic enzymes, confirming the genetic potential of these strains to exhibit biocontrol effects against pathogenic fungi. However, single inoculations of these bacteria did not protect the plants from pathogen attack. This might not be surprising considering the bacteria did not show *in vitro* direct antagonism to *Fusarium* and *Rhizoctonia*. Furthermore, inoculation of tomato seedlings with the strain P1-5 increased the negative effect caused by *Rhizoctonia*. A possible explanation is that in the same way the bacterium stimulates *S. indica* growth, it stimulates also the growth of *Rhizoctonia* enhancing damping off. Moreover, the bacterium P1-5 might help to detoxify the plant material after pathogen attack (Howden and Preston, 2009), by production of cyanide hydratases, carotenoids and antioxidants. Only strain P9-22 alleviated the symptoms of Fusarium wilt disease when single-inoculated. In reference to its genome, this biocontrol effect might stem from activation of ISR and competition for iron, as this strain encodes the highest number of genes implicated in siderophore synthesis and receptors (including BGCs identified as mycobactin, coelichelin, scabichelin) and numerous genes involved in triggering ISR (phenolic lipids—alkylresorcinols, cell wall-degrading enzymes, polyketides, antibiotics), as well as from direct antagonisms, like secreting the antifungal bacillomycin.

Overall, in this study dual inoculations of *S. indica* and bacteria enhanced the protective effect conferred by *S. indica* against the two pathogens. The strains that better performed were P1-18 and P9-22 against *Fusarium*, and P9-22 and P9-64 against *Rhizoctonia*. Similar results showing synergism by dual-inoculations have been earlier reported (Whipps, 2001). For instance, dual inoculation of *S. indica* and pseudomonad R81 improved biocontrol of *Fusarium* compared to single inoculations (Sarma et al., 2011). This could be explained by cooperation in triggering the plant ISR. According to its biotrophic lifestyle, *S. indica* lacks also genes potentially involved in biosynthesis of toxic secondary metabolites and cyclic peptides, and family proteins involved in PKS and NRPS are contracted (Zuccaro et al., 2011). This deficiency could be ameliorated by these bacterial helpers, as they possess several genes involved in secondary metabolite production, including PKS and NRPS. These secondary metabolites might supplement *S. indica* metabolism, bioenergetic capacity, activation of defense related genes and production of antibiosis compounds (Bonfante and Anca, 2009; Bhuyan et al., 2015; Salvioli et al., 2016), that ultimately raises the plant ISR. These strains possess also genes that might cooperate in restraining pathogen expansion, like strains P1-18 and P9-64 that might degrade oxalate produced by pathogens. Moreover, competition for niche and nutrients (carbon, nitrogen, and iron) has been shown to be a mechanism associated with biocontrol or suppression of Fusarium wilt in several systems (Whipps, 2001). All in all, perhaps the combined effect of bacterial and *S. indica*-mediated ISR, bacterial production of siderophores and antimicrobial compounds [e.g., polyketides, non-ribosomal peptides, phenazines, chitinolytic enzymes, bacillomycin (P9-22 and P9-64), angucycline and pimaricin (P1-18), galbonolides] and the nutrient and niche competition between the pathogen and beneficial microbes might explain the enhanced resistance of plants inoculated with *S. indica* + *Mycolicibacterium*.

These results demonstrate the potential of *Mycolicibacterium-S. indica* combinations for biocontrol of plant pathogens, but the safety of these bacteria should be carefully addressed. As the dendrogram shows, these strains are separated from the clade “*Tuberculosis-Simiae*,” and included in the clade “*Fortuitum-Vaccae*,” primarily comprised of environmental species (Gupta et al., 2018). Besides, the analysis of antimicrobial resistance or virulence genes detected very few antimicrobial resistance genes of concern and the most related strains were classified as risk 1 according to the German classification TRBA. Nevertheless, the fact that these isolates are not related to the well-known human pathogens does not imply that they are completely safe. Moreover, future studies must consider how the application of fungal and bacterial inoculants affect soil microbial communities. For example, several studies provided evidence that mycorrhizal fungi modify the bacterial communities in the rhizosphere (Nuccio et al., 2013). Similarly, Meena et al. (2010) showed that *S. indica* affected population dynamics of pseudomonads in chickpea and also Nautiyal et al. (2010) observed changes in the microbial community structure in soil inoculated with *S. indica*. Most probably, the previously reported shifts in the bacterial communities might be attributed to the modified plant physiology, altered composition of root exudates and changes in the pH (Linderman, 1992; Barea et al., 2005; Svenningsen et al., 2018). Future research is needed in culture independent analysis to study the effect of *S. indica* in the native soil populations, and in exploring the interaction between *S. indica* and other positive strains belonging to different taxa that were isolated in this study. This might help to better understand bacteria-fungal interactions, and the selection of compatible microbial strains for field application, aiming at crop enhancement and biocontrol of fungal pathogens.

## Data Availability Statement

The datasets generated for this study can be found in the Sequence data are available at NCBI database and GenBank under the accession numbers MN180888–MN181366. The draft genome sequences for the *Mycolicibacterium* strains P1-5, P1-18, P9-22, and P9-64 are available at NCBI, BioProject PRJNA393298, with the DDBJ/ENA/GenBank accession numbers NPKT00000000, NPKR00000000, NPKP00000000, and NPKO00000000, respectively.

## Author Contributions

AB-D carried out the experiments and wrote the manuscript. JL and ASa helped for analysis. LA was responsible of genome assemblage and statistics. AB-D, ASe, and SC designed the experiments. All authors gave intellectual input and critically revised the manuscript.

### Conflict of Interest

The authors declare that the research was conducted in the absence of any commercial or financial relationships that could be construed as a potential conflict of interest.

## Footnotes

^1^FastQC. https://www.bioinformatics.babraham.ac.uk/projects/fastqc/
