# Supplementary material for: Beneficial Endophytic Bacteria-Serendipita indica Interaction for Crop Enhancement and Resistance to Phytopathogens
Source: Front Microbiol. 2019 Dec 19;10:2888. doi: 10.3389/fmicb.2019.02888 (PMC6930893; doi:10.3389/fmicb.2019.02888)
Supplement: Supplementary file 1 [file Presentation_1.pptx]

## Slide 1
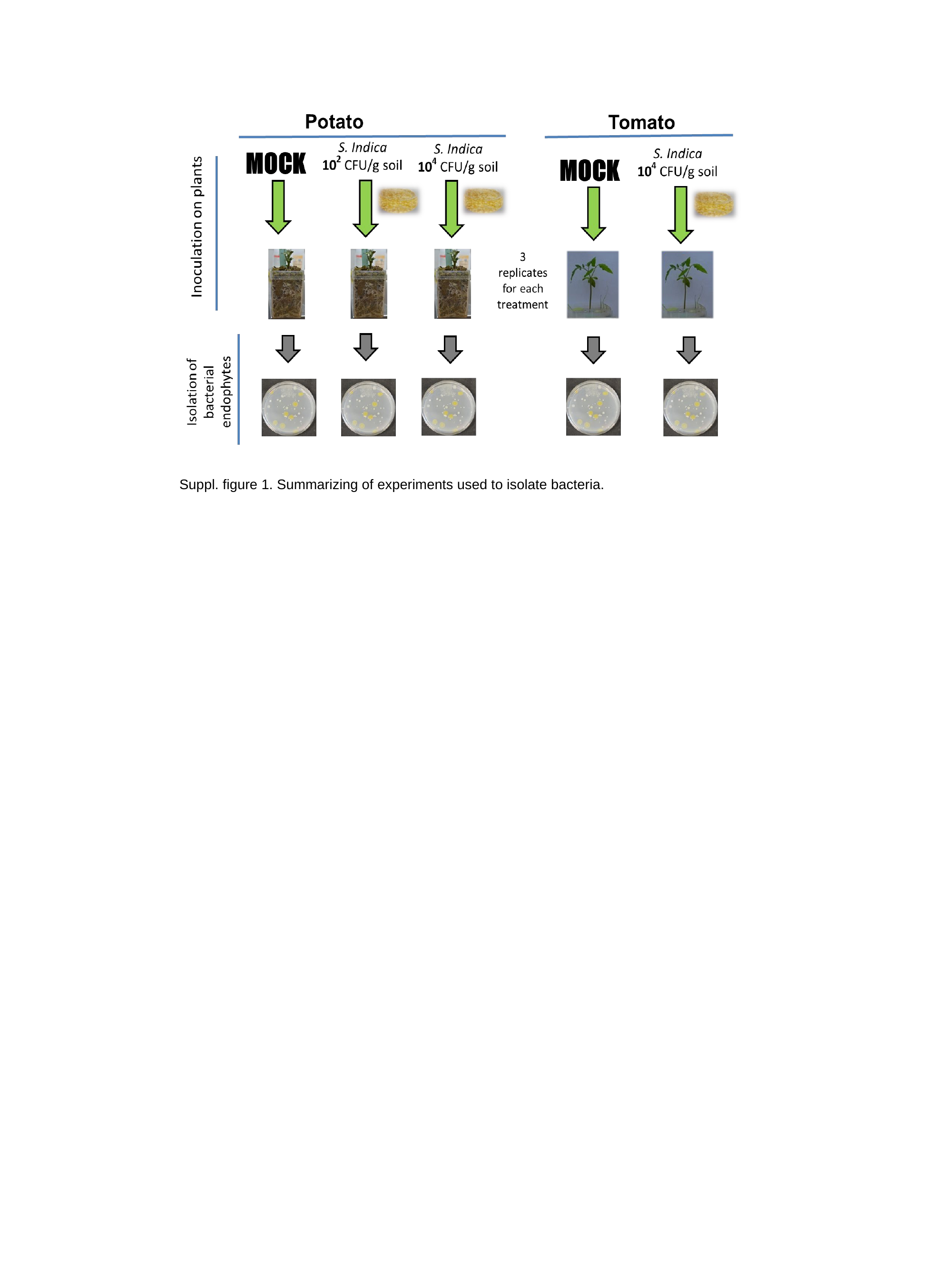

Suppl. figure 1. Summarizing of experiments used to isolate bacteria.

## Slide 2
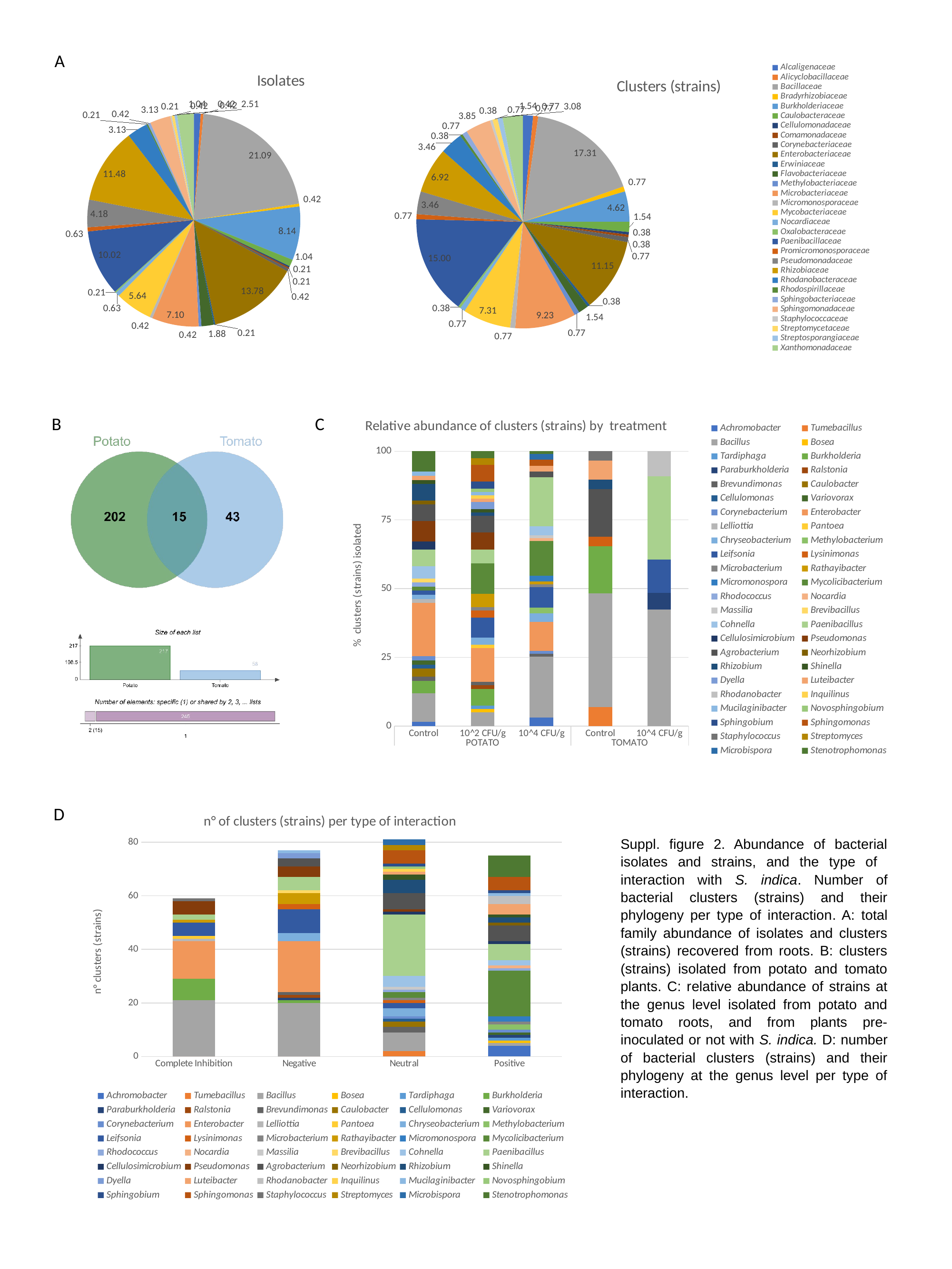

A
### Chart: Isolates
| Category | |
|---|---|
| Alcaligenaceae | 1.0438413361169103 |
| Alicyclobacillaceae | 0.41753653444676403 |
| Bacillaceae | 21.08559498956159 |
| Bradyrhizobiaceae | 0.41753653444676403 |
| Burkholderiaceae | 8.1419624217119 |
| Caulobacteraceae | 1.0438413361169103 |
| Cellulomonadaceae | 0.20876826722338201 |
| Comamonadaceae | 0.20876826722338201 |
| Corynebacteriaceae | 0.41753653444676403 |
| Enterobacteriaceae | 13.778705636743215 |
| Erwiniaceae | 0.20876826722338201 |
| Flavobacteriaceae | 1.8789144050104383 |
| Methylobacteriaceae | 0.41753653444676403 |
| Microbacteriaceae | 7.09812108559499 |
| Micromonosporaceae | 0.41753653444676403 |
| Mycobacteriaceae | 5.6367432150313155 |
| Nocardiaceae | 0.6263048016701461 |
| Oxalobacteraceae | 0.20876826722338201 |
| Paenibacillaceae | 10.020876826722338 |
| Promicromonosporaceae | 0.6263048016701461 |
| Pseudomonadaceae | 4.175365344467641 |
| Rhizobiaceae | 11.482254697286013 |
| Rhodanobacteraceae | 3.1315240083507305 |
| Rhodospirillaceae | 0.20876826722338201 |
| Sphingobacteriaceae | 0.41753653444676403 |
| Sphingomonadaceae | 3.1315240083507305 |
| Staphylococcaceae | 0.20876826722338201 |
| Streptomycetaceae | 0.41753653444676403 |
| Streptosporangiaceae | 0.41753653444676403 |
| Xanthomonadaceae | 2.5052192066805845 |
### Chart: Clusters (strains)
| Category | |
|---|---|
| Alcaligenaceae | 1.5384615384615385 |
| Alicyclobacillaceae | 0.7692307692307693 |
| Bacillaceae | 17.307692307692307 |
| Bradyrhizobiaceae | 0.7692307692307693 |
| Burkholderiaceae | 4.615384615384616 |
| Caulobacteraceae | 1.5384615384615385 |
| Cellulomonadaceae | 0.38461538461538464 |
| Comamonadaceae | 0.38461538461538464 |
| Corynebacteriaceae | 0.7692307692307693 |
| Enterobacteriaceae | 11.153846153846155 |
| Erwiniaceae | 0.38461538461538464 |
| Flavobacteriaceae | 1.5384615384615385 |
| Methylobacteriaceae | 0.7692307692307693 |
| Microbacteriaceae | 9.230769230769232 |
| Micromonosporaceae | 0.7692307692307693 |
| Mycobacteriaceae | 7.307692307692308 |
| Nocardiaceae | 0.7692307692307693 |
| Oxalobacteraceae | 0.38461538461538464 |
| Paenibacillaceae | 15.0 |
| Promicromonosporaceae | 0.7692307692307693 |
| Pseudomonadaceae | 3.4615384615384617 |
| Rhizobiaceae | 6.923076923076923 |
| Rhodanobacteraceae | 3.4615384615384617 |
| Rhodospirillaceae | 0.38461538461538464 |
| Sphingobacteriaceae | 0.7692307692307693 |
| Sphingomonadaceae | 3.8461538461538463 |
| Staphylococcaceae | 0.38461538461538464 |
| Streptomycetaceae | 0.7692307692307693 |
| Streptosporangiaceae | 0.7692307692307693 |
| Xanthomonadaceae | 3.076923076923077 |B
C
### Chart: Relative abundance of clusters (strains) by treatment
| Category | Achromobacter | Tumebacillus | Bacillus | Bosea | Tardiphaga | Burkholderia | Paraburkholderia | Ralstonia | Brevundimonas | Caulobacter | Cellulomonas | Variovorax | Corynebacterium | Enterobacter | Lelliottia | Pantoea | Chryseobacterium | Methylobacterium | Leifsonia | Lysinimonas | Microbacterium | Rathayibacter | Micromonospora | Mycolicibacterium | Rhodococcus | Nocardia | Massilia | Brevibacillus | Cohnella | Paenibacillus | Cellulosimicrobium | Pseudomonas | Agrobacterium | Neorhizobium | Rhizobium | Shinella | Dyella | Luteibacter | Rhodanobacter | Inquilinus | Mucilaginibacter | Novosphingobium | Sphingobium | Sphingomonas | Staphylococcus | Streptomyces | Microbispora | Stenotrophomonas |
|---|---|---|---|---|---|---|---|---|---|---|---|---|---|---|---|---|---|---|---|---|---|---|---|---|---|---|---|---|---|---|---|---|---|---|---|---|---|---|---|---|---|---|---|---|---|---|---|---|
| Control | 1.4925373134328357 | 0.0 | 10.44776119402985 | 0.0 | 0.0 | 4.477611940298507 | 0.0 | 0.0 | 1.4925373134328357 | 2.9850746268656714 | 1.4925373134328357 | 1.4925373134328357 | 1.4925373134328357 | 19.402985074626866 | 1.4925373134328357 | 0.0 | 1.4925373134328357 | 0.0 | 1.4925373134328357 | 0.0 | 0.0 | 0.0 | 0.0 | 1.4925373134328357 | 1.4925373134328357 | 0.0 | 0.0 | 1.4925373134328357 | 4.477611940298507 | 5.970149253731343 | 2.9850746268656714 | 7.462686567164178 | 5.970149253731343 | 1.4925373134328357 | 5.970149253731343 | 1.4925373134328357 | 0.0 | 1.4925373134328357 | 0.0 | 0.0 | 1.4925373134328357 | 0.0 | 0.0 | 0.0 | 0.0 | 0.0 | 0.0 | 7.462686567164178 |
| 10^2 CFU/g | 0.0 | 0.0 | 4.938271604938271 | 1.2345679012345678 | 1.2345679012345678 | 6.172839506172839 | 0.0 | 1.2345679012345678 | 1.2345679012345678 | 0.0 | 0.0 | 0.0 | 0.0 | 12.345679012345679 | 0.0 | 1.2345679012345678 | 2.4691358024691357 | 0.0 | 7.4074074074074066 | 2.4691358024691357 | 1.2345679012345678 | 4.938271604938271 | 0.0 | 11.11111111111111 | 0.0 | 0.0 | 0.0 | 0.0 | 0.0 | 4.938271604938271 | 0.0 | 6.172839506172839 | 6.172839506172839 | 0.0 | 1.2345679012345678 | 1.2345679012345678 | 2.4691358024691357 | 1.2345679012345678 | 0.0 | 1.2345679012345678 | 1.2345679012345678 | 1.2345679012345678 | 2.4691358024691357 | 6.172839506172839 | 0.0 | 2.4691358024691357 | 0.0 | 2.4691358024691357 |
| 10^4 CFU/g | 3.1578947368421053 | 0.0 | 22.105263157894736 | 0.0 | 0.0 | 0.0 | 0.0 | 0.0 | 1.0526315789473684 | 0.0 | 0.0 | 0.0 | 1.0526315789473684 | 10.526315789473683 | 0.0 | 0.0 | 3.1578947368421053 | 2.1052631578947367 | 7.368421052631578 | 0.0 | 1.0526315789473684 | 1.0526315789473684 | 2.1052631578947367 | 12.631578947368421 | 0.0 | 1.0526315789473684 | 1.0526315789473684 | 0.0 | 3.1578947368421053 | 17.894736842105264 | 0.0 | 0.0 | 2.1052631578947367 | 0.0 | 0.0 | 0.0 | 0.0 | 2.1052631578947367 | 0.0 | 0.0 | 0.0 | 0.0 | 0.0 | 2.1052631578947367 | 0.0 | 0.0 | 2.1052631578947367 | 1.0526315789473684 |
| Control | 0.0 | 6.896551724137931 | 41.37931034482759 | 0.0 | 0.0 | 17.24137931034483 | 0.0 | 0.0 | 0.0 | 0.0 | 0.0 | 0.0 | 0.0 | 0.0 | 0.0 | 0.0 | 0.0 | 0.0 | 0.0 | 3.4482758620689653 | 0.0 | 0.0 | 0.0 | 0.0 | 0.0 | 0.0 | 0.0 | 0.0 | 0.0 | 0.0 | 0.0 | 0.0 | 17.24137931034483 | 0.0 | 3.4482758620689653 | 0.0 | 0.0 | 6.896551724137931 | 0.0 | 0.0 | 0.0 | 0.0 | 0.0 | 0.0 | 3.4482758620689653 | 0.0 | 0.0 | 0.0 |
| 10^4 CFU/g | 0.0 | 0.0 | 42.42424242424242 | 0.0 | 0.0 | 0.0 | 6.0606060606060606 | 0.0 | 0.0 | 0.0 | 0.0 | 0.0 | 0.0 | 0.0 | 0.0 | 0.0 | 0.0 | 0.0 | 12.121212121212121 | 0.0 | 0.0 | 0.0 | 0.0 | 0.0 | 0.0 | 0.0 | 0.0 | 0.0 | 0.0 | 30.303030303030305 | 0.0 | 0.0 | 0.0 | 0.0 | 0.0 | 0.0 | 0.0 | 0.0 | 9.090909090909092 | 0.0 | 0.0 | 0.0 | 0.0 | 0.0 | 0.0 | 0.0 | 0.0 | 0.0 |
D
### Chart: n° of clusters (strains) per type of interaction
| Category | Achromobacter | Tumebacillus | Bacillus | Bosea | Tardiphaga | Burkholderia | Paraburkholderia | Ralstonia | Brevundimonas | Caulobacter | Cellulomonas | Variovorax | Corynebacterium | Enterobacter | Lelliottia | Pantoea | Chryseobacterium | Methylobacterium | Leifsonia | Lysinimonas | Microbacterium | Rathayibacter | Micromonospora | Mycolicibacterium | Rhodococcus | Nocardia | Massilia | Brevibacillus | Cohnella | Paenibacillus | Cellulosimicrobium | Pseudomonas | Agrobacterium | Neorhizobium | Rhizobium | Shinella | Dyella | Luteibacter | Rhodanobacter | Inquilinus | Mucilaginibacter | Novosphingobium | Sphingobium | Sphingomonas | Staphylococcus | Streptomyces | Microbispora | Stenotrophomonas |
|---|---|---|---|---|---|---|---|---|---|---|---|---|---|---|---|---|---|---|---|---|---|---|---|---|---|---|---|---|---|---|---|---|---|---|---|---|---|---|---|---|---|---|---|---|---|---|---|---|
| Complete Inhibition | 0.0 | 0.0 | 21.0 | 0.0 | 0.0 | 8.0 | 0.0 | 0.0 | 0.0 | 0.0 | 0.0 | 0.0 | 0.0 | 14.0 | 1.0 | 1.0 | 0.0 | 0.0 | 5.0 | 0.0 | 0.0 | 1.0 | 0.0 | 0.0 | 0.0 | 0.0 | 0.0 | 0.0 | 0.0 | 2.0 | 0.0 | 5.0 | 0.0 | 0.0 | 0.0 | 0.0 | 0.0 | 0.0 | 0.0 | 0.0 | 0.0 | 0.0 | 0.0 | 0.0 | 1.0 | 0.0 | 0.0 | 0.0 |
| Negative | 0.0 | 0.0 | 20.0 | 0.0 | 0.0 | 1.0 | 1.0 | 1.0 | 1.0 | 0.0 | 0.0 | 0.0 | 0.0 | 19.0 | 0.0 | 0.0 | 3.0 | 0.0 | 9.0 | 2.0 | 0.0 | 4.0 | 0.0 | 0.0 | 0.0 | 0.0 | 0.0 | 1.0 | 0.0 | 5.0 | 0.0 | 4.0 | 3.0 | 0.0 | 0.0 | 0.0 | 2.0 | 0.0 | 0.0 | 0.0 | 1.0 | 0.0 | 0.0 | 0.0 | 0.0 | 0.0 | 0.0 | 0.0 |
| Neutral | 0.0 | 2.0 | 7.0 | 0.0 | 0.0 | 0.0 | 0.0 | 0.0 | 2.0 | 2.0 | 1.0 | 0.0 | 1.0 | 0.0 | 0.0 | 0.0 | 3.0 | 0.0 | 2.0 | 1.0 | 1.0 | 0.0 | 0.0 | 2.0 | 1.0 | 0.0 | 1.0 | 0.0 | 4.0 | 23.0 | 1.0 | 1.0 | 6.0 | 0.0 | 5.0 | 2.0 | 0.0 | 1.0 | 0.0 | 1.0 | 0.0 | 1.0 | 1.0 | 5.0 | 0.0 | 2.0 | 2.0 | 0.0 |
| Positive | 4.0 | 0.0 | 1.0 | 1.0 | 1.0 | 0.0 | 1.0 | 0.0 | 0.0 | 0.0 | 0.0 | 1.0 | 1.0 | 0.0 | 0.0 | 0.0 | 0.0 | 2.0 | 0.0 | 0.0 | 1.0 | 0.0 | 2.0 | 17.0 | 1.0 | 1.0 | 0.0 | 0.0 | 2.0 | 6.0 | 1.0 | 0.0 | 6.0 | 1.0 | 2.0 | 1.0 | 0.0 | 4.0 | 3.0 | 0.0 | 1.0 | 0.0 | 1.0 | 5.0 | 0.0 | 0.0 | 0.0 | 8.0 |Suppl. figure 2. Abundance of bacterial isolates and strains, and the type of interaction with S. indica. Number of bacterial clusters (strains) and their phylogeny per type of interaction. A: total family abundance of isolates and clusters (strains) recovered from roots. B: clusters (strains) isolated from potato and tomato plants. C: relative abundance of strains at the genus level isolated from potato and tomato roots, and from plants pre-inoculated or not with S. indica. D: number of bacterial clusters (strains) and their phylogeny at the genus level per type of interaction.

## Slide 3
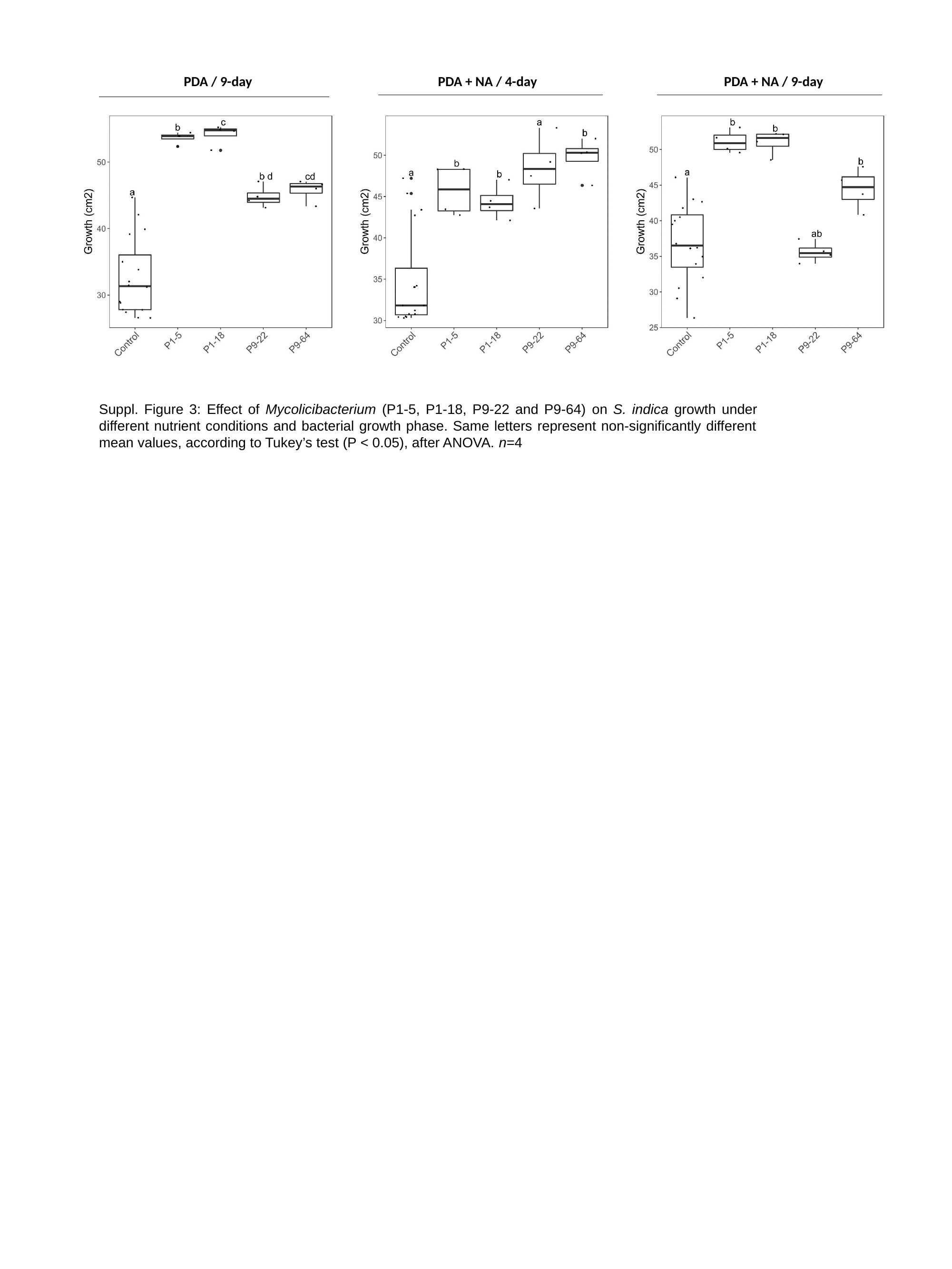

PDA / 9-day
PDA + NA / 4-day
PDA + NA / 9-day
Suppl. Figure 3: Effect of Mycolicibacterium (P1-5, P1-18, P9-22 and P9-64) on S. indica growth under different nutrient conditions and bacterial growth phase. Same letters represent non-significantly different mean values, according to Tukey’s test (P < 0.05), after ANOVA. n=4

## Slide 4
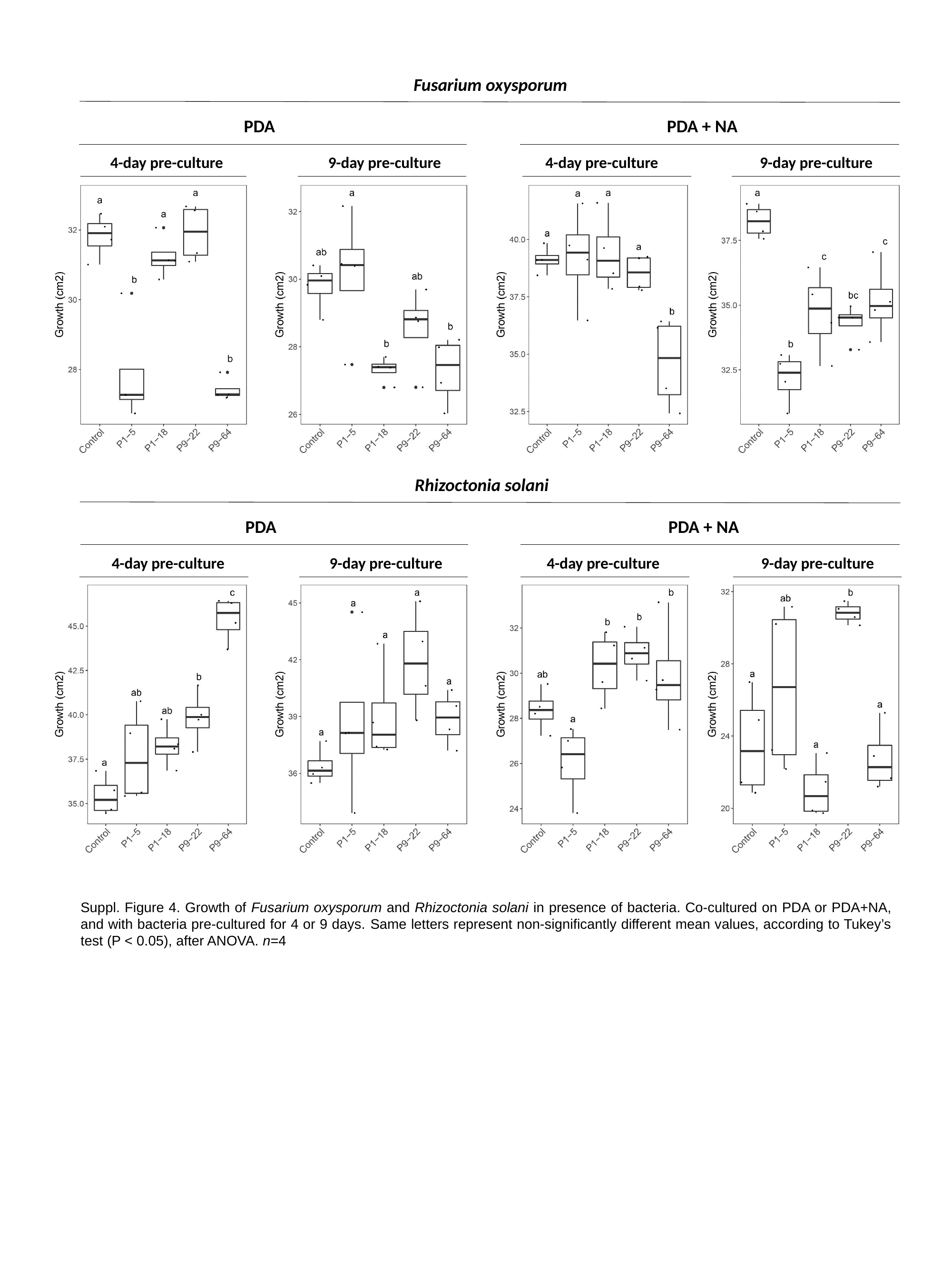

Fusarium oxysporum
PDA
PDA + NA
4-day pre-culture
9-day pre-culture
4-day pre-culture
9-day pre-culture
Rhizoctonia solani
PDA
PDA + NA
4-day pre-culture
9-day pre-culture
4-day pre-culture
9-day pre-culture
Suppl. Figure 4. Growth of Fusarium oxysporum and Rhizoctonia solani in presence of bacteria. Co-cultured on PDA or PDA+NA, and with bacteria pre-cultured for 4 or 9 days. Same letters represent non-significantly different mean values, according to Tukey’s test (P < 0.05), after ANOVA. n=4
