# Supplementary material for: Beneficial Endophytic Bacteria-Serendipita indica Interaction for Crop Enhancement and Resistance to Phytopathogens
Source: Front Microbiol. 2019 Dec 19;10:2888. doi: 10.3389/fmicb.2019.02888 (PMC6930893; doi:10.3389/fmicb.2019.02888)
Supplement: Supplementary file 3 [file Data_Sheet_2.docx]

Suppl. Table 2: Species relatedness of *Mycolicibacterium* isolates P1-5, P1-18, P9-22 and P9-64 to the closest genomes available in the NCBI database according to ANIb and TETRA values.

|  | ANI | |  | TETRA | |
| --- | --- | --- | --- | --- | --- |
| Isolate | Closest genomes | % |  | Closest genomes | % |
| P1-5 | *M.* sp. EPa45 | 0.8847 |  | *M.* sp. EPa45 | 0.9989 |
|  | *M.* sp. WY10 | 0.8751 |  | *M.* sp. WY10 | 0.9977 |
|  | *M.* sp. PYR15 | 0.8739 |  | *M. rhodesiae* DSM 44223 | 0.9976 |
| P1-18 | *M. hodleri* JCM 12141 | 0.8932 |  | *M. chubuense* NBB4 | 0.9820 |
|  | *M.* sp. P9-64 | 0.7878 |  | *M. gilvum* PYR-GCK | 0.9812 |
|  | *M. phlei* CCUG 21000 | 0.7811 |  | *M. gilvum* NCTC10742 | 0.9806 |
| P9-22 | *M.* sp. YC-RL4 | 0.8607 |  | *M.* sp. YC-RL4 | 0.9972 |
|  | *M. diernhoferi* BM1 | 0.8318 |  | *M. bacteremicum* DSM 45578 | 0.9924 |
|  | *M. diernhoferi* IP141170001 | 0.8314 |  | *M. diernhoferi* BM1 | 0.9910 |
| P9-64 | *M. hodleri* JCM 12141 | 0.7996 |  | *M.* sp. CECT 8783 | 0.9849 |
|  | *M.* sp. P9-22 | 0.7880 |  | *M. moriokaense* GAS496 | 0.9828 |
|  | *M. phlei* DSM 43072 | 0.7770 |  | *M.* sp. JS623 | 0.9789 |

Suppl. Table 3: Protein encoding genes predicted to be involved in fungal growth stimulation of strains P1-5, P1-18, P9-22 and P9-64 determined by RAST.

| **Myco. P1-5 (fig\|6666666.353102)** | | |  |  |
| --- | --- | --- | --- | --- |
| Category | Subsytem | Role/ Protein | EC number | Protein encoding genes |
| Vitamins/ Cofactors | Biotin (vitamin B7) biosynthesis | Competence protein F homolog, phosphoribosyltransferase domain |  | *.peg.2724 |
|  |  | Adenosylmethionine-8-amino-7-oxononanoate aminotransferase | 2.6.1.62 | *.peg.77, *.peg.1648 |
|  |  | 8-amino-7-oxononanoate synthase | 2.3.1.47 | *.peg.1647, *.peg.3614 |
|  |  | Dethiobiotin synthetase | 6.3.3.3 | *.peg.1646 |
|  |  | Biotin synthase | 2.8.1.6 | *.peg.1643 |
|  |  | Long-chain-fatty-acid--CoA ligase | 6.2.1.3 | *.peg.380, *.peg.508, *.peg.557, *.peg.564, *.peg.634, *.peg.729, *.peg.754, *.peg.757, *.peg.1181, *.peg.1966, *.peg.2074, *.peg.2523, *.peg.3128, *.peg.3145, *.peg.3165, *.peg.3546, *.peg.3615, *.peg.3658, *.peg.4269, *.peg.4622, *.peg.4634, *.peg.4635, *.peg.4980, *.peg.5140 |
|  |  | 3-ketoacyl-CoA thiolase | 2.3.1.16 | *.peg.378, *.peg.470, *.peg.714, *.peg.1503, *.peg.1841, *.peg.2560, *.peg.2631, *.peg.3175, *.peg.3921, *.peg.3994, *.peg.4009, *.peg.4628 |
|  |  | tRNA (cytidine(34)-2'-O)-methyltransferase | 2.1.1.207 | *.peg.3466 |
|  |  | ATPase component BioM of energizing module of biotin ECF transporter |  | *.peg.815 |
|  |  | Predicted biotin repressor from TetR family |  | *.peg.1644 |
|  |  | predicted biotin regulatory protein BioR (GntR family) |  | *.peg.3130 |
|  | Thiamin (vitamin B1) biosynthesis | Sulfur carrier protein adenylyltransferase ThiF |  | *.peg.2759 |
|  |  | Glycine oxidase ThiO | 1.4.3.19 | *.peg.3854 |
|  |  | Thiazole biosynthesis protein ThiG |  | *.peg.3852 |
|  |  | Thiamine-monophosphate kinase | 2.7.4.16 | *.peg.2375 |
|  |  | Thiamin-phosphate pyrophosphorylase | 2.5.1.3 | *.peg.3855 |
|  |  | Sulfur carrier protein ThiS |  | *.peg.3853 |
|  |  | 1-deoxy-D-xylulose 5-phosphate synthase | 2.2.1.7 | *.peg.1999 |
|  |  | Naphthoate synthase | 4.1.3.36 | *.peg.635, *.peg.751, *.peg.2793, *.peg.3664 |
|  | Mena- quinone and Phyllo- quinone (vitamin K1 & K2) biosynthesis | Naphthoate synthase | 4.1.3.36 | *.peg.635, *.peg.751, *.peg.2793, *.peg.3664 |
|  |  | 2-succinyl-6-hydroxy-2,4-cyclohexadiene-1-carboxylate synthase | 4.2.99.20 | *.peg.3651 |
|  |  | 2-succinyl-5-enolpyruvyl-6-hydroxy-3-cyclohexene-1-carboxylic-acid synthase | 2.2.1.9 | *.peg.3650 |
|  |  | 2-heptaprenyl-1,4-naphthoquinone methyltransferase | 2.1.1.163 | *.peg.3644 |
|  |  | O-succinylbenzoic acid--CoA ligase | 6.2.1.26 | *.peg.3660, *.peg.3671, *.peg.5142 |
|  |  | O-succinylbenzoate synthase | 4.2.1.113 | *.peg.85, *.peg.3657 |
|  |  | 1,4-dihydroxy-2-naphthoate polyprenyltransferase | 2.5.1.74 | *.peg.3689 |
|  | Cobalamin (vitamin B12) synthesis | Cobalt-precorrin-6x reductase | 1.3.1.54 | *.peg.1576 |
|  |  | Cobalamin biosynthesis protein BluB |  | *.peg.4965 |
|  |  | Adenosylcobinamide-phosphate guanylyltransferase | 2.7.7.62 | *.peg.4585 |
|  |  | Cobalt-precorrin-8x methylmutase | 5.4.1.2 | *.peg.1573 |
|  |  | Cobalt-precorrin-2 C20-methyltransferase | 2.1.1.130 | *.peg.1574 |
|  |  | Cobyric acid synthase | 6.3.5.10 | *.peg.2174 |
|  |  | Cobalt-precorrin-4 C11-methyltransferase | 2.1.1.133 | *.peg.1577 |
|  |  | Cobalt-precorrin-3b C17-methyltransferase |  | *.peg.1574 |
|  |  | Nicotinate-nucleotide--dimethylbenzimidazole phosphoribosyltransferase | 2.4.2.21 | *.peg.4586 |
|  |  | Adenosylcobinamide-phosphate synthase | 6.3.1.10 | *.peg.349 |
|  |  | Cob(I)alamin adenosyltransferase | 2.5.1.17 | *.peg.2132 |
|  |  | Cobyrinic acid A,C-diamide synthase |  | *.peg.2131 |
|  | Heme and Siroheme biosynthesis | Hypothetical radical SAM family enzyme in heat shock gene cluster, similarity with CPO of BS HemN-type |  | *.peg.208 |
|  |  | Glutamyl-tRNA synthetase | 6.1.1.17 | *.peg.2389 |
|  |  | Uroporphyrinogen-III synthase | 4.2.1.75 | *.peg.1656, *.peg.3717 |
|  |  | Glutamate-1-semialdehyde aminotransferase | 5.4.3.8 | *.peg.3698 |
|  |  | Precorrin-2 oxidase | 1.3.1.76 | *.peg.2130 |
|  |  | Porphobilinogen synthase | 4.2.1.24 | *.peg.3716 |
|  |  | Hemoprotein HemQ, essential component of heme biosynthetic pathway in Gram-positive bacteria |  | *.peg.1994 |
|  |  | Glutamyl-tRNA reductase | 1.2.1.70 | *.peg.3719 |
|  |  | Porphobilinogen deaminase | 2.5.1.61 | *.peg.3718 |
|  |  | Uroporphyrinogen-III methyltransferase | 2.1.1.107 | *.peg.2130, *.peg.3717 |
|  |  | Ferrochelatase, protoheme ferro-lyase | 4.99.1.1 | *.peg.1705 |
|  |  | Uroporphyrinogen III decarboxylase | 4.1.1.37 | *.peg.1996 |
|  |  | Protoporphyrinogen IX oxidase, aerobic, HemY | 1.3.3.4 | *.peg.1995 |
|  |  | Sirohydrochlorin ferrochelatase | 4.99.1.4 | *.peg.205, *.peg.1655, *.peg.2130 |
|  | Riboflavin (vitamin B2) metabolism | FMN adenylyltransferase | 2.7.7.2 | *.peg.2101 |
|  |  | FIG000859: hypothetical protein YebC |  | *.peg.1899 |
|  |  | Molybdopterin binding motif, CinA N-terminal domain |  | *.peg.133 |
|  |  | C-terminal domain of CinA type S |  | *.peg.133, *.peg.2062 |
|  |  | 6,7-dimethyl-8-ribityllumazine synthase | 2.5.1.78 | *.peg.1756 |
|  |  | 5-amino-6-(5-phosphoribosylamino)uracil reductase | 1.1.1.193 | *.peg.1767 |
|  |  | tRNA pseudouridine synthase B | 4.2.1.70 | *.peg.2105 |
|  |  | Riboflavin kinase | 2.7.1.26 | *.peg.2101 |
|  |  | GTP cyclohydrolase II | 3.5.4.25 | *.peg.1525, *.peg.1757 |
|  |  | Riboflavin synthase eubacterial/eukaryotic | 2.5.1.9 | *.peg.1761 |
|  |  | Diaminohydroxyphosphoribosylaminopyrimidine deaminase | 3.5.4.26 | *.peg.1767 |
|  | Pyridoxin (vitamin B6) biosynthesis | Pyridoxine biosynthesis glutamine amidotransferase, synthase subunit | 2.4.2.- | *.peg.1918 |
|  |  | Pyridoxine biosynthesis glutamine amidotransferase, glutaminase subunit | 2.4.2.- | *.peg.1916 |
|  |  | D-3-phosphoglycerate dehydrogenase | 1.1.1.95 | *.peg.2393, *.peg.3250, *.peg.3931, *.peg.4844 |
|  |  | Pyridoxamine 5'-phosphate oxidase | 1.4.3.5 | *.peg.416 |
|  |  | Phosphoserine aminotransferase | 2.6.1.52 | *.peg.408 |
|  |  | NAD-dependent glyceraldehyde-3-phosphate dehydrogenase | 1.2.1.12 | *.peg.1748 |
|  |  | 1-deoxy-D-xylulose 5-phosphate synthase | 2.2.1.7 | *.peg.1999 |
|  | Folate (vitamin B9) biosynthesis | Ketopantoate reductase PanG | 1.1.1.169 | *.peg.1449 |
|  |  | Dihydropteroate synthase | 2.5.1.15 | *.peg.1444 |
|  |  | tRNA(Ile)-lysidine synthetase | 6.3.4.19 | *.peg.1435 |
|  |  | Hypoxanthine-guanine phosphoribosyltransferase | 2.4.2.8 | *.peg.1436 |
|  |  | FIG027937: secreted protein |  | *.peg.52, *.peg.1447, *.peg.3983 |
|  |  | Aspartate 1-decarboxylase | 4.1.1.11 | *.peg.1451 |
|  |  | membrane-flanked domain |  | *.peg.1351 |
|  |  | Cell division protein FtsH | 3.4.24.- | *.peg.1442, *.peg.3813, *.peg.4376 |
|  |  | GTP cyclohydrolase I | 3.5.4.16 type 1 | *.peg.1443 |
|  |  | Pantoate--beta-alanine ligase | 6.3.2.1 | *.peg.1450 |
|  |  | 2-amino-4-hydroxy-6-hydroxymethyldihydropteridine pyrophosphokinase | 2.7.6.3 | *.peg.1446 |
|  |  | Dihydroneopterin aldolase | 4.1.2.25 | *.peg.1445 |
|  |  | Aminodeoxychorismate lyase | 4.1.3.38 | *.peg.4676 |
|  |  | Folylpolyglutamate synthase | 6.3.2.17 | *.peg.2356 |
|  |  | 5-formyltetrahydrofolate cyclo-ligase | 6.3.3.2 | *.peg.1129 |
|  |  | Dihydrofolate reductase | 1.5.1.3 | *.peg.2076, *.peg.2326, *.peg.3939 |
|  |  | Thymidylate synthase thyX | 2.1.1.- | *.peg.2073 |
|  |  | Dihydrofolate synthase | 6.3.2.12 | *.peg.2356 |
|  |  | Para-aminobenzoate synthase, aminase component | 2.6.1.85 | *.peg.1171, *.peg.3607 |
|  |  | Thymidylate synthase | 2.1.1.45 | *.peg.2077 |
|  |  | Para-aminobenzoate synthase, amidotransferase component | 2.6.1.85 | *.peg.3607, *.peg.4355 |
| Cell Wall/ Secretion | Amino acid and peptide ABC transporter | Oligopeptide transport system permease protein OppC | 3.A.1.5.1 | *.peg.676 |
|  |  | Oligopeptide transport system permease protein OppB | 3.A.1.5.1 | *.peg.675, *.peg.677 |
|  |  | Oligopeptide ABC transporter, periplasmic oligopeptide-binding protein OppA | 3.A.1.5.1 | *.peg.674, *.peg.1880 |
|  |  | High-affinity branched-chain amino acid transport system permease protein LivH | 3.A.1.4.1 | *.peg.4460 |
|  |  | Branched-chain amino acid transport ATP-binding protein LivF | 3.A.1.4.1 | *.peg.4457 |
|  |  | Branched-chain amino acid transport system permease protein LivM | 3.A.1.4.1 | *.peg.4459 |
|  |  | Branched-chain amino acid ABC transporter, amino acid-binding protein | 3.A.1.4.1 | *.peg.4461 |
|  |  | Branched-chain amino acid transport ATP-binding protein LivG | 3.A.1.4.1 | *.peg.4458 |
|  |  | Dipeptide transport system permease protein DppB | 3.A.1.5.2 | *.peg.286 |
|  |  | Dipeptide transport ATP-binding protein DppD | 3.A.1.5.2 | *.peg.284 |
|  |  | Dipeptide transport system permease protein DppC | 3.A.1.5.2 | *.peg.285 |
|  |  | Dipeptide-binding ABC transporter, periplasmic substrate-binding component | 3.A.1.5.2 | *.peg.287 |
| Nitrogen | Nitrate reductase | Respiratory nitrate reductase alpha chain | 1.7.99.4 | *.peg.1675 |
|  |  | Assimilatory nitrate reductase large subunit | 1.7.99.4 | *.peg.1660, *.peg.2367 |
|  |  | Respiratory nitrate reductase gamma chain | 1.7.99.4 | *.peg.1672 |
|  |  | Respiratory nitrate reductase beta chain | 1.7.99.4 | *.peg.1674 |
|  |  | Respiratory nitrate reductase delta chain | 1.7.99.4 | *.peg.1673 |
|  | Nitrite reductase | Nitrite reductase [NAD(P)H] large subunit | 1.7.1.4 | *.peg.1658 |
|  |  | Nitrite reductase [NAD(P)H] small subunit | 1.7.1.4 | *.peg.1657 |
|  | Nitrate/ite transporter | Nitrate/nitrite transporter |  | *.peg.1659, *.peg.1671, *.peg.3249 |
|  | Nitric oxide reductase | Nitric oxide reductase activation protein NorD |  | *.peg.1608, *.peg.2406 |
|  |  | Nitric oxide reductase activation protein NorQ |  | *.peg.1609, *.peg.2405 |
|  | Ammonium transporter | Ammonium transporter |  | *.peg.2237, *.peg.4759 |
|  | Glutamine synthetase | Glutamine synthetase type I | 6.3.1.2 | *.peg.268, *.peg.3423, *.peg.3429 |
|  |  | Glutamine synthetase family protein in hypothetical Actinobacterial gene cluster |  | *.peg.2168 |
|  | Glutamate synthase | Glutamate synthase [NADPH] large chain | 1.4.1.13 | *.peg.1078, *.peg.4482 |
|  |  | Glutamate synthase [NADPH] small chain | 1.4.1.13 | *.peg.1077, *.peg.4481 |
|  |  | Ferredoxin-dependent glutamate synthase | 1.4.7.1 | *.peg.4410 |
| Carbo- hydrate | Trehalose biosynthesis | Malto-oligosyltrehalose synthase | 5.4.99.15 | *.peg.1651 |
|  |  | 1,4-alpha-glucan (glycogen) branching enzyme, GH-13-type | 2.4.1.18 | *.peg.2563 |
|  |  | Trehalose phosphorylase | 2.4.1.64 | *.peg.2883 |
|  |  | Trehalose-6-phosphate phosphatase | 3.1.3.12 | *.peg.764, *.peg.1484 |
|  |  | Putative glucanase glgE | 3.2.1.- | *.peg.2564 |
|  |  | Malto-oligosyltrehalose trehalohydrolase | 3.2.1.141 | *.peg.1652 |
|  |  | Glycogen debranching enzyme | 3.2.1.- | *.peg.1650, *.peg.3003 |
|  |  | Alpha,alpha-trehalose-phosphate synthase [UDP-forming] | 2.4.1.15 | *.peg.4771 |
|  |  | Glucoamylase | 3.2.1.3 | *.peg.199, *.peg.2163 |
| **Myco. P1-18 (fig\|6666666.353104)** | | |  |  |
| Category | Subsytem | Role/ Protein | EC number | Protein encoding genes |
| Vitamins/ Cofactors | Biotin (vitamin B7) biosynthesis | Competence protein F homolog, phosphoribosyltransferase domain |  | *.peg.4807 |
|  |  | Substrate-specific component BioY of biotin ECF transporter |  | *.peg.840 |
|  |  | Adenosylmethionine-8-amino-7-oxononanoate aminotransferase | 2.6.1.62 | *.peg.2435, *.peg.3183 |
|  |  | 8-amino-7-oxononanoate synthase | 2.3.1.47 | *.peg.3184 |
|  |  | Dethiobiotin synthetase | 6.3.3.3 | *.peg.3185 |
|  |  | Biotin synthase | 2.8.1.6 | *.peg.3188 |
|  |  | Long-chain-fatty-acid--CoA ligase | 6.2.1.3 | *.peg.370, *.peg.693, *.peg.746, *.peg.929, *.peg.1233, *.peg.1373, *.peg.2214, *.peg.2257, *.peg.2385, *.peg.2396, *.peg.2604, *.peg.4266, *.peg.6239, *.peg.6250 |
|  |  | 3-ketoacyl-CoA thiolase | 2.3.1.16 | *.peg.789, *.peg.821, *.peg.2203, *.peg.2997, *.peg.4996, *.peg.5267, *.peg.5435, *.peg.5514, *.peg.6326, *.peg.6408, *.peg.6423 |
|  |  | tRNA (cytidine(34)-2'-O)-methyltransferase | 2.1.1.207 | *.peg.4021 |
|  |  | ATPase component BioM of energizing module of biotin ECF transporter |  | *.peg.761 |
|  |  | Predicted biotin repressor from TetR family |  | *.peg.3187 |
|  |  | predicted biotin regulatory protein BioR (GntR family) |  | *.peg.2256 |
|  | Thiamin (vitamin B1) biosynthesis | Sulfur carrier protein adenylyltransferase ThiF |  | *.peg.4772 |
|  |  | Predicted hydroxymethylpyrimidine transporter CytX |  | *.peg.3950 |
|  |  | Glycine oxidase ThiO | 1.4.3.19 | *.peg.1952 |
|  |  | Thiazole biosynthesis protein ThiG |  | *.peg.1950 |
|  |  | Transmembrane component YkoC of energizing module of thiamin-regulated ECF transporter for HydroxyMethylPyrimidine |  | *.peg.5227 |
|  |  | Thiamine-monophosphate kinase | 2.7.4.16 | *.peg.2555 |
|  |  | 1-deoxy-D-xylulose 5-phosphate synthase | 2.2.1.7 | *.peg.2812 |
|  |  | Substrate-specific component YkoE of thiamin-regulated ECF transporter for HydroxyMethylPyrimidine |  | *.peg.5225 |
|  |  | Thiaminase II | 3.5.99.2 | *.peg.5784 |
|  |  | Thiamin-phosphate pyrophosphorylase | 2.5.1.3 | *.peg.1953 |
|  |  | Thiamin ABC transporter, transmembrane component |  | *.peg.3772 |
|  |  | Sulfur carrier protein ThiS |  | *.peg.1951 |
|  | Mena- quinone and Phyllo- quinone (vitamin K1 & K2) biosynthesis | Naphthoate synthase | 4.1.3.36 | *.peg.1707 |
|  |  | 2-succinyl-6-hydroxy-2,4-cyclohexadiene-1-carboxylate synthase | 4.2.99.20 | *.peg.1692 |
|  |  | 2-succinyl-5-enolpyruvyl-6-hydroxy-3-cyclohexene-1-carboxylic-acid synthase | 2.2.1.9 | *.peg.1691 |
|  |  | O-succinylbenzoic acid--CoA ligase | 6.2.1.26 | *.peg.1698, *.peg.1702, *.peg.1718, *.peg.6171 |
|  |  | O-succinylbenzoate synthase | 4.2.1.113 | *.peg.1694, *.peg.5773 |
|  |  | 1,4-dihydroxy-2-naphthoate polyprenyltransferase | 2.5.1.74 | *.peg.1775 |
|  | Cobalamin (vitamin B12) synthesis | Cobalt-precorrin-6x reductase | 1.3.1.54 | *.peg.1064 |
|  |  | Cobalamin biosynthesis protein BluB |  | *.peg.5028 |
|  |  | Adenosylcobinamide-phosphate guanylyltransferase | 2.7.7.62 | *.peg.1302 |
|  |  | Cobalt-precorrin-8x methylmutase | 5.4.1.2 | *.peg.1062 |
|  |  | Cobalt-precorrin-2 C20-methyltransferase | 2.1.1.130 | *.peg.4105 |
|  |  | Cobyric acid synthase | 6.3.5.10 | *.peg.2655 |
|  |  | Cobalt-precorrin-4 C11-methyltransferase | 2.1.1.133 | *.peg.1065 |
|  |  | Cobalt-precorrin-3b C17-methyltransferase |  | *.peg.4105 |
|  |  | Nicotinate-nucleotide--dimethylbenzimidazole phosphoribosyltransferase | 2.4.2.21 | *.peg.4104 |
|  |  | Adenosylcobinamide-phosphate synthase | 6.3.1.10 | *.peg.1259 |
|  |  | Cob(I)alamin adenosyltransferase | 2.5.1.17 | *.peg.2676 |
|  |  | Cobalamin synthase | 2.7.8.26 | *.peg.1301 |
|  |  | Cobyrinic acid A,C-diamide synthase |  | *.peg.2677 |
|  |  | CblZ, a non-orthologous displasment for Alpha-ribazole-5'-phosphate phosphatase |  | *.peg.1303 |
|  |  | Cobalt-precorrin-6y C5-methyltransferase | 2.1.1.- | *.peg.1066 |
|  |  | 5,6-dimethylbenzimidazole synthase, flavin destructase family |  | *.peg.5028 |
|  |  | Cobalt-precorrin-6y C15-methyltransferase [decarboxylating] | 2.1.1.- | *.peg.1066 |
|  |  | Uroporphyrinogen-III methyltransferase | 2.1.1.107 | *.peg.1797, *.peg.2678, *.peg.4114 |
|  |  | ChlI component of cobalt chelatase involved in B12 biosynthesis |  | *.peg.2675 |
|  |  | Precorrin-6A synthase | 2.1.1.152 | *.peg.5584 |
|  |  | ChlD component of cobalt chelatase involved in B12 biosynthesis |  | *.peg.2675 |
|  |  | Cobalamin biosynthesis protein CobG |  | *.peg.1061 |
|  |  | Predicted cobalt transporter CbtA |  | *.peg.4707, *.peg.5050 |
|  |  | CobN component of cobalt chelatase involved in B12 biosynthesis |  | *.peg.1058 |
|  | Heme and Siroheme biosynthesis | Hypothetical radical SAM family enzyme in heat shock gene cluster, similarity with CPO of BS HemN-type |  | *.peg.101 |
|  |  | Glutamyl-tRNA synthetase | 6.1.1.17 | *.peg.2542 |
|  |  | Uroporphyrinogen-III synthase | 4.2.1.75 | *.peg.1797, *.peg.2177 |
|  |  | Glutamate-1-semialdehyde aminotransferase | 5.4.3.8 | *.peg.40, *.peg.1786, *.peg.1812, *.peg.1820, *.peg.1940, *.peg.3387, *.peg.3517, *.peg.4502 |
|  |  | Precorrin-2 oxidase | 1.3.1.76 | *.peg.2678, *.peg.4114 |
|  |  | Porphobilinogen synthase | 4.2.1.24 | *.peg.1796 |
|  |  | Hemoprotein HemQ, essential component of heme biosynthetic pathway in Gram-positive bacteria |  | *.peg.2819 |
|  |  | Glutamyl-tRNA reductase | 1.2.1.70 | *.peg.1799 |
|  |  | Porphobilinogen deaminase | 2.5.1.61 | *.peg.1798 |
|  |  | Uroporphyrinogen-III methyltransferase | 2.1.1.107 | *.peg.1797, *.peg.2678, *.peg.4114 |
|  |  | Ferrochelatase, protoheme ferro-lyase | 4.99.1.1 | *.peg.3138 |
|  |  | Uroporphyrinogen III decarboxylase | 4.1.1.37 | *.peg.2817 |
|  |  | Protoporphyrinogen IX oxidase, aerobic, HemY | 1.3.3.4 | *.peg.1695, *.peg.2818 |
|  |  | Sirohydrochlorin ferrochelatase | 4.99.1.4 | *.peg.97, *.peg.2178, *.peg.2678, *.peg.4114 |
|  | Riboflavin (vitamin B2) metabolism | FMN adenylyltransferase | 2.7.7.2 | *.peg.2705 |
|  |  | FIG000859: hypothetical protein YebC |  | *.peg.2949 |
|  |  | Molybdopterin binding motif, CinA N-terminal domain |  | *.peg.5744 |
|  |  | C-terminal domain of CinA type S |  | *.peg.5744 |
|  |  | 6,7-dimethyl-8-ribityllumazine synthase | 2.5.1.78 | *.peg.3076 |
|  |  | 5-amino-6-(5-phosphoribosylamino)uracil reductase | 1.1.1.193 | *.peg.3068 |
|  |  | tRNA pseudouridine synthase B | 4.2.1.70 | *.peg.2703 |
|  |  | Riboflavin kinase | 2.7.1.26 | *.peg.2705 |
|  |  | GTP cyclohydrolase II | 3.5.4.25 | *.peg.798, *.peg.3075 |
|  |  | Riboflavin synthase eubacterial/eukaryotic | 2.5.1.9 | *.peg.3073 |
|  |  | Diaminohydroxyphosphoribosylaminopyrimidine deaminase | 3.5.4.26 | *.peg.3068 |
|  |  | 3,4-dihydroxy-2-butanone 4-phosphate synthase | 4.1.99.12 | *.peg.798, *.peg.3075 |
|  | Pyridoxin (vitamin B6) biosynthesis | Pyridoxine biosynthesis glutamine amidotransferase, synthase subunit | 2.4.2.- | *.peg.2946, *.peg.3473 |
|  |  | Pyridoxine biosynthesis glutamine amidotransferase, glutaminase subunit | 2.4.2.- | *.peg.2948 |
|  |  | D-3-phosphoglycerate dehydrogenase | 1.1.1.95 | *.peg.970, *.peg.1454, *.peg.1556, *.peg.2103, *.peg.2533, *.peg.5087, *.peg.5096 |
|  |  | Pyridoxamine 5'-phosphate oxidase | 1.4.3.5 | *.peg.1788, *.peg.5473, *.peg.6131 |
|  |  | Phosphoserine aminotransferase | 2.6.1.52 | *.peg.5463 |
|  |  | NAD-dependent glyceraldehyde-3-phosphate dehydrogenase | 1.2.1.12 | *.peg.3088 |
|  |  | 1-deoxy-D-xylulose 5-phosphate synthase | 2.2.1.7 | *.peg.2812 |
|  | Folate (vitamin B9) biosynthesis | Ketopantoate reductase PanG | 1.1.1.169 | *.peg.5065 |
|  |  | Dihydropteroate synthase | 2.5.1.15 | *.peg.5070 |
|  |  | tRNA(Ile)-lysidine synthetase | 6.3.4.19 | *.peg.5077 |
|  |  | Hypoxanthine-guanine phosphoribosyltransferase | 2.4.2.8 | *.peg.5076 |
|  |  | FIG027937: secreted protein |  | *.peg.231, *.peg.5067, *.peg.6394 |
|  |  | Aspartate 1-decarboxylase | 4.1.1.11 | *.peg.2321 |
|  |  | membrane-flanked domain |  | *.peg.4843 |
|  |  | Cell division protein FtsH | 3.4.24.- | *.peg.1890, *.peg.4599, *.peg.5072 |
|  |  | GTP cyclohydrolase I | 3.5.4.16 type 1 | *.peg.5071 |
|  |  | transmembrane protein, distant homology with ydbS |  | *.peg.1885 |
|  |  | Pantoate--beta-alanine ligase | 6.3.2.1 | *.peg.5064 |
|  |  | 2-amino-4-hydroxy-6-hydroxymethyldihydropteridine pyrophosphokinase | 2.7.6.3 | *.peg.5068 |
|  |  | Dihydroneopterin aldolase | 4.1.2.25 | *.peg.5069 |
|  |  | Aminodeoxychorismate lyase | 4.1.3.38 | *.peg.5384 |
|  |  | Folylpolyglutamate synthase | 6.3.2.17 | *.peg.27 |
|  |  | 5-formyltetrahydrofolate cyclo-ligase | 6.3.3.2 | *.peg.5641 |
|  |  | Dihydrofolate reductase | 1.5.1.3 | *.peg.1202, *.peg.2229, *.peg.2726, *.peg.4026, *.peg.4403, *.peg.5980 |
|  |  | Dihydrofolate synthase | 6.3.2.12 | *.peg.27 |
|  |  | Para-aminobenzoate synthase, aminase component | 2.6.1.85 | *.peg.919 |
|  |  | Thymidylate synthase | 2.1.1.45 | *.peg.2725 |
|  |  | Para-aminobenzoate synthase, amidotransferase component | 2.6.1.85 | *.peg.3614 |
| Cell Wall/ Secretion | Lipoprotein releasing | Lipoprotein releasing system transmembrane protein LolC |  | *.peg.737 |
|  | Amino acid and peptide ABC transporter | Oligopeptide transport system permease protein OppC | 3.A.1.5.1 | *.peg.6154 |
|  |  | Oligopeptide transport system permease protein OppB | 3.A.1.5.1 | *.peg.217, *.peg.1442, *.peg.2073, *.peg.5092, *.peg.6153, *.peg.6155 |
|  |  | Oligopeptide transport ATP-binding protein OppF | 3.A.1.5.1 | *.peg.2074, *.peg.5091, *.peg.5519 |
|  |  | Oligopeptide transport ATP-binding protein OppD | 3.A.1.5.1 | *.peg.1579 |
|  |  | Oligopeptide ABC transporter, periplasmic oligopeptide-binding protein OppA | 3.A.1.5.1 | *.peg.2070, *.peg.2966, *.peg.6156 |
|  |  | High-affinity branched-chain amino acid transport system permease protein LivH | 3.A.1.4.1 | *.peg.6061 |
|  |  | Branched-chain amino acid transport ATP-binding protein LivF | 3.A.1.4.1 | *.peg.6058 |
|  |  | Branched-chain amino acid transport system permease protein LivM | 3.A.1.4.1 | *.peg.6060 |
|  |  | Branched-chain amino acid ABC transporter, amino acid-binding protein | 3.A.1.4.1 | *.peg.6062 |
|  |  | Branched-chain amino acid transport ATP-binding protein LivG | 3.A.1.4.1 | *.peg.6059 |
|  |  | Dipeptide transport system permease protein DppB | 3.A.1.5.2 | *.peg.219, *.peg.226, *.peg.1444, *.peg.1577, *.peg.2072, *.peg.5521 |
|  |  | Dipeptide transport ATP-binding protein DppD | 3.A.1.5.2 | *.peg.228 |
|  |  | Dipeptide transport system permease protein DppC | 3.A.1.5.2 | *.peg.218, *.peg.227, *.peg.1443, *.peg.1578, *.peg.2071, *.peg.5093, *.peg.5520 |
|  |  | Dipeptide-binding ABC transporter, periplasmic substrate-binding component | 3.A.1.5.2 | *.peg.220, *.peg.225, *.peg.436, *.peg.1576, *.peg.2465, *.peg.2469 |
| Nitrogen | Nitrate reductase | Assimilatory nitrate reductase large subunit | 1.7.99.4 | *.peg.1121, *.peg.2850 |
|  | Nitrite reductase | Nitrite reductase [NAD(P)H] small subunit | 1.7.1.4 | *.peg.2181 |
|  |  | Nitrite reductase [NAD(P)H] large subunit | 1.7.1.4 | *.peg.2182 |
|  | Nitrate/ite transporter | Nitrate/nitrite transporter |  | *.peg.1122 |
|  |  | ABC-type nitrate/sulfonate/bicarbonate transport system, permease component |  | *.peg.3707 |
|  |  | ABC-type nitrate/sulfonate/bicarbonate transport system, ATPase component |  | *.peg.3709 |
|  | Ammonium transporter | Ammonium transporter |  | *.peg.2577, *.peg.5202 |
|  | Glutamine synthetase | Glutamine synthetase type I | 6.3.1.2 | *.peg.67, *.peg.1279, *.peg.1284, *.peg.3330 |
|  |  | Glutamine synthetase type III, GlnN | 6.3.1.2 | *.peg.680 |
|  |  | Glutamine synthetase family protein in hypothetical Actinobacterial gene cluster |  | *.peg.2661 |
|  | Glutamate synthase | Glutamate synthase [NADPH] large chain | 1.4.1.13 | *.peg.3333, *.peg.3827, *.peg.4419, *.peg.6086 |
|  |  | Glutamate synthase [NADPH] small chain | 1.4.1.13 | *.peg.3828, *.peg.6085 |
|  |  | Glutamate synthase [NADPH] putative GlxC chain | 1.4.1.13 | *.peg.3332 |
| Carbo- hydrates | Trehalose biosynthesis | Malto-oligosyltrehalose synthase | 5.4.99.15 | *.peg.3180 |
|  |  | Putative glucanase glgE | 3.2.1.- | *.peg.817 |
|  |  | 1,4-alpha-glucan (glycogen) branching enzyme, GH-13-type | 2.4.1.18 | *.peg.818 |
|  |  | Trehalose phosphorylase | 2.4.1.64 | *.peg.1740 |
|  |  | Trehalose synthase | 5.4.99.16 | *.peg.1466 |
|  |  | Trehalose-6-phosphate phosphatase | 3.1.3.12 | *.peg.5021 |
|  |  | Alpha,alpha-trehalose-phosphate synthase [UDP-forming] | 2.4.1.15 | *.peg.1621 |
|  |  | Glycogen debranching enzyme | 3.2.1.- | *.peg.3181, *.peg.3786 |
|  |  | Malto-oligosyltrehalose trehalohydrolase | 3.2.1.141 | *.peg.3179 |
|  |  | Glucoamylase | 3.2.1.3 | *.peg.87, *.peg.2033, *.peg.2854 |
| **Myco. P9-22 (fig\|6666666.353106)** | | |  |  |
| Category | Subsytem | Role/ Protein | EC number | Protein encoding genes |
| Vitamins/ Cofactors | Biotin (vitamin B7) biosynthesis | Competence protein F homolog, phosphoribosyltransferase domain |  | *.peg.2722 |
|  |  | Adenosylmethionine-8-amino-7-oxononanoate aminotransferase | 2.6.1.62 | *.peg.995, *.peg.4382 |
|  |  | 8-amino-7-oxononanoate synthase | 2.3.1.47 | *.peg.4381 |
|  |  | Dethiobiotin synthetase | 6.3.3.3 | *.peg.4380 |
|  |  | Biotin synthase | 2.8.1.6 | *.peg.4376 |
|  |  | Long-chain-fatty-acid--CoA ligase | 6.2.1.3 | *.peg.700, *.peg.860, *.peg.878, *.peg.978, *.peg.1118, *.peg.1122, *.peg.1448, *.peg.1556, *.peg.1558, *.peg.1579, *.peg.1674, *.peg.1713, *.peg.2059, *.peg.2099, *.peg.2100, *.peg.2522, *.peg.2837, *.peg.2839, *.peg.2995, *.peg.4065, *.peg.4146, *.peg.4328, *.peg.4959, *.peg.4983, *.peg.4984, *.peg.4985, *.peg.5033, *.peg.6250, *.peg.6262, *.peg.6323 |
|  |  | 3-ketoacyl-CoA thiolase | 2.3.1.16 | *.peg.84, *.peg.103, *.peg.196, *.peg.903, *.peg.1405, *.peg.1496, *.peg.2132, *.peg.3797, *.peg.4572, *.peg.4977, *.peg.4993 |
|  |  | tRNA (cytidine(34)-2'-O)-methyltransferase | 2.1.1.207 | *.peg.2938 |
|  |  | ATPase component BioM of energizing module of biotin ECF transporter |  | *.peg.1589 |
|  |  | Predicted biotin repressor from TetR family |  | *.peg.4377 |
|  |  | predicted biotin regulatory protein BioR (GntR family) |  | *.peg.449 |
|  | Thiamin (vitamin B1) biosynthesis | Sulfur carrier protein adenylyltransferase ThiF |  | *.peg.2668, *.peg.3101 |
|  |  | Hydroxymethylpyrimidine ABC transporter, substrate-binding component |  | *.peg.4806, *.peg.4871, *.peg.6510, *.peg.6522 |
|  |  | Glycine oxidase ThiO | 1.4.3.19 | *.peg.3595 |
|  |  | Thiazole biosynthesis protein ThiG |  | *.peg.3593 |
|  |  | Thiamine-monophosphate kinase | 2.7.4.16 | *.peg.6396 |
|  |  | 1-deoxy-D-xylulose 5-phosphate synthase | 2.2.1.7 | *.peg.4722 |
|  |  | Hydroxymethylpyrimidine ABC transporter, ATPase component |  | *.peg.1693, *.peg.4872 |
|  |  | Hydroxymethylpyrimidine ABC transporter, transmembrane component |  | *.peg.4870 |
|  |  | Thiamin-phosphate pyrophosphorylase | 2.5.1.3 | *.peg.3596 |
|  |  | Thiamin ABC transporter, transmembrane component |  | *.peg.1022 |
|  |  | Sulfur carrier protein ThiS |  | *.peg.3594 |
|  | Mena- quinone and Phyllo- quinone (vitamin K1 & K2) biosynthesis | Naphthoate synthase | 4.1.3.36 | *.peg.3396 |
|  |  | 2-succinyl-6-hydroxy-2,4-cyclohexadiene-1-carboxylate synthase | 4.2.99.20 | *.peg.3371 |
|  |  | 2-succinyl-5-enolpyruvyl-6-hydroxy-3-cyclohexene-1-carboxylic-acid synthase | 2.2.1.9 | *.peg.3370 |
|  |  | 2-heptaprenyl-1,4-naphthoquinone methyltransferase | 2.1.1.163 | *.peg.3364 |
|  |  | O-succinylbenzoic acid--CoA ligase | 6.2.1.26 | *.peg.2123, *.peg.3376, *.peg.3379, *.peg.3421, *.peg.5006 |
|  |  | O-succinylbenzoate synthase | 4.2.1.113 | *.peg.2533, *.peg.3375 |
|  |  | 1,4-dihydroxy-2-naphthoate polyprenyltransferase | 2.5.1.74 | *.peg.3441 |
|  | Cobalamin (vitamin B12) synthesis | Cobalt-precorrin-6x reductase | 1.3.1.54 | *.peg.298 |
|  |  | Cobalamin biosynthesis protein BluB |  | *.peg.3738 |
|  |  | L-threonine 3-O-phosphate decarboxylase | 4.1.1.81 | *.peg.2259 |
|  |  | Adenosylcobinamide-phosphate guanylyltransferase | 2.7.7.62 | *.peg.2298 |
|  |  | Cobalt-precorrin-8x methylmutase | 5.4.1.2 | *.peg.302 |
|  |  | Cobalt-precorrin-2 C20-methyltransferase | 2.1.1.130 | *.peg.301 |
|  |  | Cobyric acid synthase | 6.3.5.10 | *.peg.4903 |
|  |  | Cobalt-precorrin-4 C11-methyltransferase | 2.1.1.133 | *.peg.297 |
|  |  | Cobalt-precorrin-3b C17-methyltransferase |  | *.peg.301 |
|  |  | Nicotinate-nucleotide--dimethylbenzimidazole phosphoribosyltransferase | 2.4.2.21 | *.peg.2297 |
|  |  | Adenosylcobinamide-phosphate synthase | 6.3.1.10 | *.peg.2255 |
|  |  | Cob(I)alamin adenosyltransferase | 2.5.1.17 | *.peg.4889 |
|  |  | Cobyrinic acid A,C-diamide synthase |  | *.peg.4888 |
|  | Heme and Siroheme biosynthesis | Hypothetical radical SAM family enzyme in heat shock gene cluster, similarity with CPO of BS HemN-type |  | *.peg.1927 |
|  |  | Glutamyl-tRNA synthetase | 6.1.1.17 | *.peg.6411 |
|  |  | Uroporphyrinogen-III synthase | 4.2.1.75 | *.peg.932, *.peg.3467 |
|  |  | Glutamate-1-semialdehyde aminotransferase | 5.4.3.8 | *.peg.2444, *.peg.3455 |
|  |  | Precorrin-2 oxidase | 1.3.1.76 | *.peg.4887 |
|  |  | Porphobilinogen synthase | 4.2.1.24 | *.peg.3466 |
|  |  | Hemoprotein HemQ, essential component of heme biosynthetic pathway in Gram-positive bacteria |  | *.peg.4716 |
|  |  | Glutamyl-tRNA reductase | 1.2.1.70 | *.peg.3469 |
|  |  | Porphobilinogen deaminase | 2.5.1.61 | *.peg.3468 |
|  |  | Uroporphyrinogen-III methyltransferase | 2.1.1.107 | *.peg.3054, *.peg.3467, *.peg.4887 |
|  |  | Ferrochelatase, protoheme ferro-lyase | 4.99.1.1 | *.peg.4432 |
|  |  | Uroporphyrinogen III decarboxylase | 4.1.1.37 | *.peg.4718 |
|  |  | Protoporphyrinogen IX oxidase, aerobic, HemY | 1.3.3.4 | *.peg.2138, *.peg.4717 |
|  |  | Sirohydrochlorin ferrochelatase | 4.99.1.4 | *.peg.931, *.peg.1923, *.peg.4887 |
|  | Riboflavin (vitamin B2) metabolism | FMN adenylyltransferase | 2.7.7.2 | *.peg.4846 |
|  |  | FIG000859: hypothetical protein YebC |  | *.peg.4632 |
|  |  | Molybdopterin binding motif, CinA N-terminal domain |  | *.peg.1251 |
|  |  | C-terminal domain of CinA type S |  | *.peg.1251 |
|  |  | 6,7-dimethyl-8-ribityllumazine synthase | 2.5.1.78 | *.peg.4501 |
|  |  | 5-amino-6-(5-phosphoribosylamino)uracil reductase | 1.1.1.193 | *.peg.4509 |
|  |  | tRNA pseudouridine synthase B | 4.2.1.70 | *.peg.4849 |
|  |  | Riboflavin kinase | 2.7.1.26 | *.peg.4846 |
|  |  | GTP cyclohydrolase II | 3.5.4.25 | *.peg.2442, *.peg.4502, *.peg.5645 |
|  |  | Riboflavin synthase eubacterial/eukaryotic | 2.5.1.9 | *.peg.4505 |
|  |  | Diaminohydroxyphosphoribosylaminopyrimidine deaminase | 3.5.4.26 | *.peg.4509 |
|  |  | 3,4-dihydroxy-2-butanone 4-phosphate synthase | 4.1.99.12 | *.peg.2442, *.peg.4502, *.peg.5645 |
|  | Pyridoxin (vitamin B6) biosynthesis | Pyridoxine biosynthesis glutamine amidotransferase, synthase subunit | 2.4.2.- | *.peg.4643, *.peg.5691 |
|  |  | D-3-phosphoglycerate dehydrogenase | 1.1.1.95 | *.peg.2406, *.peg.2454, *.peg.3672, *.peg.3681, *.peg.5241, *.peg.5253, *.peg.5489, *.peg.6418, *.peg.6516 |
|  |  | 4-hydroxythreonine-4-phosphate dehydrogenase | 1.1.1.262 | *.peg.2852 |
|  |  | Pyridoxamine 5'-phosphate oxidase | 1.4.3.5 | *.peg.3457, *.peg.4117, *.peg.5768, *.peg.6137 |
|  |  | Phosphoserine aminotransferase | 2.6.1.52 | *.peg.4108 |
|  |  | 1-deoxy-D-xylulose 5-phosphate synthase | 2.2.1.7 | *.peg.4722 |
|  |  | Pyridoxine biosynthesis glutamine amidotransferase, glutaminase subunit | 2.4.2.- | *.peg.4641 |
|  |  | NAD-dependent glyceraldehyde-3-phosphate dehydrogenase | 1.2.1.12 | *.peg.4489 |
|  | Folate (vitamin B9) biosynthesis | Ketopantoate reductase PanG | 1.1.1.169 | *.peg.3700 |
|  |  | Dihydropteroate synthase | 2.5.1.15 | *.peg.3695 |
|  |  | tRNA(Ile)-lysidine synthetase | 6.3.4.19 | *.peg.3687 |
|  |  | Hypoxanthine-guanine phosphoribosyltransferase | 2.4.2.8 | *.peg.3688 |
|  |  | FIG027937: secreted protein |  | *.peg.819, *.peg.3413, *.peg.3698 |
|  |  | Aspartate 1-decarboxylase | 4.1.1.11 | *.peg.3702, *.peg.5014 |
|  |  | membrane-flanked domain |  | *.peg.2768 |
|  |  | Cell division protein FtsH | 3.4.24.- | *.peg.3548, *.peg.3693 |
|  |  | GTP cyclohydrolase I | 3.5.4.16 type 1 | *.peg.3694 |
|  |  | transmembrane protein, distant homology with ydbS |  | *.peg.3543 |
|  |  | Pantoate--beta-alanine ligase | 6.3.2.1 | *.peg.3701 |
|  |  | 2-amino-4-hydroxy-6-hydroxymethyldihydropteridine pyrophosphokinase | 2.7.6.3 | *.peg.3697 |
|  |  | Dihydroneopterin aldolase | 4.1.2.25 | *.peg.3696 |
|  |  | Aminodeoxychorismate lyase | 4.1.3.38 | *.peg.3980 |
|  |  | Folylpolyglutamate synthase | 6.3.2.17 | *.peg.1764 |
|  |  | 5-formyltetrahydrofolate cyclo-ligase | 6.3.3.2 | *.peg.4288 |
|  |  | Dihydrofolate reductase | 1.5.1.3 | *.peg.884, *.peg.4826, *.peg.5126, *.peg.6416 |
|  |  | Dihydrofolate synthase | 6.3.2.12 | *.peg.1764 |
|  |  | Para-aminobenzoate synthase, aminase component | 2.6.1.85 | *.peg.4320 |
|  |  | Thymidylate synthase | 2.1.1.45 | *.peg.4827 |
|  |  | Para-aminobenzoate synthase, amidotransferase component | 2.6.1.85 | *.peg.1645, *.peg.5955 |
| Cell Wall/ Secretion | Amino acid and peptide ABC transporter | Oligopeptide transport system permease protein OppC | 3.A.1.5.1 | *.peg.1905, *.peg.6167 |
|  |  | Oligopeptide transport system permease protein OppB | 3.A.1.5.1 | *.peg.19, *.peg.3676, *.peg.5431, *.peg.6166, *.peg.6168 |
|  |  | Oligopeptide transport ATP-binding protein OppF | 3.A.1.5.1 | *.peg.2859, *.peg.3677, *.peg.5430 |
|  |  | Oligopeptide transport ATP-binding protein OppD | 3.A.1.5.1 | *.peg.2858 |
|  |  | Oligopeptide ABC transporter, periplasmic oligopeptide-binding protein OppA | 3.A.1.5.1 | *.peg.1226, *.peg.2027, *.peg.4619, *.peg.4669, *.peg.5434, *.peg.5541, *.peg.6169 |
|  |  | High-affinity branched-chain amino acid transport system permease protein LivH | 3.A.1.4.1 | *.peg.2402 |
|  |  | Branched-chain amino acid transport ATP-binding protein LivF | 3.A.1.4.1 | *.peg.2405 |
|  |  | Branched-chain amino acid transport system permease protein LivM | 3.A.1.4.1 | *.peg.2403 |
|  |  | Branched-chain amino acid ABC transporter, amino acid-binding protein | 3.A.1.4.1 | *.peg.2401 |
|  |  | Branched-chain amino acid transport ATP-binding protein LivG | 3.A.1.4.1 | *.peg.2404 |
|  |  | Dipeptide transport system permease protein DppB | 3.A.1.5.2 | *.peg.1227, *.peg.1612, *.peg.1904, *.peg.2028, *.peg.4666, *.peg.5432, *.peg.5544 |
|  |  | Dipeptide transport ATP-binding protein DppD | 3.A.1.5.2 | *.peg.1229, *.peg.1610, *.peg.1906, *.peg.2030, *.peg.3513, *.peg.4668 |
|  |  | Dipeptide transport system permease protein DppC | 3.A.1.5.2 | *.peg.1228, *.peg.1611, *.peg.2029, *.peg.2857, *.peg.3514, *.peg.3675, *.peg.4667, *.peg.5433, *.peg.5543 |
|  |  | Dipeptide-binding ABC transporter, periplasmic substrate-binding component | 3.A.1.5.2 | *.peg.1613, *.peg.2855 |
| Nitrogen | Nitrate reductase | Assimilatory nitrate reductase large subunit | 1.7.99.4 | *.peg.213, *.peg.216 |
|  | Nitrite reductase | Nitrite reductase [NAD(P)H] small subunit | 1.7.1.4 | *.peg.211, *.peg.929 |
|  |  | Nitrite reductase [NAD(P)H] large subunit | 1.7.1.4 | *.peg.212, *.peg.928 |
|  | Nitrate/ite transporter | Nitrate/nitrite transporter |  | *.peg.215, *.peg.2199 |
|  |  | Nitrite transporter from formate/nitrite family |  | *.peg.823, *.peg.3028 |
|  |  | ABC-type nitrate/sulfonate/bicarbonate transport system, permease component |  | *.peg.1692, *.peg.4803, *.peg.6039 |
|  |  | ABC-type nitrate/sulfonate/bicarbonate transport system, ATPase component |  | *.peg.689, *.peg.4805, *.peg.5386, *.peg.6041 |
|  | Nitric oxide reductase | Nitric oxide reductase activation protein NorD |  | *.peg.1096, *.peg.5713 |
|  |  | Nitric oxide reductase activation protein NorQ |  | *.peg.1095, *.peg.5714 |
|  |  | Anaerobic nitric oxide reductase flavorubredoxin |  | *.peg.5382 |
|  | Ammonium transporter | Ammonium transporter |  | *.peg.542, *.peg.1433, *.peg.1758, *.peg.6348 |
|  | Glutamine synthetase | Glutamine synthetase type I | 6.3.1.2 | *.peg.541, *.peg.1646, *.peg.2276, *.peg.2282, *.peg.5109, *.peg.6518 |
|  |  | Glutamine synthetase type III, GlnN | 6.3.1.2 | *.peg.1712 |
|  |  | Glutamine synthetase family protein in hypothetical Actinobacterial gene cluster |  | *.peg.4900 |
|  | Glutamate synthase | Glutamate synthase [NADPH] large chain | 1.4.1.13 | *.peg.538, *.peg.1061, *.peg.2372, *.peg.5223, *.peg.5288 |
|  |  | Glutamate synthase [NADPH] small chain | 1.4.1.13 | *.peg.1062, *.peg.2373 |
|  |  | Glutamate synthase [NADPH] putative GlxC chain | 1.4.1.13 | *.peg.539 |
| Carbo- hydrates | Trehalose biosynthesis | Malto-oligosyltrehalose synthase | 5.4.99.15 | *.peg.4385 |
|  |  | 1,4-alpha-glucan (glycogen) branching enzyme, GH-13-type | 2.4.1.18 | *.peg.1408 |
|  |  | Trehalose phosphorylase | 2.4.1.64 | *.peg.2976 |
|  |  | Trehalose-6-phosphate phosphatase | 3.1.3.12 | *.peg.3761 |
|  |  | Putative glucanase glgE | 3.2.1.- | *.peg.1409 |
|  |  | Malto-oligosyltrehalose trehalohydrolase | 3.2.1.141 | *.peg.4386 |
|  |  | Glycogen debranching enzyme | 3.2.1.- | *.peg.4384, *.peg.5606 |
|  |  | Alpha,alpha-trehalose-phosphate synthase [UDP-forming] | 2.4.1.15 | *.peg.3898 |
|  |  | Glucoamylase | 3.2.1.3 | *.peg.1913, *.peg.5612 |
| **Myco. P9-64 (fig\|6666666.353107)** | | |  |  |
| Category | Subsytem | Role/ Protein | EC number | Protein encoding genes |
| Vitamins/ Cofactors | Biotin (vitamin B7) biosynthesis | Competence protein F homolog, phosphoribosyltransferase domain |  | *.peg.6964 |
|  |  | Adenosylmethionine-8-amino-7-oxononanoate aminotransferase | 2.6.1.62 | *.peg.2370, *.peg.3589, *.peg.5126 |
|  |  | Biotin-protein ligase | 6.3.4.15 | *.peg.7020 |
|  |  | 8-amino-7-oxononanoate synthase | 2.3.1.47 | *.peg.3588 |
|  |  | Dethiobiotin synthetase | 6.3.3.3 | *.peg.3587 |
|  |  | Biotin synthase | 2.8.1.6 | *.peg.3583 |
|  |  | Long-chain-fatty-acid--CoA ligase | 6.2.1.3 | *.peg.117, *.peg.361, *.peg.555, *.peg.822, *.peg.1101, *.peg.1579, *.peg.1600, *.peg.1638, *.peg.3952, *.peg.5061, *.peg.5280, *.peg.6093, *.peg.6104, *.peg.6376, *.peg.6684, *.peg.6695, *.peg.6856, *.peg.6866, *.peg.6867 |
|  |  | 3-ketoacyl-CoA thiolase | 2.3.1.16 | *.peg.159, *.peg.771, *.peg.1657, *.peg.3373, *.peg.3786, *.peg.4182, *.peg.4233, *.peg.4772, *.peg.5120, *.peg.5276, *.peg.5872, *.peg.6098, *.peg.6221, *.peg.6464, *.peg.6505, *.peg.6524, *.peg.6613, *.peg.6845 |
|  |  | tRNA (cytidine(34)-2'-O)-methyltransferase | 2.1.1.207 | *.peg.999 |
|  |  | ATPase component BioM of energizing module of biotin ECF transporter |  | *.peg.4268 |
|  |  | Predicted biotin repressor from TetR family |  | *.peg.3584 |
|  |  | predicted biotin regulatory protein BioR (GntR family) |  | *.peg.1580 |
|  | Thiamin (vitamin B1) biosynthesis | Sulfur carrier protein adenylyltransferase ThiF |  | *.peg.4480, *.peg.6929 |
|  |  | Hydroxymethylpyrimidine ABC transporter, substrate-binding component |  | *.peg.1256 |
|  |  | Glycine oxidase ThiO | 1.4.3.19 | *.peg.2064 |
|  |  | Thiazole biosynthesis protein ThiG |  | *.peg.2066 |
|  |  | Thiamine-monophosphate kinase | 2.7.4.16 | *.peg.6029 |
|  |  | 1-deoxy-D-xylulose 5-phosphate synthase | 2.2.1.7 | *.peg.3927 |
|  |  | Hydroxymethylpyrimidine ABC transporter, ATPase component |  | *.peg.837 |
|  |  | Thiamin-phosphate pyrophosphorylase | 2.5.1.3 | *.peg.2062 |
|  |  | Thiamin ABC transporter, transmembrane component |  | *.peg.2976 |
|  |  | Sulfur carrier protein ThiS |  | *.peg.2065 |
|  | Mena- quinone and Phyllo- quinone (vitamin K1 & K2) biosynthesis | Naphthoate synthase | 4.1.3.36 | *.peg.2264 |
|  |  | 2-succinyl-6-hydroxy-2,4-cyclohexadiene-1-carboxylate synthase | 4.2.99.20 | *.peg.2279 |
|  |  | 2-succinyl-5-enolpyruvyl-6-hydroxy-3-cyclohexene-1-carboxylic-acid synthase | 2.2.1.9 | *.peg.2280 |
|  |  | 2-heptaprenyl-1,4-naphthoquinone methyltransferase | 2.1.1.163 | *.peg.2289 |
|  |  | O-succinylbenzoic acid--CoA ligase | 6.2.1.26 | *.peg.883, *.peg.2249, *.peg.2269, *.peg.2273 |
|  |  | O-succinylbenzoate synthase | 4.2.1.113 | *.peg.2274, *.peg.5630 |
|  |  | 1,4-dihydroxy-2-naphthoate polyprenyltransferase | 2.5.1.74 | *.peg.2231 |
|  |  | Ubiquinone/Mena- quinone biosynthesis methyltransferase UbiE | 2.1.1.- | *.peg.4591 |
|  | Cobalamin (vitamin B12) synthesis | Cobalt-precorrin-6x reductase | 1.3.1.54 | *.peg.5345 |
|  |  | Cobalamin biosynthesis protein BluB |  | *.peg.3337 |
|  |  | Adenosylcobinamide-phosphate guanylyltransferase | 2.7.7.62 | *.peg.4696 |
|  |  | Cobalt-precorrin-8x methylmutase | 5.4.1.2 | *.peg.5348 |
|  |  | Cobalt-precorrin-2 C20-methyltransferase | 2.1.1.130 | *.peg.5347 |
|  |  | Cobyric acid synthase | 6.3.5.10 | *.peg.6171 |
|  |  | Cobalt-precorrin-4 C11-methyltransferase | 2.1.1.133 | *.peg.5344 |
|  |  | Cobalt-precorrin-3b C17-methyltransferase |  | *.peg.5347 |
|  |  | Nicotinate-nucleotide--dimethylbenzimidazole phosphoribosyltransferase | 2.4.2.21 | *.peg.4695 |
|  |  | Adenosylcobinamide-phosphate synthase | 6.3.1.10 | *.peg.4650 |
|  |  | Cob(I)alamin adenosyltransferase | 2.5.1.17 | *.peg.6188 |
|  |  | Cobalamin synthase | 2.7.8.26 | *.peg.4694 |
|  |  | Cobyrinic acid A,C-diamide synthase |  | *.peg.6189 |
|  |  | CblZ, a non-orthologous displasment for Alpha-ribazole-5'-phosphate phosphatase |  | *.peg.4697 |
|  |  | Cobalt-precorrin-6y C5-methyltransferase | 2.1.1.- | *.peg.5343 |
|  |  | 5,6-dimethylbenzimidazole synthase, flavin destructase family |  | *.peg.3337 |
|  |  | Cobalt-precorrin-6y C15-methyltransferase [decarboxylating] | 2.1.1.- | *.peg.5343 |
|  |  | Uroporphyrinogen-III methyltransferase | 2.1.1.107 | *.peg.2208, *.peg.6190 |
|  |  | ChlI component of cobalt chelatase involved in B12 biosynthesis |  | *.peg.6187 |
|  |  | Precorrin-6A synthase | 2.1.1.152 | *.peg.5228 |
|  |  | ChlD component of cobalt chelatase involved in B12 biosynthesis |  | *.peg.6187 |
|  |  | Cobalamin biosynthesis protein CobG |  | *.peg.5349 |
|  |  | Predicted cobalt transporter CbtA |  | *.peg.3312 |
|  |  | CobN component of cobalt chelatase involved in B12 biosynthesis |  | *.peg.5352 |
|  | Heme and Siroheme biosynthesis | Hypothetical radical SAM family enzyme in heat shock gene cluster, similarity with CPO of BS HemN-type |  | *.peg.4503 |
|  |  | Glutamyl-tRNA synthetase | 6.1.1.17 | *.peg.6016 |
|  |  | Uroporphyrinogen-III synthase | 4.2.1.75 | *.peg.1684, *.peg.2208 |
|  |  | Glutamate-1-semialdehyde aminotransferase | 5.4.3.8 | *.peg.1165, *.peg.1644, *.peg.2076, *.peg.2221, *.peg.6001 |
|  |  | Precorrin-2 oxidase | 1.3.1.76 | *.peg.6190 |
|  |  | Porphobilinogen synthase | 4.2.1.24 | *.peg.2209 |
|  |  | Hemoprotein HemQ, essential component of heme biosynthetic pathway in Gram-positive bacteria |  | *.peg.3921 |
|  |  | Glutamyl-tRNA reductase | 1.2.1.70 | *.peg.2206 |
|  |  | Porphobilinogen deaminase | 2.5.1.61 | *.peg.2207 |
|  |  | Uroporphyrinogen-III methyltransferase | 2.1.1.107 | *.peg.2208, *.peg.6190 |
|  |  | Ferrochelatase, protoheme ferro-lyase | 4.99.1.1 | *.peg.3632 |
|  |  | Uroporphyrinogen III decarboxylase | 4.1.1.37 | *.peg.3923 |
|  |  | Protoporphyrinogen IX oxidase, aerobic, HemY | 1.3.3.4 | *.peg.3922 |
|  |  | Sirohydrochlorin ferrochelatase | 4.99.1.4 | *.peg.1683, *.peg.4500, *.peg.6190 |
|  | Riboflavin (vitamin B2) metabolism | FMN adenylyltransferase | 2.7.7.2 | *.peg.6227 |
|  |  | FIG000859: hypothetical protein YebC |  | *.peg.3833 |
|  |  | Molybdopterin binding motif, CinA N-terminal domain |  | *.peg.5665 |
|  |  | C-terminal domain of CinA type S |  | *.peg.5665 |
|  |  | 6,7-dimethyl-8-ribityllumazine synthase | 2.5.1.78 | *.peg.3693 |
|  |  | 5-amino-6-(5-phosphoribosylamino)uracil reductase | 1.1.1.193 | *.peg.3700 |
|  |  | tRNA pseudouridine synthase B | 4.2.1.70 | *.peg.6216 |
|  |  | Riboflavin kinase | 2.7.1.26 | *.peg.6227 |
|  |  | GTP cyclohydrolase II | 3.5.4.25 | *.peg.3694, *.peg.4042, *.peg.5158, *.peg.5487 |
|  |  | Riboflavin synthase eubacterial/eukaryotic | 2.5.1.9 | *.peg.3695 |
|  |  | Diaminohydroxyphosphoribosylaminopyrimidine deaminase | 3.5.4.26 | *.peg.3700 |
|  |  | 3,4-dihydroxy-2-butanone 4-phosphate synthase | 4.1.99.12 | *.peg.3694, *.peg.4042, *.peg.5158, *.peg.5487 |
|  | Pyridoxin (vitamin B6) biosynthesis | Pyridoxine biosynthesis glutamine amidotransferase, synthase subunit | 2.4.2.- | *.peg.3838 |
|  |  | Pyridoxine biosynthesis glutamine amidotransferase, glutaminase subunit | 2.4.2.- | *.peg.3836 |
|  |  | D-3-phosphoglycerate dehydrogenase | 1.1.1.95 | *.peg.1795, *.peg.2474, *.peg.2696, *.peg.3272, *.peg.3281, *.peg.4086, *.peg.5996 |
|  |  | Pyridoxamine 5'-phosphate oxidase | 1.4.3.5 | *.peg.302, *.peg.2217, *.peg.2262, *.peg.5034 |
|  |  | Phosphoserine aminotransferase | 2.6.1.52 | *.peg.5022 |
|  |  | NAD-dependent glyceraldehyde-3-phosphate dehydrogenase | 1.2.1.12 | *.peg.3680 |
|  |  | 1-deoxy-D-xylulose 5-phosphate synthase | 2.2.1.7 | *.peg.3927 |
|  | Folate (vitamin B9) biosynthesis | Ketopantoate reductase PanG | 1.1.1.169 | *.peg.3303 |
|  |  | Dihydropteroate synthase | 2.5.1.15 | *.peg.3298 |
|  |  | tRNA(Ile)-lysidine synthetase | 6.3.4.19 | *.peg.3288 |
|  |  | Hypoxanthine-guanine phosphoribosyltransferase | 2.4.2.8 | *.peg.3289 |
|  |  | FIG027937: secreted protein |  | *.peg.3301, *.peg.4596, *.peg.6435 |
|  |  | Aspartate 1-decarboxylase | 4.1.1.11 | *.peg.3305, *.peg.3870 |
|  |  | membrane-flanked domain |  | *.peg.7019 |
|  |  | Cell division protein FtsH | 3.4.24.- | *.peg.2126, *.peg.2630, *.peg.3296 |
|  |  | GTP cyclohydrolase I | 3.5.4.16 type 1 | *.peg.3297 |
|  |  | transmembrane protein, distant homology with ydbS |  | *.peg.2131 |
|  |  | Pantoate--beta-alanine ligase | 6.3.2.1 | *.peg.3304 |
|  |  | 2-amino-4-hydroxy-6-hydroxymethyldihydropteridine pyrophosphokinase | 2.7.6.3 | *.peg.3300 |
|  |  | Dihydroneopterin aldolase | 4.1.2.25 | *.peg.3299 |
|  |  | Aminodeoxychorismate lyase | 4.1.3.38 | *.peg.4878 |
|  |  | Folylpolyglutamate synthase | 6.3.2.17 | *.peg.4418 |
|  |  | 5-formyltetrahydrofolate cyclo-ligase | 6.3.3.2 | *.peg.6313 |
|  |  | Dihydrofolate reductase | 1.5.1.3 | *.peg.1610, *.peg.2842, *.peg.5728, *.peg.6254 |
|  |  | Dihydrofolate synthase | 6.3.2.12 | *.peg.4418 |
|  |  | Para-aminobenzoate synthase, aminase component | 2.6.1.85 | *.peg.6365 |
|  |  | Thymidylate synthase | 2.1.1.45 | *.peg.6253 |
|  |  | Para-aminobenzoate synthase, amidotransferase component | 2.6.1.85 | *.peg.78, *.peg.1729 |
| Cell Wall/ Secretion | Amino acid and peptide ABC transporter | Oligopeptide transport system permease protein OppC | 3.A.1.5.1 | *.peg.415 |
|  |  | Oligopeptide transport system permease protein OppB | 3.A.1.5.1 | *.peg.414, *.peg.416, *.peg.1211, *.peg.2722, *.peg.3276, *.peg.3969, *.peg.3984, *.peg.5250, *.peg.5251, *.peg.6007 |
|  |  | Oligopeptide transport ATP-binding protein OppF | 3.A.1.5.1 | *.peg.1848, *.peg.2721, *.peg.3277 |
|  |  | Oligopeptide transport ATP-binding protein OppD | 3.A.1.5.1 | *.peg.1849 |
|  |  | Oligopeptide ABC transporter, periplasmic oligopeptide-binding protein OppA | 3.A.1.5.1 | *.peg.417, *.peg.418, *.peg.1852, *.peg.2718, *.peg.3820, *.peg.3986, *.peg.5247, *.peg.5544, *.peg.6003 |
|  |  | High-affinity branched-chain amino acid transport system permease protein LivH | 3.A.1.4.1 | *.peg.427, *.peg.3526 |
|  |  | Branched-chain amino acid transport ATP-binding protein LivF | 3.A.1.4.1 | *.peg.429, *.peg.3523 |
|  |  | Branched-chain amino acid transport system permease protein LivM | 3.A.1.4.1 | *.peg.3525 |
|  |  | Branched-chain amino acid ABC transporter, amino acid-binding protein | 3.A.1.4.1 | *.peg.3527 |
|  |  | Branched-chain amino acid transport ATP-binding protein LivG | 3.A.1.4.1 | *.peg.428, *.peg.3524 |
|  |  | Dipeptide transport system permease protein DppB | 3.A.1.5.2 | *.peg.1209, *.peg.1850, *.peg.2719, *.peg.5543, *.peg.6005 |
|  |  | Dipeptide transport ATP-binding protein DppD | 3.A.1.5.2 | *.peg.5541 |
|  |  | Dipeptide transport system permease protein DppC | 3.A.1.5.2 | *.peg.1210, *.peg.1851, *.peg.2720, *.peg.3275, *.peg.3970, *.peg.3983, *.peg.5542, *.peg.6006 |
|  |  | Dipeptide-binding ABC transporter, periplasmic substrate-binding component | 3.A.1.5.2 | *.peg.1207 |
| Nitrogen | Nitrate reductase | Respiratory nitrate reductase alpha chain | 1.7.99.4 | *.peg.310 |
|  |  | Assimilatory nitrate reductase large subunit | 1.7.99.4 | *.peg.2497 |
|  |  | Respiratory nitrate reductase gamma chain | 1.7.99.4 | *.peg.307 |
|  |  | Respiratory nitrate reductase beta chain | 1.7.99.4 | *.peg.309 |
|  |  | Respiratory nitrate reductase delta chain | 1.7.99.4 | *.peg.308 |
|  | Nitrite reductase | Nitrite reductase [NAD(P)H] large subunit | 1.7.1.4 | *.peg.1680 |
|  |  | Nitrite reductase [NAD(P)H] small subunit | 1.7.1.4 | *.peg.1681 |
|  | Nitrate/ite transporter | Nitrate/nitrite transporter |  | *.peg.311, *.peg.2309, *.peg.2496, *.peg.6710 |
|  |  | ABC-type nitrate/sulfonate/bicarbonate transport system, permease component |  | *.peg.436, *.peg.836, *.peg.1253, *.peg.2485 |
|  |  | ABC-type nitrate/sulfonate/bicarbonate transport system, ATPase component |  | *.peg.2483 |
|  | Nitric oxide reductase | Anaerobic nitric oxide reductase flavorubredoxin |  | *.peg.1980 |
|  | Ammonium transporter | Ammonium transporter |  | *.peg.1952, *.peg.6066 |
|  | Glutamine synthetase | Glutamine synthetase type I | 6.3.1.2 | *.peg.4468, *.peg.4676, *.peg.4683 |
|  |  | Glutamine synthetase type III, GlnN | 6.3.1.2 | *.peg.4369 |
|  |  | Glutamine synthetase family protein in hypothetical Actinobacterial gene cluster |  | *.peg.6176 |
|  |  | glutamine synthetase family protein |  | *.peg.799, *.peg.1731, *.peg.1823, *.peg.5775 |
|  | Glutamate synthase | Ferredoxin-dependent glutamate synthase | 1.4.7.1 | *.peg.3472 |
|  |  | Glutamate synthase [NADPH] large chain | 1.4.1.13 | *.peg.2877, *.peg.3549 |
|  |  | Glutamate synthase [NADPH] small chain | 1.4.1.13 | *.peg.2878, *.peg.3548 |
| Carbo- hydrates | Trehalose biosynthesis | Malto-oligosyltrehalose synthase | 5.4.99.15 | *.peg.3592 |
|  |  | Putative glucanase glgE | 3.2.1.- | *.peg.4186 |
|  |  | 1,4-alpha-glucan (glycogen) branching enzyme, GH-13-type | 2.4.1.18 | *.peg.4185 |
|  |  | Trehalose phosphorylase | 2.4.1.64 | *.peg.2751 |
|  |  | Trehalose synthase | 5.4.99.16 | *.peg.2830 |
|  |  | Trehalose-6-phosphate phosphatase | 3.1.3.12 | *.peg.340, *.peg.3344 |
|  |  | Alpha,alpha-trehalose-phosphate synthase [UDP-forming] | 2.4.1.15 | *.peg.4796 |
|  |  | Glycogen debranching enzyme | 3.2.1.- | *.peg.2838, *.peg.3591 |
|  |  | Malto-oligosyltrehalose trehalohydrolase | 3.2.1.141 | *.peg.3593 |
|  |  | Glucoamylase | 3.2.1.3 | *.peg.4487 |

Suppl. Table 4: Protein encoding genes predicted to be involved in plant growth promotion and resistance of strains P1-5, P1-18, P9-22 and P9-64 determined by RAST.

| **Myco. P1-5 (fig\|6666666.353102)** | | |  |  |
| --- | --- | --- | --- | --- |
| Category | Subsytem | Role/ Protein | EC number | Protein encoding genes |
| Siderophore / Fe uptake | Ferrous iron transporter | Ferrous iron transport peroxidase EfeB |  | *.peg.1197 |
|  |  | Ferrous iron transport permease EfeU |  | *.peg.1199 |
|  |  | Ferrous iron transport periplasmic protein EfeO, contains peptidase-M75 domain and (frequently) cupredoxin-like domain |  | *.peg.1198 |
| Phosphate solubilization | Phosphatase | Exopolyphosphatase | 3.6.1.11 | *.peg.1204, *.peg.3730 |
|  |  | Inorganic pyrophosphatase | 3.6.1.1 | *.peg.1432 |
| Phosphate uptake and transport | Low-affinity inorganic phosphate trans. system | Probable low-affinity inorganic phosphate transporter |  | *.peg.3669 |
|  | High-affinity phosphate transport system | Phosphate regulon transcriptional regulatory protein PhoB (SphR) |  | *.peg.1970, *.peg.3204, *.peg.3679, *.peg.3741, *.peg.4666 |
|  |  | Phosphate transport system permease protein PstC | 3.A.1.7.1 | *.peg.4663 |
|  |  | Phosphate transport ATP-binding protein PstB | 3.A.1.7.1 | *.peg.4661 |
|  |  | Phosphate transport system permease protein PstA | 3.A.1.7.1 | *.peg.4662 |
|  |  | Phosphate transport system regulatory protein PhoU |  | *.peg.4660 |
|  |  | Phosphate ABC transporter, periplasmic phosphate-binding protein PstS | 3.A.1.7.1 | *.peg.4664 |
|  |  | Polyphosphate kinase | 2.7.4.1 | *.peg.2382 |
|  |  | Phosphate regulon sensor protein PhoR (SphS) | 2.7.13.3 | *.peg.3742 |
| Polyamine | Putrescine/ spermidine synthesis | Putrescine transport ATP-binding protein PotG | 3.A.1.11.2 | *.peg.1528 |
|  |  | Putrescine transport system permease protein PotH | 3.A.1.11.2 | *.peg.1530 |
|  |  | Putrescine transport system permease protein PotI | 3.A.1.11.2 | *.peg.1531 |
|  |  | Putrescine ABC transporter putrescine-binding protein PotF | 3.A.1.11.2 | *.peg.1529 |
|  | Putrescine/ spermidine transport | Spermidine synthase | 2.5.1.16 | *.peg.5015 |
|  |  | Arginine decarboxylase | 4.1.1.19 | *.peg.1967 |
|  |  | Ornithine decarboxylase | 4.1.1.17 | *.peg.1967 |
|  |  | Agmatinase | 3.5.3.11 | *.peg.116 |
| Resistance to heavy metals | Cobalt-zinc-cadmium resistance | Cobalt-zinc-cadmium resistance protein CzcD |  | *.peg.3359, *.peg.4848 |
|  |  | Transcriptional regulator, MerR family |  | *.peg.1665, *.peg.3554, *.peg.5077 |
|  | Copper homeostasis/ tolerance | Multicopper oxidase |  | *.peg.4852 |
|  |  | Copper resistance protein CopD |  | *.peg.3245 |
|  |  | Copper-translocating P-type ATPase | 3.6.3.4 | *.peg.1276, *.peg.2159, *.peg.3708 |
|  |  | Copper resistance protein CopC |  | *.peg.1064, *.peg.3245 |
|  |  | Multidrug resistance transporter, Bcr/CflA family |  | *.peg.601 |
|  |  | Copper resistance protein D |  | *.peg.2145, *.peg.2283 |
|  | Arsenic resistance | Arsenical resistance operon repressor |  | *.peg.2142, *.peg.4051 |
|  |  | Arsenical pump-driving ATPase | 3.6.3.16 | *.peg.847, *.peg.848, *.peg.4560 |
|  |  | Arsenic efflux pump protein |  | *.peg.1477 |
|  |  | Arsenate reductase | 1.20.4.1 | *.peg.1189, *.peg.4053, *.peg.4054 |
|  | Mercury resistance & detoxification | PF00070 family, FAD-dependent NAD(P)-disulphide oxidoreductase |  | *.peg.3435, *.peg.4485 |
|  | Uptake of selenate/ selenite | Sulfate and thiosulfate import ATP-binding protein CysA | 3.6.3.25 | *.peg.204 |
|  |  | DedA protein |  | *.peg.1532, *.peg.3353, *.peg.4200 |
| Resistance to antibiotics | Fluoro-quinolone resistance | DNA gyrase subunit B | 5.99.1.3 | *.peg.4345 |
|  |  | DNA gyrase subunit A | 5.99.1.3 | *.peg.4346 |
|  |  | Topoisomerase IV subunit B | 5.99.1.- | *.peg.4217 |
|  |  | Topoisomerase IV subunit A | 5.99.1.- | *.peg.4218, *.peg.4231 |
|  | Beta-lactamase | Beta-lactamase class C and other penicillin binding proteins |  | *.peg.307, *.peg.1791, *.peg.2019 |
|  |  | Beta-lactamase | 3.5.2.6 | *.peg.452, *.peg.1461, *.peg.2094, *.peg.1831, *.peg.2831 |
|  |  | Metal-dependent hydrolases of the beta-lactamase superfamily III |  | *.peg.2579 |
| Antibiosis compounds | Clavulanic acid biosynthesis | clavaldehyde dehydrogenase |  | *.peg.2795 |
|  | Chitinase & β-hexosaminidase | Chitinase | 3.2.1.14 | *.peg.2112 |
|  |  | Beta-hexosaminidase | 3.2.1.52 | *.peg.3169 |
| Resistance to oxidative stress | Peroxidase | Ferrous iron transport peroxidase EfeB |  | *.peg.1197 |
|  |  | Predicted dye-decolorizing peroxidase (DyP), encapsulated subgroup |  | *.peg.4692 |
|  |  | Encapsulating protein for a DyP-type peroxidase or ferritin-like protein oligomers |  | *.peg.4693 |
|  |  | Peroxidase | 1.11.1.7 | *.peg.88, *.peg.5161 |
|  |  | Thiol peroxidase, Tpx-type | 1.11.1.15 | *.peg.2772 |
|  | Catalase | Catalase | 1.11.1.6 | *.peg.88, *.peg.894, *.peg.1864, *.peg.3604 |
|  | Superoxide dismutase | Superoxide dismutase [Cu-Zn] precursor | 1.15.1.1 | *.peg.3830, *.peg.4037 |
|  | Hydroperoxide reductase | Alkyl hydroperoxide reductase subunit C-like protein |  | *.peg.324, *.peg.1621 |
|  |  | Alkyl hydroperoxide reductase protein C | 1.6.4.- | *.peg.3684 |
|  |  | Alkylhydroperoxidase protein D |  | *.peg.3683 |
|  |  | Organic hydroperoxide resistance protein |  | *.peg.804, *.peg.893 |
|  | Glutathione-mediated detoxification | Lactoylglutathione lyase | 4.4.1.5 | *.peg.1125, *.peg.4052 |
|  |  | Hydroxyacylglutathione hydrolase | 3.1.2.6 | *.peg.1874 |
|  | Mycothiol | NADPH-dependent mycothiol reductase Mtr |  | *.peg.2137 |
|  |  | Formaldehyde dehydrogenase MscR, NAD/mycothiol-dependent | 1.2.1.66 | *.peg.305 |
|  |  | Putative hydrolase in cluster with formaldehyde/S-nitrosomycothiol reductase MscR |  | *.peg.304 |
|  |  | S-nitrosomycothiol reductase MscR |  | *.peg.305 |
|  |  | Mycothiol S-conjugate amidase Mca |  | *.peg.3984 |
|  |  | L-cysteine:1D-myo-inosityl 2-amino-2-deoxy-alpha-D-glucopyranoside ligase MshC |  | *.peg.4499 |
|  |  | Glycosyltransferase MshA involved in mycothiol biosynthesis | 2.4.1.- | *.peg.3745 |
|  |  | Uncharacterized protein Rv0487/MT0505 clustered with mycothiol biosynthesis gene |  | *.peg.3744 |
|  |  | Acetyl-CoA:Cys-GlcN-Ins acetyltransferase, mycothiol synthase MshD |  | *.peg.4665 |
|  |  | N-acetyl-1-D-myo-inosityl-2-amino-2-deoxy-alpha-D-glucopyranoside deacetylase MshB |  | *.peg.1248 |
| Heat/ cold shock | Heat shock protein/ chaperone | Hypothetical radical SAM family enzyme in heat shock gene cluster, similarity with CPO of BS HemN-type |  | *.peg.208 |
|  |  | HspR, transcriptional repressor of DnaK operon |  | *.peg.3322 |
|  |  | Heat-inducible transcription repressor HrcA |  | *.peg.214 |
|  |  | Chaperone protein DnaK |  | *.peg.3319 |
|  |  | Chaperone protein DnaJ |  | *.peg.215, *.peg.3321 |
|  |  | Ribosomal RNA small subunit methyltransferase E | 2.1.1.- | *.peg.216 |
|  |  | tmRNA-binding protein SmpB |  | *.peg.4901 |
|  |  | Heat shock protein GrpE |  | *.peg.3320 |
|  |  | Translation elongation factor LepA |  | *.peg.3397 |
|  |  | Ribosome-associated heat shock protein implicated in the recycling of the 50S subunit (S4 paralog) |  | *.peg.4933 |
|  |  | Nucleoside 5-triphosphatase RdgB (dHAPTP, dITP, XTP-specific) | 3.6.1.15 | *.peg.2581 |
|  |  | Ribonuclease PH | 2.7.7.56 | *.peg.2580 |
|  |  | rRNA small subunit methyltransferase I |  | *.peg.1170 |
|  |  | Heat shock protein 60 family co-chaperone GroES |  | *.peg.2858 |
|  |  | Heat shock protein 60 family chaperone GroEL |  | *.peg.2859, *.peg.3798 |
|  | Cold shock protein | Cold shock protein CspA |  | *.peg.1410, *.peg.3539 |
|  |  | Cold shock protein CspC |  | *.peg.395 |
| Salt tolerance | Choline/ betaine uptake & biosynthesis | L-proline glycine betaine ABC transport system permease protein ProV | 3.A.1.12.1 | *.peg.956 |
|  |  | Glycine betaine ABC transport system permease protein |  | *.peg.954 |
|  |  | L-proline glycine betaine binding ABC transporter protein ProX | 3.A.1.12.1 | *.peg.957 |
|  |  | Choline-sulfatase | 3.1.6.6 | *.peg.3223 |
|  |  | Choline dehydrogenase | 1.1.99.1 | *.peg.4707 |
|  |  | L-proline glycine betaine ABC transport system permease protein ProW | 3.A.1.12.1 | *.peg.955 |
|  |  | Betaine aldehyde dehydrogenase | 1.2.1.8 | *.peg.1259 |
|  | K+ /Na+ transport | Osmosensitive K+ channel histidine kinase KdpD | 2.7.3.- | *.peg.539, *.peg.974, *.peg.1193, *.peg.1971, *.peg.3203 |
|  |  | Potassium channel protein |  | *.peg.461, *.peg.2766 |
|  |  | Potassium efflux system KefA protein |  | *.peg.3636, *.peg.4905 |
|  |  | Trk system potassium uptake protein TrkA |  | *.peg.2005, *.peg.2006, *.peg.2005, *.peg.2006 |
|  |  | Glutathione-regulated potassium-efflux system protein KefB |  | *.peg.5092 |
|  |  | Kup system potassium uptake protein |  | *.peg.2819 |
|  |  | Large-conductance mechanosensitive channel |  | *.peg.1126 |
|  |  | Na(+) H(+) antiporter subunit A |  | *.peg.3819 |
|  |  | Na(+) H(+) antiporter subunit B |  | *.peg.3819 |
|  |  | Na(+) H(+) antiporter subunit C |  | *.peg.3820 |
|  |  | Na(+) H(+) antiporter subunit D |  | *.peg.3821 |
|  |  | Na(+) H(+) antiporter subunit E |  | *.peg.3822 |
|  |  | Na(+) H(+) antiporter subunit F |  | *.peg.3823 |
|  |  | Na(+) H(+) antiporter subunit G |  | *.peg.3824 |
|  |  | Na+/H+ antiporter NhaA type |  | *.peg.2000, *.peg.3341 |
| Protection from UV radiation and oxidative stress | Carotenoids | Phytoene dehydrogenase and related proteins |  | *.peg.2394, *.peg.5122 |
|  |  | Phytoene synthase | 2.5.1.32 | *.peg.290 |
|  |  | Geranylgeranyl diphosphate synthase | 2.5.1.29 | *.peg.3638 |
|  |  | CrtT-methyltransferase-like protein |  | *.peg.293, *.peg.3344 |
|  |  | Phytoene dehydrogenase | 1.14.99.- | *.peg.289 |
|  |  | CrtV-methyltransferase-like protein |  | *.peg.295 |
|  |  | Beta-carotene ketolase | 1.14.-.- | *.peg.368, *.peg.3640 |
|  |  | Lycopene cyclase |  | *.peg.291, *.peg.292 |
|  |  | (2E,6E)-farnesyl diphosphate synthase | 2.5.1.10 | *.peg.1232, *.peg.3638 |
| **Myco. P1-18 (fig\|6666666.353104)** | | |  |  |
| Category | Subsytem | Role/ Protein | EC number | Protein encoding genes |
| Siderophore / Fe uptake | Siderohore receptors/ transport | ABC-type Fe3+-siderophore transport system, permease 2 component |  | *.peg.2323 |
|  | Ferrous iron transporter | Ferrous iron transport permease EfeU, N-terminal extended |  | *.peg.3646 |
|  |  | Ferrous iron transport peroxidase EfeB |  | *.peg.945, *.peg.3648 |
|  |  | Ferrous iron transport permease EfeU |  | *.peg.947 |
|  |  | Ferrous iron transport periplasmic protein EfeO, contains peptidase-M75 domain and (frequently) cupredoxin-like domain |  | *.peg.946, *.peg.3647 |
| Phosphate solubilization | Phosphatase | Exopolyphosphatase | 3.6.1.11 | *.peg.952, *.peg.1810 |
|  |  | Inorganic pyrophosphatase | 3.6.1.1 | *.peg.5080 |
|  |  | Alkaline phosphatase | 3.1.3.1 | *.peg.1410 |
| Phosphate uptake and transport | Low-affinity inorganic phosphate trans. system | Probable low-affinity inorganic phosphate transporter |  | *.peg.1716 |
|  | High-affinity phosphate transport system | Phosphate regulon transcriptional regulatory protein PhoB (SphR) |  | *.peg.1769, *.peg.1826, *.peg.5280, *.peg.5393 |
|  |  | Phosphate transport system permease protein PstC | 3.A.1.7.1 | *.peg.5396 |
|  |  | Phosphate transport ATP-binding protein PstB | 3.A.1.7.1 | *.peg.5398 |
|  |  | Phosphate transport system permease protein PstA | 3.A.1.7.1 | *.peg.5397 |
|  |  | Phosphate transport system regulatory protein PhoU |  | *.peg.1486, *.peg.1748, *.peg.5401 |
|  |  | Phosphate ABC transporter, periplasmic phosphate-binding protein PstS | 3.A.1.7.1 | *.peg.5395 |
|  |  | Polyphosphate kinase | 2.7.4.1 | *.peg.2548 |
|  |  | Phosphate regulon sensor protein PhoR (SphS) | 2.7.13.3 | *.peg.1827 |
| Plant hormone | Auxin biosynthesis | Tryptophan synthase alpha chain | 4.2.1.20 | *.peg.3215 |
|  |  | Tryptophan synthase beta chain | 4.2.1.20 | *.peg.3214 |
|  |  | Monoamine oxidase (1.4.3.4) |  | *.peg.1217, *.peg.2631, *.peg.6013 |
| Polyamine | Putrescine/ spermidine synthesis | Putrescine transport ATP-binding protein PotG | 3.A.1.11.2 | *.peg.5995 |
|  |  | Spermidine Putrescine ABC transporter permease component potC | 3.A.1.11.1 | *.peg.774, *.peg.1943, *.peg.5822, *.peg.5850 |
|  |  | Spermidine Putrescine ABC transporter permease component PotB | 3.A.1.11.1 | *.peg.773, *.peg.1942, *.peg.5823 |
|  |  | Putrescine transport system permease protein PotH | 3.A.1.11.2 | *.peg.5851, *.peg.5997 |
|  |  | Putrescine transport ATP-binding protein PotA | 3.A.1.11.1 | *.peg.771, *.peg.1939, *.peg.5825, *.peg.5853 |
|  |  | Putrescine transport system permease protein PotI | 3.A.1.11.2 | *.peg.5998 |
|  |  | Putrescine ABC transporter putrescine-binding protein PotF | 3.A.1.11.2 | *.peg.5996 |
|  |  | ABC transporter, periplasmic spermidine putrescine-binding protein PotD | 3.A.1.11.1 | *.peg.772, *.peg.1941, *.peg.5824, *.peg.5852 |
|  | Putrescine/ spermidine transport | Agmatinase | 3.5.3.11 | *.peg.432, *.peg.438, *.peg.439, *.peg.2714, *.peg.3932, *.peg.5720 |
|  |  | Agmatine deiminase | 3.5.3.12 | *.peg.2149, *.peg.3501 |
|  |  | Arginine decarboxylase | 4.1.1.19 | *.peg.2421, *.peg.2758 |
|  |  | Ornithine decarboxylase | 4.1.1.17 | *.peg.2758 |
| Resistance to heavy metals | Cobalt-zinc-cadmium resistance | DNA-binding heavy metal response regulator |  | *.peg.1085 |
|  |  | Cobalt-zinc-cadmium resistance protein CzcD |  | *.peg.3658, *.peg.6357 |
|  |  | Transcriptional regulator, MerR family |  | *.peg.333, *.peg.1463, *.peg.3013, *.peg.3327, *.peg.3466, *.peg.4063, *.peg.4706 |
|  | Copper homeostasis/ tolerance | Multicopper oxidase |  | *.peg.2925 |
|  |  | Copper resistance protein CopC |  | *.peg.144, *.peg.3859 |
|  |  | Multidrug resistance transporter, Bcr/CflA family |  | *.peg.5272, *.peg.6201 |
|  |  | Cu(I)-responsive transcriptional regulator |  | *.peg.1320 |
|  |  | Copper resistance protein D |  | *.peg.721, *.peg.2915, *.peg.3858 |
|  |  | Copper resistance protein CopD |  | *.peg.144 |
|  |  | Copper chaperone |  | *.peg.6172 |
|  |  | Copper-translocating P-type ATPase | 3.6.3.4 | *.peg.6173 |
|  | Arsenic resistance | Arsenical resistance operon repressor |  | *.peg.1251, *.peg.1256, *.peg.2443, *.peg.2450, *.peg.2666 |
|  |  | Arsenical pump-driving ATPase | 3.6.3.16 | *.peg.1166, *.peg.5147, *.peg.5148 |
|  |  | Arsenic efflux pump protein |  | *.peg.1006 |
|  |  | Arsenate reductase | 1.20.4.1 | *.peg.938, *.peg.1254, *.peg.1255, *.peg.2447, *.peg.2448, *.peg.2449 |
|  | Mercury resistance & detoxification | Mercuric ion reductase | 1.16.1.1 | *.peg.1366 |
|  |  | PF00070 family, FAD-dependent NAD(P)-disulphide oxidoreductase |  | *.peg.1094, *.peg.1366, *.peg.4391 |
|  | Uptake of selenate/ selenite | Various polyols ABC transporter, permease component 2 |  | *.peg.5554 |
|  |  | Sulfate and thiosulfate import ATP-binding protein CysA | 3.6.3.25 | *.peg.93, *.peg.2605, *.peg.3773 |
|  |  | DedA protein |  | *.peg.791, *.peg.1993, *.peg.5743 |
|  |  | Various polyols ABC transporter, periplasmic substrate-binding protein |  | *.peg.2499, *.peg.5552 |
|  |  | Various polyols ABC transporter, permease component 1 |  | *.peg.3287, *.peg.5553 |
| VOC´s PGP | Acetoin butanediol metabolism | Alpha-acetolactate decarboxylase | 4.1.1.5 | *.peg.1557, *.peg.3455 |
|  |  | Acetolactate synthase large subunit | 2.2.1.6 | *.peg.2390, *.peg.2528, *.peg.4090 |
|  |  | Acetolactate synthase small subunit | 2.2.1.6 | *.peg.2529 |
|  |  | Acetolactate synthase, catabolic | 2.2.1.6 | *.peg.1558, *.peg.3454 |
|  |  | 2,3-butanediol dehydrogenase, S-alcohol forming, (S)-acetoin-specific | 1.1.1.76 | *.peg.5131 |
| Resistance to antibiotics | Fluoro-quinolone resistance | DNA gyrase subunit B | 5.99.1.3 | *.peg.3605 |
|  |  | DNA gyrase subunit A | 5.99.1.3 | *.peg.3606 |
|  |  | Topoisomerase IV subunit B | 5.99.1.- | *.peg.4703 |
|  |  | Topoisomerase IV subunit A | 5.99.1.- | *.peg.4704 |
|  | Beta-lactamase | Beta-lactamase class C and other penicillin binding proteins |  | *.peg.1196, *.peg.3040, *.peg.4656 |
|  |  | Beta-lactamase | 3.5.2.6 | *.peg.53, *.peg.55, *.peg.5048, *.peg.5503, *.peg.2283 |
|  |  | Metal-dependent hydrolases of the beta-lactamase superfamily III |  | *.peg.807 |
|  | Oxalate catabolism | Oxalyl-CoA decarboxylase | 4.1.1.8 | *.peg.3970 |
|  | 4-hydroxybenzoate degradation | P-hydroxybenzoate hydroxylase | 1.14.13.2 | *.peg.4596 |
| Antibiosis compounds | Clavulanic acid biosynthesis | clavaldehyde dehydrogenase |  | *.peg.4028 |
|  | Chitinase & β-hexosaminidase | Beta-hexosaminidase | 3.2.1.52 | *.peg.2208 |
| Resistance to oxidative stress | Peroxidase | Ferrous iron transport peroxidase EfeB |  | *.peg.945, *.peg.3648 |
|  |  | Predicted dye-decolorizing peroxidase (DyP), encapsulated subgroup |  | *.peg.5371 |
|  |  | Encapsulating protein for a DyP-type peroxidase or ferritin-like protein oligomers |  | *.peg.5370 |
|  |  | Peroxidase | 1.11.1.7 | *.peg.1498, *.peg.2873, *.peg.5770 |
|  |  | Thiol peroxidase, Tpx-type | 1.11.1.15 | *.peg.5783 |
|  |  | Thiol peroxidase, Bcp-type | 1.11.1.15 | *.peg.766 |
|  | Catalase | Catalase | 1.11.1.6 | *.peg.201, *.peg.1426, *.peg.1427, *.peg.1496, *.peg.1498, *.peg.3627, *.peg.4697, *.peg.5191, *.peg.5282, *.peg.5770 |
|  | Superoxide dismutase | Superoxide dismutase [Mn/Fe] | 1.15.1.1 | *.peg.2669 |
|  |  | Superoxide dismutase [Cu-Zn] precursor | 1.15.1.1 | *.peg.1906 |
|  |  | FIG017534: hypothetical protein |  | *.peg.3867 |
|  | Hydroperoxide reductase | Alkyl hydroperoxide reductase subunit C-like protein |  | *.peg.1212, *.peg.3209 |
|  |  | Organic hydroperoxide resistance protein |  | *.peg.4033, *.peg.5189 |
|  |  | Organic hydroperoxide resistance transcriptional regulator |  | *.peg.4034, *.peg.4577 |
|  | Glutathione-mediated detoxification | Lactoylglutathione lyase | 4.4.1.5 | *.peg.5440 |
|  |  | Hydroxyacylglutathione hydrolase | 3.1.2.6 | *.peg.79, *.peg.2973 |
|  | Mycothiol | NADPH-dependent mycothiol reductase Mtr |  | *.peg.2670 |
|  |  | Formaldehyde dehydrogenase MscR, NAD/mycothiol-dependent | 1.2.1.66 | *.peg.1193 |
|  |  | Putative hydrolase in cluster with formaldehyde/S-nitrosomycothiol reductase MscR |  | *.peg.1192 |
|  |  | S-nitrosomycothiol reductase MscR |  | *.peg.1193 |
|  |  | Mycothiol S-conjugate amidase Mca |  | *.peg.6395 |
|  |  | L-cysteine:1D-myo-inosityl 2-amino-2-deoxy-alpha-D-glucopyranoside ligase MshC |  | *.peg.1106 |
|  |  | Glycosyltransferase MshA involved in mycothiol biosynthesis | 2.4.1.- | *.peg.1830 |
|  |  | Uncharacterized protein Rv0487/MT0505 clustered with mycothiol biosynthesis gene |  | *.peg.1829 |
|  |  | Acetyl-CoA:Cys-GlcN-Ins acetyltransferase, mycothiol synthase MshD |  | *.peg.5394 |
|  |  | N-acetyl-1-D-myo-inosityl-2-amino-2-deoxy-alpha-D-glucopyranoside deacetylase MshB |  | *.peg.6268 |
| Heat/ cold shock | Heat shock protein/ chaperone | Hypothetical radical SAM family enzyme in heat shock gene cluster, similarity with CPO of BS HemN-type |  | *.peg.101 |
|  |  | HspR, transcriptional repressor of DnaK operon |  | *.peg.2011 |
|  |  | Heat-inducible transcription repressor HrcA |  | *.peg.103 |
|  |  | Chaperone protein DnaK |  | *.peg.2014 |
|  |  | Chaperone protein DnaJ |  | *.peg.104, *.peg.2012 |
|  |  | Ribosomal RNA small subunit methyltransferase E | 2.1.1.- | *.peg.105 |
|  |  | tmRNA-binding protein SmpB |  | *.peg.4601 |
|  |  | Heat shock protein GrpE |  | *.peg.2013 |
|  |  | Translation elongation factor LepA |  | *.peg.74 |
|  |  | Ribosome-associated heat shock protein implicated in the recycling of the 50S subunit (S4 paralog) |  | *.peg.5804 |
|  |  | Nucleoside 5-triphosphatase RdgB (dHAPTP, dITP, XTP-specific) | 3.6.1.15 | *.peg.805 |
|  |  | Ribonuclease PH | 2.7.7.56 | *.peg.806 |
|  |  | rRNA small subunit methyltransferase I |  | *.peg.918 |
|  |  | Heat shock protein 60 family co-chaperone GroES |  | *.peg.4066 |
|  |  | Heat shock protein 60 family chaperone GroEL |  | *.peg.1352, *.peg.1872, *.peg.4065 |
|  | Cold shock protein | Cold shock protein CspA |  | *.peg.2977 |
|  |  | Cold shock protein CspC |  | *.peg.5451 |
| Salt tolerance | Choline/ betaine uptake & biosynthesis | Sarcosine oxidase gamma subunit | 1.5.3.1 | *.peg.3269 |
|  |  | Sarcosine oxidase alpha subunit | 1.5.3.1 | *.peg.2371, *.peg.3268 |
|  |  | Sarcosine oxidase beta subunit | 1.5.3.1 | *.peg.1930, *.peg.2431, *.peg.3266, *.peg.3329 |
|  |  | High-affinity choline uptake protein BetT |  | *.peg.3260 |
|  |  | Choline-sulfatase | 3.1.6.6 | *.peg.2695, *.peg.6370 |
|  |  | Betaine aldehyde dehydrogenase | 1.2.1.8 | *.peg.3980, *.peg.5060 |
|  |  | Glycine betaine ABC transport system permease protein |  | *.peg.4004, *.peg.5294 |
|  |  | L-proline glycine betaine ABC transport system permease protein ProV | 3.A.1.12.1 | *.peg.4006, *.peg.5296 |
|  |  | Glycine betaine transporter OpuD |  | *.peg.848, *.peg.2419 |
|  |  | Sarcosine oxidase delta subunit | 1.5.3.1 | *.peg.3267 |
|  |  | L-proline glycine betaine binding ABC transporter protein ProX | 3.A.1.12.1 | *.peg.4003, *.peg.5297 |
|  |  | Choline dehydrogenase | 1.1.99.1 | *.peg.433, *.peg.830, *.peg.1249, *.peg.3233, *.peg.3299, *.peg.4201, *.peg.5058, *.peg.5867, *.peg.5961, *.peg.6160 |
|  |  | L-proline glycine betaine ABC transport system permease protein ProW | 3.A.1.12.1 | *.peg.4005, *.peg.5295 |
|  | K+ /Na+ transport | Osmosensitive K+ channel histidine kinase KdpD | 2.7.3.- | *.peg.959, *.peg.1009, *.peg.5279, *.peg.5420, *.peg.5633 |
|  |  | Potassium channel protein |  | *.peg.4763, *.peg.5506 |
|  |  | Potassium efflux system KefA protein |  | *.peg.893, *.peg.2762, *.peg.4605 |
|  |  | Potassium voltage-gated channel subfamily KQT |  | *.peg.6064 |
|  |  | Potassium-transporting ATPase A chain | 3.6.3.12 | *.peg.962 |
|  |  | Potassium-transporting ATPase B chain | 3.6.3.12 | *.peg.961 |
|  |  | Potassium-transporting ATPase C chain | 3.6.3.12 | *.peg.960 |
|  |  | Trk system potassium uptake protein TrkA |  | *.peg.2338, *.peg.2804, *.peg.2805, *.peg.2338, *.peg.2804, *.peg.2805 |
|  |  | Kup system potassium uptake protein |  | *.peg.2930 |
|  |  | Large-conductance mechanosensitive channel |  | *.peg.5638 |
|  |  | Na(+) H(+) antiporter subunit A |  | *.peg.1893 |
|  |  | Na(+) H(+) antiporter subunit B |  | *.peg.1893 |
|  |  | Na(+) H(+) antiporter subunit C |  | *.peg.1894 |
|  |  | Na(+) H(+) antiporter subunit D |  | *.peg.1895 |
|  |  | Na(+) H(+) antiporter subunit E |  | *.peg.1896 |
|  |  | Na(+) H(+) antiporter subunit F |  | *.peg.1897 |
|  |  | Na(+) H(+) antiporter subunit G |  | *.peg.1898 |
|  |  | Na+/H+ antiporter NhaA type |  | *.peg.2811 |
|  |  | Na+/H+ antiporter NhaD type |  | *.peg.2809 |
| Protection from UV radiation and oxidative stress | Mycosporine synthesis | D-alanine--D-alanine ligase, MysD | 6.3.2.4 | *.peg.2331 |
|  |  | O-methyltransferase MysB |  | *.peg.2333 |
|  |  | ATP-grasp ligase forming mycosporine-glycine, MysC |  | *.peg.2332 |
|  |  | Demethyl 4-deoxygadusol synthase MysA |  | *.peg.2334 |
| **Myco. P9-22 (fig\|6666666.353106)** | | |  |  |
| Category | Subsytem | Role/ Protein | EC number | Protein encoding genes |
| Siderophore / Fe uptake | Siderohore receptors/ transport | ABC transporter (iron.B12.siderophore.hemin) , permease component |  | *.peg.5895 |
|  |  | ABC transporter (iron.B12.siderophore.hemin) , ATP-binding component |  | *.peg.5894 |
|  |  | ABC transporter (iron.B12.siderophore.hemin) , periplasmic substrate-binding component |  | *.peg.5896 |
|  |  | Ferric siderophore transport system, periplasmic binding protein TonB |  | *.peg.822 |
|  |  | ABC-type Fe3+-siderophore transport system, permease 2 component |  | *.peg.3952, *.peg.5814 |
|  | Ferrous iron transporter | Ferrous iron transport peroxidase EfeB |  | *.peg.6 |
|  |  | Ferrous iron transport periplasmic protein EfeO, contains peptidase-M75 domain and (frequently) cupredoxin-like domain |  | *.peg.7 |
| Phosphate solubilization | Phosphatase | Exopolyphosphatase | 3.6.1.11 | *.peg.12, *.peg.3481 |
|  |  | secreted alkaline phosphatase |  | *.peg.3668 |
|  |  | Inorganic pyrophosphatase | 3.6.1.1 | *.peg.3684 |
|  |  | Alkaline phosphatase | 3.1.3.1 | *.peg.352, *.peg.675 |
| Phosphate uptake and transport | Low-affinity inorganic phosphate trans. system | Probable low-affinity inorganic phosphate transporter |  | *.peg.3419 |
|  | High-affinity phosphate transport system | Phosphate regulon transcriptional regulatory protein PhoB (SphR) |  | *.peg.3436, *.peg.3483, *.peg.3987 |
|  |  | Phosphate transport system permease protein PstC | 3.A.1.7.1 | *.peg.3990 |
|  |  | Phosphate transport ATP-binding protein PstB | 3.A.1.7.1 | *.peg.3992 |
|  |  | Phosphate transport system permease protein PstA | 3.A.1.7.1 | *.peg.3991 |
|  |  | Phosphate transport system regulatory protein PhoU |  | *.peg.3426, *.peg.3995 |
|  |  | Phosphate ABC transporter, periplasmic phosphate-binding protein PstS | 3.A.1.7.1 | *.peg.3989, *.peg.5428 |
|  |  | Polyphosphate kinase | 2.7.4.1 | *.peg.6403 |
|  |  | Phosphate regulon sensor protein PhoR (SphS) | 2.7.13.3 | *.peg.3484 |
|  | Phosphonate ABC transporter | Phosphonate ABC transporter permease protein phnE | 3.A.1.9.1 | *.peg.3914 |
|  |  | Phosphonate ABC transporter ATP-binding protein | 3.A.1.9.1 | *.peg.3915 |
|  |  | Phosphonate ABC transporter phosphate-binding periplasmic component | 3.A.1.9.1 | *.peg.3916 |
| Plant hormone | Auxin biosynthesis | Tryptophan synthase alpha chain | 4.2.1.20 | *.peg.4347 |
|  |  | Anthranilate phosphoribosyltransferase | 2.4.2.18 | *.peg.2315 |
|  |  | Tryptophan synthase beta chain | 4.2.1.20 | *.peg.4348 |
|  |  | Monoamine oxidase (1.4.3.4) |  | *.peg.321, *.peg.2566, *.peg.4154, *.peg.5646 |
| Polyamine | Putrescine/ spermidine synthesis | Putrescine transport ATP-binding protein PotG | 3.A.1.11.2 | *.peg.340 |
|  |  | Putrescine transport system permease protein PotH | 3.A.1.11.2 | *.peg.338 |
|  |  | Putrescine transport ATP-binding protein PotA | 3.A.1.11.1 | *.peg.2461 |
|  |  | Spermidine Putrescine ABC transporter permease component potC | 3.A.1.11.1 | *.peg.2462 |
|  |  | Putrescine transport system permease protein PotI | 3.A.1.11.2 | *.peg.337 |
|  |  | Putrescine ABC transporter putrescine-binding protein PotF | 3.A.1.11.2 | *.peg.339 |
|  |  | ABC transporter, periplasmic spermidine putrescine-binding protein PotD | 3.A.1.11.1 | *.peg.2463 |
|  | Putrescine/ spermidine transport | Agmatine deiminase | 3.5.3.12 | *.peg.944 |
|  |  | Arginine decarboxylase | 4.1.1.19 | *.peg.644 |
|  |  | Ornithine decarboxylase | 4.1.1.17 | *.peg.644 |
|  |  | Spermidine synthase | 2.5.1.16 | *.peg.6449 |
|  |  | Agmatinase | 3.5.3.11 | *.peg.1989, *.peg.3402 |
|  |  | N-carbamoylputrescine amidase | 3.5.1.53 | *.peg.943 |
|  |  | Carboxynorspermidine dehydrogenase, putative | 1.1.1.- | *.peg.372 |
|  |  | Carbamate kinase | 2.7.2.2 | *.peg.3097 |
| Resistance to heavy metals | Cobalt-zinc-cadmium resistance | DNA-binding heavy metal response regulator |  | *.peg.274 |
|  |  | Cobalt-zinc-cadmium resistance protein CzcD |  | *.peg.5320 |
|  |  | Transcriptional regulator, MerR family |  | *.peg.363, *.peg.536, *.peg.731, *.peg.1615, *.peg.3445, *.peg.4389, *.peg.4864, *.peg.5343, *.peg.5435, *.peg.5600 |
|  | Copper homeostasis/ tolerance | Multicopper oxidase |  | *.peg.974 |
|  |  | Copper-translocating P-type ATPase | 3.6.3.4 | *.peg.16, *.peg.601, *.peg.603, *.peg.4639, *.peg.6186 |
|  |  | Copper resistance protein CopC |  | *.peg.1077 |
|  |  | Copper chaperone |  | *.peg.599 |
|  |  | Multidrug resistance transporter, Bcr/CflA family |  | *.peg.4776, *.peg.6210 |
|  |  | Copper resistance protein D |  | *.peg.1076, *.peg.1626 |
|  | Arsenic resistance | Arsenical resistance operon repressor |  | *.peg.609, *.peg.611, *.peg.4895 |
|  |  | Arsenical pump-driving ATPase | 3.6.3.16 | *.peg.2323, *.peg.3622, *.peg.3623 |
|  |  | Arsenic efflux pump protein |  | *.peg.3730 |
|  |  | Arsenate reductase | 1.20.4.1 | *.peg.606, *.peg.607, *.peg.608, *.peg.4336 |
|  | Mercury resistance & detoxification | PF00070 family, FAD-dependent NAD(P)-disulphide oxidoreductase |  | *.peg.256 |
|  | Chromium compounds resistance | Chromate resistance protein ChrB |  | *.peg.5839 |
|  |  | Chromate transport protein ChrA |  | *.peg.5837 |
|  | Uptake of selenate/ selenite | Sulfate and thiosulfate import ATP-binding protein CysA | 3.6.3.25 | *.peg.1023, *.peg.1919, *.peg.6322 |
|  |  | DedA protein |  | *.peg.1398, *.peg.5326 |
|  |  | Various polyols ABC transporter, permease component 1 |  | *.peg.4467 |
| VOC´s PGP | Acetoin butanediol metabolism | Dihydrolipoamide dehydrogenase of acetoin dehydrogenase | 1.8.1.4 | *.peg.55 |
|  |  | Acetolactate synthase large subunit | 2.2.1.6 | *.peg.663, *.peg.1988, *.peg.3147, *.peg.6426 |
|  |  | Acetolactate synthase small subunit | 2.2.1.6 | *.peg.6425 |
|  |  | Acetoin dehydrogenase E1 component beta-subunit | 1.2.4.- | *.peg.2186 |
|  |  | Acetoin dehydrogenase E1 component alpha-subunit | 1.2.4.- | *.peg.2185 |
|  |  | 2,3-butanediol dehydrogenase, S-alcohol forming, (S)-acetoin-specific | 1.1.1.76 | *.peg.961 |
| Resistance to antibiotics | Fluoro-quinolone resistance | DNA gyrase subunit B | 5.99.1.3 | *.peg.5947 |
|  |  | DNA gyrase subunit A | 5.99.1.3 | *.peg.5948 |
|  |  | Topoisomerase IV subunit B | 5.99.1.- | *.peg.5799 |
|  |  | Topoisomerase IV subunit A | 5.99.1.- | *.peg.5800 |
|  | Beta-lactamase | Beta-lactamase class C and other penicillin binding proteins |  | *.peg.2021, *.peg.4200, *.peg.4537 |
|  |  | Beta-lactamase | 3.5.2.6 | *.peg.3711, *.peg.4165, *.peg.4841, *.peg.5861, *.peg.992, *.peg.4147 |
|  |  | Metal-dependent hydrolases of the beta-lactamase superfamily III |  | *.peg.1424 |
| Antibiosis compounds | Chitinase & β-hexosaminidase | Beta-hexosaminidase | 3.2.1.52 | *.peg.899 |
| Resistance to oxidative stress | Peroxidase | Ferrous iron transport peroxidase EfeB |  | *.peg.6 |
|  |  | Predicted dye-decolorizing peroxidase (DyP), encapsulated subgroup |  | *.peg.3965 |
|  |  | Encapsulating protein for a DyP-type peroxidase or ferritin-like protein oligomers |  | *.peg.3964 |
|  |  | Peroxidase | 1.11.1.7 | *.peg.2491, *.peg.5979, *.peg.6436 |
|  |  | Thiol peroxidase, Tpx-type | 1.11.1.15 | *.peg.2529 |
|  |  | Thiol peroxidase, Bcp-type | 1.11.1.15 | *.peg.1574 |
|  | Catalase | Catalase | 1.11.1.6 | *.peg.657, *.peg.2491, *.peg.5979 |
|  | Superoxide dismutase | Superoxide dismutase [Mn/Fe] | 1.15.1.1 | *.peg.1084 |
|  |  | Superoxide dismutase [Mn] | 1.15.1.1 | *.peg.6316 |
|  |  | Superoxide dismutase [Cu-Zn] precursor | 1.15.1.1 | *.peg.3559 |
|  |  | FIG017534: hypothetical protein |  | *.peg.1083 |
|  | Hydroperoxide reductase | Alkyl hydroperoxide reductase subunit C-like protein |  | *.peg.2041, *.peg.4352 |
|  |  | Organic hydroperoxide resistance protein |  | *.peg.947 |
|  |  | Organic hydroperoxide resistance transcriptional regulator |  | *.peg.948, *.peg.4079 |
|  |  | Alkyl hydroperoxide reductase protein C | 1.6.4.- | *.peg.6414 |
|  |  | Alkylhydroperoxidase protein D |  | *.peg.6415 |
|  | Glutathione-mediated detoxification | Glutathione S-transferase domain protein |  | *.peg.4742 |
|  |  | Lactoylglutathione lyase | 4.4.1.5 | *.peg.610, *.peg.4080, *.peg.6508 |
|  |  | Hydroxyacylglutathione hydrolase | 3.1.2.6 | *.peg.3105, *.peg.3106, *.peg.4613 |
|  | Mycothiol | NADPH-dependent mycothiol reductase Mtr |  | *.peg.4894 |
|  |  | Formaldehyde dehydrogenase MscR, NAD/mycothiol-dependent | 1.2.1.66 | *.peg.2020 |
|  |  | Putative hydrolase in cluster with formaldehyde/S-nitrosomycothiol reductase MscR |  | *.peg.2019 |
|  |  | S-nitrosomycothiol reductase MscR |  | *.peg.2020 |
|  |  | Mycothiol S-conjugate amidase Mca |  | *.peg.114 |
|  |  | L-cysteine:1D-myo-inosityl 2-amino-2-deoxy-alpha-D-glucopyranoside ligase MshC |  | *.peg.241 |
|  |  | Glycosyltransferase MshA involved in mycothiol biosynthesis | 2.4.1.- | *.peg.3487 |
|  |  | Uncharacterized protein Rv0487/MT0505 clustered with mycothiol biosynthesis gene |  | *.peg.3486 |
|  |  | Acetyl-CoA:Cys-GlcN-Ins acetyltransferase, mycothiol synthase MshD |  | *.peg.3988 |
|  |  | N-acetyl-1-D-myo-inosityl-2-amino-2-deoxy-alpha-D-glucopyranoside deacetylase MshB |  | *.peg.6280 |
| Heat/ cold shock | Heat shock protein/ chaperone | Hypothetical radical SAM family enzyme in heat shock gene cluster, similarity with CPO of BS HemN-type |  | *.peg.1927 |
|  |  | HspR, transcriptional repressor of DnaK operon |  | *.peg.432, *.peg.5344 |
|  |  | Heat-inducible transcription repressor HrcA |  | *.peg.1942 |
|  |  | Chaperone protein DnaK |  | *.peg.5347 |
|  |  | Chaperone protein DnaJ |  | *.peg.1943, *.peg.5345 |
|  |  | Ribosomal RNA small subunit methyltransferase E | 2.1.1.- | *.peg.1944 |
|  |  | tmRNA-binding protein SmpB |  | *.peg.5128 |
|  |  | Heat shock protein GrpE |  | *.peg.5346 |
|  |  | Translation elongation factor LepA |  | *.peg.1890 |
|  |  | Ribosome-associated heat shock protein implicated in the recycling of the 50S subunit (S4 paralog) |  | *.peg.2511 |
|  |  | Nucleoside 5-triphosphatase RdgB (dHAPTP, dITP, XTP-specific) | 3.6.1.15 | *.peg.1426 |
|  |  | Ribonuclease PH | 2.7.7.56 | *.peg.1425 |
|  |  | rRNA small subunit methyltransferase I |  | *.peg.4319 |
|  |  | Heat shock protein 60 family co-chaperone GroES |  | *.peg.3005 |
|  |  | Heat shock protein 60 family chaperone GroEL |  | *.peg.3004, *.peg.3536 |
|  | Cold shock protein | Cold shock protein CspA |  | *.peg.2516, *.peg.3648 |
|  |  | Cold shock protein CspC |  | *.peg.4091 |
| Salt tolerance | Choline/ betaine uptake & biosynthesis | Sarcosine oxidase alpha subunit | 1.5.3.1 | *.peg.6368 |
|  |  | Sarcosine oxidase beta subunit | 1.5.3.1 | *.peg.537 |
|  |  | High-affinity choline uptake protein BetT |  | *.peg.2547 |
|  |  | Choline-sulfatase | 3.1.6.6 | *.peg.4583, *.peg.5053 |
|  |  | Betaine aldehyde dehydrogenase | 1.2.1.8 | *.peg.2908, *.peg.3179 |
|  |  | Glycine betaine ABC transport system permease protein |  | *.peg.457 |
|  |  | L-proline glycine betaine ABC transport system permease protein ProV | 3.A.1.12.1 | *.peg.455 |
|  |  | Glycine betaine transporter OpuD |  | *.peg.496, *.peg.2716 |
|  |  | L-proline glycine betaine binding ABC transporter protein ProX | 3.A.1.12.1 | *.peg.454 |
|  |  | Choline dehydrogenase | 1.1.99.1 | *.peg.379, *.peg.406, *.peg.1129, *.peg.2140, *.peg.3209 |
|  |  | L-proline glycine betaine ABC transport system permease protein ProW | 3.A.1.12.1 | *.peg.456 |
|  | Ectoine biosynthesis | Diaminobutyrate-pyruvate aminotransferase | 2.6.1.46 | *.peg.276 |
|  |  | Ectoine hydroxylase | 1.17.-.- | *.peg.278 |
|  |  | L-ectoine synthase | 4.2.1.- | *.peg.277 |
|  |  | L-2,4-diaminobutyric acid acetyltransferase | 2.3.1.- | *.peg.275 |
|  | K+ /Na+ transport | Na(+) H(+) antiporter subunit A |  | *.peg.3551 |
|  |  | Na(+) H(+) antiporter subunit B |  | *.peg.3551 |
|  |  | Na(+) H(+) antiporter subunit C |  | *.peg.3552 |
|  |  | Na(+) H(+) antiporter subunit D |  | *.peg.3553 |
|  |  | Na(+) H(+) antiporter subunit E |  | *.peg.3554 |
|  |  | Na(+) H(+) antiporter subunit F |  | *.peg.3555 |
|  |  | Na(+) H(+) antiporter subunit G |  | *.peg.3556 |
|  |  | Na+/H+ antiporter NhaA type |  | *.peg.4723, *.peg.5327 |
|  |  | Sodium-dependent phosphate transporter |  | *.peg.5429 |
|  |  | Osmosensitive K+ channel histidine kinase KdpD | 2.7.3.- | *.peg.26, *.peg.27, *.peg.28, *.peg.596, *.peg.3792, *.peg.4277 |
|  |  | Potassium channel protein |  | *.peg.2661 |
|  |  | Potassium efflux system KefA protein |  | *.peg.805, *.peg.5132 |
|  |  | Potassium-transporting ATPase A chain | 3.6.3.12 | *.peg.33 |
|  |  | Potassium-transporting ATPase B chain | 3.6.3.12 | *.peg.32 |
|  |  | Potassium-transporting ATPase C chain | 3.6.3.12 | *.peg.31 |
|  |  | Trk system potassium uptake protein TrkA |  | *.peg.4726, *.peg.4727, *.peg.5710 |
|  |  | Glutathione-regulated potassium-efflux system protein KefB |  | *.peg.3110 |
|  |  | Kup system potassium uptake protein |  | *.peg.5210 |
|  |  | Large-conductance mechanosensitive channel |  | *.peg.4285 |
| **Myco. P9-64 (fig\|6666666.353107)** | | |  |  |
| Category | Subsytem | Role/ Protein | EC number | Protein encoding genes |
| Siderophore / Fe uptake | Siderohore receptors/ transport | ABC transporter (iron.B12.siderophore.hemin) , permease component |  | *.peg.1286 |
|  |  | ABC transporter (iron.B12.siderophore.hemin) , ATP-binding component |  | *.peg.1287 |
|  |  | ABC transporter (iron.B12.siderophore.hemin) , periplasmic substrate-binding component |  | *.peg.1285 |
|  | Ferrous iron transporter | Ferrous iron transport peroxidase EfeB |  | *.peg.6398 |
|  |  | Ferrous iron transport permease EfeU |  | *.peg.6400 |
|  |  | Ferrous iron transport periplasmic protein EfeO, contains peptidase-M75 domain and (frequently) cupredoxin-like domain |  | *.peg.6399 |
| Phosphate solubilization | Phosphatase | Exopolyphosphatase | 3.6.1.11 | *.peg.2194, *.peg.6405 |
|  |  | secreted alkaline phosphatase |  | *.peg.2358 |
|  |  | Inorganic pyrophosphatase | 3.6.1.1 | *.peg.3285 |
|  |  | Alkaline phosphatase | 3.1.3.1 | *.peg.2364, *.peg.5889 |
|  | Pyrrolo-quinoline Quinone biosynthesis | Coenzyme PQQ synthesis protein D |  | *.peg.5083 |
|  |  | Coenzyme PQQ synthesis protein A |  | *.peg.5080 |
|  |  | Coenzyme PQQ synthesis protein B |  | *.peg.5081 |
|  |  | Coenzyme PQQ synthesis protein C |  | *.peg.5082 |
|  |  | Coenzyme PQQ synthesis protein E |  | *.peg.5084 |
| Phosphate uptake and transport | Low-affinity inorganic phosphate trans. system | Probable low-affinity inorganic phosphate transporter |  | *.peg.2251 |
|  | High-affinity phosphate transport system | Phosphate regulon transcriptional regulatory protein PhoB (SphR) |  | *.peg.1725, *.peg.2192, *.peg.2237, *.peg.4890, *.peg.4896, *.peg.5778 |
|  |  | Phosphate transport system permease protein PstC | 3.A.1.7.1 | *.peg.4893 |
|  |  | Phosphate transport ATP-binding protein PstB | 3.A.1.7.1 | *.peg.4895 |
|  |  | Phosphate transport system permease protein PstA | 3.A.1.7.1 | *.peg.4894 |
|  |  | Phosphate transport system regulatory protein PhoU |  | *.peg.4634, *.peg.4899 |
|  |  | Phosphate ABC transporter, periplasmic phosphate-binding protein PstS | 3.A.1.7.1 | *.peg.4892 |
|  |  | Polyphosphate kinase | 2.7.4.1 | *.peg.6022 |
|  |  | Phosphate regulon sensor protein PhoR (SphS) | 2.7.13.3 | *.peg.2191 |
| Plant hormone | Auxin biosynthesis | Tryptophan synthase alpha chain | 4.2.1.20 | *.peg.3554 |
|  |  | Aromatic-L-amino-acid decarboxylase | 4.1.1.28 | *.peg.1836 |
|  |  | Anthranilate phosphoribosyltransferase | 2.4.2.18 | *.peg.4712 |
|  |  | Tryptophan synthase beta chain | 4.2.1.20 | *.peg.3555 |
|  |  | Monoamine oxidase | 1.4.3.4 | *.peg.4043, *.peg.4432, *.peg.5068, *.peg.5370, *.peg.5522, *.peg.6137 |
| Polyamine | Putrescine/ spermidine synthesis | Putrescine transport ATP-binding protein PotG | 3.A.1.11.2 | *.peg.2365, *.peg.5403 |
|  |  | Putrescine transport system permease protein PotH | 3.A.1.11.2 | *.peg.5401 |
|  |  | Putrescine transport ATP-binding protein PotA | 3.A.1.11.1 | *.peg.191, *.peg.2077, *.peg.2635, *.peg.4244 |
|  |  | Spermidine Putrescine ABC transporter permease component PotB | 3.A.1.11.1 | *.peg.189, *.peg.2074, *.peg.2369, *.peg.4242 |
|  |  | Putrescine transport system permease protein PotI | 3.A.1.11.2 | *.peg.2368, *.peg.5400 |
|  |  | Putrescine ABC transporter putrescine-binding protein PotF | 3.A.1.11.2 | *.peg.4334, *.peg.5402 |
|  |  | ABC transporter, periplasmic spermidine putrescine-binding protein PotD | 3.A.1.11.1 | *.peg.190, *.peg.2075, *.peg.2367, *.peg.2377, *.peg.4243 |
|  |  | Spermidine Putrescine ABC transporter permease component potC | 3.A.1.11.1 | *.peg.188, *.peg.2073, *.peg.4241 |
|  | Putrescine/ spermidine transport | Agmatine deiminase | 3.5.3.12 | *.peg.197 |
|  |  | Arginine decarboxylase | 4.1.1.19 | *.peg.2326, *.peg.4101, *.peg.5269 |
|  |  | Ornithine decarboxylase | 4.1.1.17 | *.peg.5269 |
|  |  | Spermidine synthase | 2.5.1.16 | *.peg.1299 |
|  |  | Carbamate kinase | 2.7.2.2 | *.peg.316 |
|  |  | Agmatinase | 3.5.3.11 | *.peg.786, *.peg.876, *.peg.1887, *.peg.4584, *.peg.5745 |
| Resistance to heavy metals | Cobalt-zinc-cadmium resistance | DNA-binding heavy metal response regulator |  | *.peg.5313 |
|  |  | Cobalt-zinc-cadmium resistance protein CzcD |  | *.peg.2023, *.peg.5367 |
|  |  | Transcriptional regulator, MerR family |  | *.peg.2488, *.peg.2548, *.peg.3765, *.peg.6154 |
|  | Copper homeostasis/ tolerance | Multicopper oxidase |  | *.peg.2304, *.peg.5975, *.peg.6469 |
|  |  | Copper-translocating P-type ATPase | 3.6.3.4 | *.peg.337, *.peg.459, *.peg.2876, *.peg.6324, *.peg.6444 |
|  |  | Copper resistance protein CopC |  | *.peg.2907 |
|  |  | Copper chaperone |  | *.peg.458 |
|  |  | Multidrug resistance transporter, Bcr/CflA family |  | *.peg.508 |
|  |  | Copper resistance protein D |  | *.peg.2906, *.peg.4327, *.peg.6445 |
|  | Arsenic resistance | Arsenical resistance operon repressor |  | *.peg.2803, *.peg.4910, *.peg.6181 |
|  |  | Arsenical pump-driving ATPase | 3.6.3.16 | *.peg.3222, *.peg.3223, *.peg.4722 |
|  |  | Arsenic efflux pump protein |  | *.peg.3334 |
|  |  | Arsenate reductase | 1.20.4.1 | *.peg.4912, *.peg.4913, *.peg.6391 |
|  | Mercury resistance & detoxification | PF00070 family, FAD-dependent NAD(P)-disulphide oxidoreductase |  | *.peg.549, *.peg.745, *.peg.2524, *.peg.5913, *.peg.6795 |
|  |  | Mercuric ion reductase | 1.16.1.1 | *.peg.549, *.peg.5913 |
|  | Resistance to chromium compounds | Chromate transport protein ChrA |  | *.peg.6747 |
|  | Uptake of selenate/ selenite | Various polyols ABC transporter, permease component 2 |  | *.peg.5194 |
|  |  | Sulfate and thiosulfate import ATP-binding protein CysA | 3.6.3.25 | *.peg.2977, *.peg.4499, *.peg.6105 |
|  |  | DedA protein |  | *.peg.2014, *.peg.3266, *.peg.3906, *.peg.5666 |
|  |  | Various polyols ABC transporter, periplasmic substrate-binding protein |  | *.peg.5192, *.peg.5957 |
|  |  | Various polyols ABC transporter, permease component 1 |  | *.peg.1445, *.peg.5193 |
| VOC´s PGP | Acetoin butanediol metabolism | Dihydrolipoamide acetyltransferase component (E2) of acetoin dehydrogenase complex | 2.3.1.- | *.peg.387 |
|  |  | Acetolactate synthase large subunit | 2.2.1.6 | *.peg.706, *.peg.781, *.peg.1753, *.peg.4583, *.peg.5846, *.peg.5990 |
|  |  | Acetolactate synthase small subunit | 2.2.1.6 | *.peg.5991 |
|  |  | Acetoin dehydrogenase E1 component beta-subunit | 1.2.4.- | *.peg.386 |
|  |  | Acetoin dehydrogenase E1 component alpha-subunit | 1.2.4.- | *.peg.385 |
|  |  | 2,3-butanediol dehydrogenase, S-alcohol forming, (S)-acetoin-specific | 1.1.1.76 | *.peg.463, *.peg.1905 |
| Resistance to antibiotics | Fluoro-quinolone resistance | DNA gyrase subunit B | 5.99.1.3 | *.peg.89 |
|  |  | DNA gyrase subunit A | 5.99.1.3 | *.peg.88 |
|  |  | Topoisomerase IV subunit B | 5.99.1.- | *.peg.175 |
|  |  | Topoisomerase IV subunit A | 5.99.1.- | *.peg.174 |
|  | Beta-lactamase | Beta-lactamase class C and other penicillin binding proteins |  | *.peg.3283, *.peg.3726, *.peg.4615, *.peg.5998 |
|  |  | Beta-lactamase | 3.5.2.6 | *.peg.3314, *.peg.5106, *.peg.6232, *.peg.2778, *.peg.5470 |
|  |  | Metal-dependent hydrolases of the beta-lactamase superfamily III |  | *.peg.4199 |
|  | Oxalate catabolism | Oxalyl-CoA decarboxylase | 4.1.1.8 | *.peg.1086 |
| Antibiosis compounds | Chitinase & β-hexosaminidase | Beta-hexosaminidase | 3.2.1.52 | *.peg.1642 |
| Resistance to oxidative stress | Peroxidase | Ferrous iron transport peroxidase EfeB |  | *.peg.6398 |
|  |  | Predicted dye-decolorizing peroxidase (DyP), encapsulated subgroup |  | *.peg.4861 |
|  |  | Encapsulating protein for a DyP-type peroxidase or ferritin-like protein oligomers |  | *.peg.4860 |
|  |  | Peroxidase | 1.11.1.7 | *.peg.4283, *.peg.4807, *.peg.5076 |
|  |  | Thiol peroxidase, Tpx-type | 1.11.1.15 | *.peg.5626 |
|  |  | Thiol peroxidase, Bcp-type | 1.11.1.15 | *.peg.4250 |
|  | Catalase | Catalase | 1.11.1.6 | *.peg.1073, *.peg.1531, *.peg.3169, *.peg.4807, *.peg.5076, *.peg.6809 |
|  | Superoxide dismutase | Superoxide dismutase [Mn/Fe] | 1.15.1.1 | *.peg.3887 |
|  |  | Superoxide dismutase [Cu-Zn] precursor | 1.15.1.1 | *.peg.2110 |
|  |  | FIG037441: Conserved transmembrane protein |  | *.peg.2915 |
|  |  | FIG017534: hypothetical protein |  | *.peg.2916 |
|  | Hydroperoxide reductase | Alkyl hydroperoxide reductase subunit C-like protein |  | *.peg.1300, *.peg.3560, *.peg.4631 |
|  |  | Organic hydroperoxide resistance protein |  | *.peg.1017, *.peg.3171 |
|  |  | Organic hydroperoxide resistance transcriptional regulator |  | *.peg.1018, *.peg.2691 |
|  | Glutathione-mediated detoxification | Lactoylglutathione lyase | 4.4.1.5 | *.peg.4911, *.peg.4999 |
|  |  | Hydroxyacylglutathione hydrolase | 3.1.2.6 | *.peg.3810 |
|  | Mycothiol | NADPH-dependent mycothiol reductase Mtr |  | *.peg.6183 |
|  |  | Formaldehyde dehydrogenase MscR, NAD/mycothiol-dependent | 1.2.1.66 | *.peg.1068, *.peg.3945, *.peg.4614 |
|  |  | Putative hydrolase in cluster with formaldehyde/S-nitrosomycothiol reductase MscR |  | *.peg.3944, *.peg.4613 |
|  |  | S-nitrosomycothiol reductase MscR |  | *.peg.1068, *.peg.3945, *.peg.4614 |
|  |  | Mycothiol S-conjugate amidase Mca |  | *.peg.6536 |
|  |  | L-cysteine:1D-myo-inosityl 2-amino-2-deoxy-alpha-D-glucopyranoside ligase MshC |  | *.peg.2514 |
|  |  | Glycosyltransferase MshA involved in mycothiol biosynthesis | 2.4.1.- | *.peg.2188 |
|  |  | Uncharacterized protein Rv0487/MT0505 clustered with mycothiol biosynthesis gene |  | *.peg.2189 |
|  |  | Acetyl-CoA:Cys-GlcN-Ins acetyltransferase, mycothiol synthase MshD |  | *.peg.4891 |
|  |  | N-acetyl-1-D-myo-inosityl-2-amino-2-deoxy-alpha-D-glucopyranoside deacetylase MshB |  | *.peg.6661 |
| Heat/ cold shock | Heat shock protein/ chaperone | Hypothetical radical SAM family enzyme in heat shock gene cluster, similarity with CPO of BS HemN-type |  | *.peg.4503 |
|  |  | HspR, transcriptional repressor of DnaK operon |  | *.peg.1989, *.peg.4577 |
|  |  | Heat-inducible transcription repressor HrcA |  | *.peg.4523 |
|  |  | Chaperone protein DnaK |  | *.peg.1986 |
|  |  | Chaperone protein DnaJ |  | *.peg.1988, *.peg.4524 |
|  |  | Ribosomal RNA small subunit methyltransferase E | 2.1.1.- | *.peg.4525 |
|  |  | tmRNA-binding protein SmpB |  | *.peg.1503 |
|  |  | Heat shock protein GrpE |  | *.peg.1987 |
|  |  | Translation elongation factor LepA |  | *.peg.4475 |
|  |  | Ribosome-associated heat shock protein implicated in the recycling of the 50S subunit (S4 paralog) |  | *.peg.5551 |
|  |  | Nucleoside 5-triphosphatase RdgB (dHAPTP, dITP, XTP-specific) | 3.6.1.15 | *.peg.4201 |
|  |  | Ribonuclease PH | 2.7.7.56 | *.peg.4200 |
|  |  | rRNA small subunit methyltransferase I |  | *.peg.6363 |
|  |  | Heat shock protein 60 family co-chaperone GroES |  | *.peg.1066 |
|  |  | Heat shock protein 60 family chaperone GroEL |  | *.peg.1065, *.peg.2142, *.peg.6046 |
|  | Cold shock protein | Cold shock protein CspA |  | *.peg.3248, *.peg.4595 |
|  |  | Cold shock protein CspC |  | *.peg.5010 |
| Salt tolerance | Choline/ betaine uptake & biosynthesis | Sarcosine oxidase alpha subunit | 1.5.3.1 | *.peg.4311 |
|  |  | Sarcosine oxidase beta subunit | 1.5.3.1 | *.peg.2085 |
|  |  | High-affinity choline uptake protein BetT |  | *.peg.1695 |
|  |  | Choline-sulfatase | 3.1.6.6 | *.peg.4221 |
|  |  | Betaine aldehyde dehydrogenase | 1.2.1.8 | *.peg.952, *.peg.962 |
|  |  | Glycine betaine ABC transport system permease protein |  | *.peg.2734, *.peg.3016 |
|  |  | L-proline glycine betaine ABC transport system permease protein ProV | 3.A.1.12.1 | *.peg.2736, *.peg.3014 |
|  |  | L-proline glycine betaine binding ABC transporter protein ProX | 3.A.1.12.1 | *.peg.2733, *.peg.3013 |
|  |  | Choline dehydrogenase | 1.1.99.1 | *.peg.447, *.peg.657, *.peg.950, *.peg.1168, *.peg.2647, *.peg.4917, *.peg.5481, *.peg.5514, *.peg.6870 |
|  |  | L-proline glycine betaine ABC transport system permease protein ProW | 3.A.1.12.1 | *.peg.2735, *.peg.3015 |
|  | K+ /Na+ transport | Na(+) H(+) antiporter subunit A |  | *.peg.2120 |
|  |  | Na(+) H(+) antiporter subunit B |  | *.peg.2120 |
|  |  | Na(+) H(+) antiporter subunit C |  | *.peg.2119 |
|  |  | Na(+) H(+) antiporter subunit D |  | *.peg.2118 |
|  |  | Na(+) H(+) antiporter subunit E |  | *.peg.2117 |
|  |  | Na(+) H(+) antiporter subunit F |  | *.peg.2116 |
|  |  | Na(+) H(+) antiporter subunit G |  | *.peg.2115 |
|  |  | Na+/H+ antiporter NhaA type |  | *.peg.2013, *.peg.3928 |
|  |  | Na+/H+ antiporter NhaD type |  | *.peg.3931 |
|  |  | Osmosensitive K+ channel histidine kinase KdpD | 2.7.3.- | *.peg.1151, *.peg.1724, *.peg.3370, *.peg.5306, *.peg.6411 |
|  |  | Potassium channel protein |  | *.peg.5113, *.peg.6922 |
|  |  | Potassium efflux system KefA protein |  | *.peg.1499, *.peg.6336 |
|  |  | Potassium voltage-gated channel subfamily KQT |  | *.peg.1267 |
|  |  | Potassium-transporting ATPase A chain | 3.6.3.12 | *.peg.6414, *.peg.6416 |
|  |  | Potassium-transporting ATPase B chain | 3.6.3.12 | *.peg.6413 |
|  |  | Potassium-transporting ATPase C chain | 3.6.3.12 | *.peg.6412 |
|  |  | Trk system potassium uptake protein TrkA |  | *.peg.3201, *.peg.4104, *.peg.4105, *.peg.3201, *.peg.4104, *.peg.4105 |
|  |  | Kup system potassium uptake protein |  | *.peg.2743 |
|  |  | Large-conductance mechanosensitive channel |  | *.peg.6308 |

Suppl. Table 5: Biosynthetic gene clusters (BGCs) of strains P1-5, P1-18, P9-22 and P9-64 for which antiSMASH software annotated a similar known cluster.

| **Strain** | **Cluster Type** | **Length (bp)** | **Most similar known cluster** | **Similarity (% genes)** | **MIBiG BGC-ID** |
| --- | --- | --- | --- | --- | --- |
| *Myco*. P1-5 | T1pks | 47358 | Tetrocarcin A | 8 | BGC0000162_c1 |
|  | T1pks | 46281 | Griseobactin | 11 | BGC0000368_c1 |
|  | T1pks-Nrps | 113379 | Mycobactin | 70 | BGC0001021_c1 |
|  | Cf_putative | 8330 | Kosinostatin | 3 | BGC0001073_c1 |
|  | Cf_saccharide | 37187 | Phosphonoglycans | 5 | BGC0000806_c1 |
|  | Cf_putative | 23083 | Salinomycin | 6 | BGC0000144_c1 |
|  | Cf_saccharide | 24832 | Mycolic acid | 100 | BGC0000870_c1 |
|  | T1pks | 45477 | Lipopolysaccharide | 5 | BGC0000774_c1 |
|  | Bacteriocin | 10797 | Pactamycin | 3 | BGC0000119_c1 |
|  | T3pks | 41106 | Alkylresorcinol | 66 | BGC0000282_c1 |
|  | Terpene | 20920 | Isorenieratene | 71 | BGC0000664_c1 |
|  | Cf_fatty_acid | 21250 | U-68204 | 14 | BGC0001355_c1 |
|  | Cf_putative | 28944 | Galbonolides | 6 | BGC0000065_c1 |
|  | Otherks | 23961 | Azinomycin B | 4 | BGC0000960_c1 |
|  | Cf_putative | 51520 | Thiolutin | 8 | BGC0001193_c1 |
|  | Cf_putative | 11234 | Fengycin | 13 | BGC0001095_c1 |
|  | Cf_putative | 13554 | Phosphonoglycans | 3 | BGC0000806_c1 |
|  | T3pks | 41133 | Alkylresorcinol | 100 | BGC0000282_c1 |
|  | Cf_saccharide | 27041 | Cephamycin C | 10 | BGC0000319_c1 |
|  | Other | 43944 | Tiancimycin | 5 | BGC0001378_c1 |
|  | Cf_putative | 11421 | Thiolactomycin | 100 | BGC0001353_c2 |
|  | T1pks/saccharide/Nrps | 96094 | Glycopeptidolipid | 43 | BGC0000362_c1 |
|  | Cf_putative | 11958 | Arsenopolyketides | 8 | BGC0001283_c1 |
|  | Otherks | 10167 | Kosinostatin | 3 | BGC0001073_c1 |
| *Myco*. P1-18 | Cf_saccharide | 34494 | Phosphonoglycans | 3 | BGC0000806_c1 |
|  | Cf_putative | 13753 | Glycopeptidolipid | 10 | BGC0000365_c1 |
|  | T1pks-Nrps | 55016 | Glycopeptidolipid | 23 | BGC0000365_c1 |
|  | Nrps | 62664 | Cahuitamycins | 20 | BGC0001351_c1 |
|  | Other | 43530 | Sch47554/Sch47555 | 7 | BGC0000268_c1 |
|  | Cf_putative | 12130 | Rishirilide B | 7 | BGC0001179_c1 |
|  | T1pks/saccharide/Nrps | 60993 | Glycopeptidolipid | 12 | BGC0000362_c1 |
|  | Terpene | 21187 | Glycopeptidolipid | 10 | BGC0000364_c1 |
|  | T1pks | 52440 | Ansamitocin | 7 | BGC0000020_c1 |
|  | Cf_putative | 16294 | Akaeolide | 8 | BGC0001199_c1 |
|  | Cf_putative | 18087 | Mycinamicin | 9 | BGC0000102_c1 |
|  | Cf_putative | 25249 | Salinomycin | 6 | BGC0000144_c1 |
|  | Cf_putative | 31311 | Mycolic acid | 100 | BGC0000870_c1 |
|  | Cf_putative | 9841 | Mycolic acid | 100 | BGC0000870_c1 |
|  | Cf_saccharide | 25360 | Glycopeptidolipid | 5 | BGC0000362_c1 |
|  | Other | 43905 | Galbonolides | 10 | BGC0000065_c1 |
|  | Cf_putative | 8037 | Kanamycin | 1 | BGC0000703_c1 |
|  | Cf_putative | 38929 | Rifamycin | 3 | BGC0000136_c1 |
|  | T3pks | 41058 | Alkylresorcinol | 100 | BGC0000282_c1 |
|  | Cf_putative | 12573 | Azinomycin B | 4 | BGC0000960_c1 |
|  | Cf_putative | 21747 | Pimaricin | 11 | BGC0000125_c1 |
|  | Cf_putative | 22689 | Oxazolomycin | 6 | BGC0001106_c1 |
|  | Terpene | 19429 | Isorenieratene | 57 | BGC0000664_c1 |
|  | Cf_putative | 12345 | U-68204 | 14 | BGC0001355_c1 |
|  | T3pks | 41103 | Alkylresorcinol | 66 | BGC0000282_c1 |
|  | Cf_saccharide | 41861 | Sioxanthin | 37 | BGC0001087_c3 |
| *Myco*. P9-22 | Cf_putative | 34214 | Arsenopolyketides | 8 | BGC0001283_c1 |
|  | T1pks-Nrps | 65276 | Mycobactin | 80 | BGC0001021_c1 |
|  | Cf_putative | 13900 | U-68204 | 14 | BGC0001355_c1 |
|  | Cf_putative | 19440 | Rifamycin | 3 | BGC0000136_c1 |
|  | Cf_putative | 21158 | Platensimycin/platencin | 5 | BGC0001140_c1 |
|  | Cf_putative | 15746 | Glycopeptidolipid | 17 | BGC0000362_c1 |
|  | Cf_saccharide | 39868 | Salinomycin | 6 | BGC0000144_c1 |
|  | Cf_saccharide | 23233 | TP-1161 | 8 | BGC0000615_c1 |
|  | Cf_putative | 14727 | Phosphonoglycans | 3 | BGC0000806_c1 |
|  | T3pks | 41088 | Alkylresorcinol | 100 | BGC0000282_c1 |
|  | Other | 43884 | Galbonolides | 10 | BGC0000065_c1 |
|  | Cf_putative | 15894 | Bacillomycin | 20 | BGC0001090_c1 |
|  | Cf_putative | 34272 | Rifamycin | 3 | BGC0000136_c1 |
|  | Bacteriocin | 10797 | Pactamycin | 3 | BGC0000119_c1 |
|  | Terpene | 20944 | Isorenieratene | 71 | BGC0000664_c1 |
|  | T1pks/saccharide/Nrps | 80406 | Glycopeptidolipid | 12 | BGC0000362_c1 |
|  | Nrps | 64660 | Coelichelin | 36 | BGC0000325_c1 |
|  | Nrps | 71027 | Glycopeptidolipid | 13 | BGC0000365_c1 |
|  | Cf_putative | 11813 | Reveromycin | 6 | BGC0000135_c1 |
|  | Cf_putative | 11060 | Glycopeptidolipid | 7 | BGC0000362_c1 |
|  | Terpene | 21196 | Scabichelin | 30 | BGC0000423_c1 |
|  | Cf_putative | 13959 | Yatakemycin | 6 | BGC0000466_c1 |
|  | Cf_putative | 9619 | Galbonolides | 6 | BGC0000065_c1 |
|  | Cf_putative | 14869 | Thiolactomycin | 100 | BGC0001353_c2 |
|  | Cf_putative | 29066 | Phosphonoglycans | 3 | BGC0000806_c1 |
|  | Ectoine | 10392 | Ectoine | 75 | BGC0000853_c1 |
|  | Cf_putative | 22460 | A54145 | 3 | BGC0000291_c1 |
|  | T1pks-Nrps | 59262 | Glycopeptidolipid | 26 | BGC0000365_c1 |
| *Myco.* P9-64 | Cf_putative | 20730 | Reveromycin | 6 | BGC0000135_c1 |
|  | T1pks-Nrps | 54494 | Glycopeptidolipid | 20 | BGC0000365_c1 |
|  | Cf_saccharide | 26286 | Lipopolysaccharide | 8 | BGC0000774_c1 |
|  | Cf_putative | 22459 | Thiotetroamide | 17 | BGC0001236_c1 |
|  | Cf_putative | 10283 | Glycopeptidolipid | 7 | BGC0000362_c1 |
|  | Cf_putative | 22159 | Azinomycin B | 4 | BGC0000960_c1 |
|  | Other | 43923 | Galbonolides | 10 | BGC0000065_c1 |
|  | Cf_saccharide | 23538 | Capreomycin | 6 | BGC0000316_c1 |
|  | Cf_putative | 13748 | Streptomycin | 8 | BGC0000717_c1 |
|  | T1pks-Nrps | 54085 | Glycopeptidolipid | 16 | BGC0000365_c1 |
|  | T3pks | 41070 | Alkylresorcinol | 100 | BGC0000282_c1 |
|  | Nrps | 62835 | Scabichelin | 60 | BGC0000423_c1 |
|  | Cf_putative | 15862 | Kanamycin | 1 | BGC0000703_c1 |
|  | Cf_putative | 15097 | A54145 | 3 | BGC0000291_c1 |
|  | Nrps | 60127 | Mycobactin | 90 | BGC0001021_c1 |
|  | Cf_putative | 11784 | U-68204 | 14 | BGC0001354_c1 |
|  | T1pks | 38603 | Rifamycin | 9 | BGC0000136_c1 |
|  | Cf_putative | 8847 | Maduropeptin | 3 | BGC0001008_c1 |
|  | Cf_putative | 22071 | Meridamycin | 5 | BGC0001011_c1 |
|  | Cf_putative | 11437 | Herbimycin | 6 | BGC0000074_c1 |
|  | T3pks | 41106 | Alkylresorcinol | 66 | BGC0000282_c1 |
|  | Other | 42501 | Asukamycin | 3 | BGC0000187_c1 |
|  | Cf_putative | 26985 | Platensimycin/platencin | 5 | BGC0001140_c1 |
|  | T1pks | 39843 | FK520 | 13 | BGC0000994_c1 |
|  | Cf_putative | 22488 | Bacillomycin | 20 | BGC0001090_c1 |
|  | Cf_putative | 44565 | Mycolic acid | 100 | BGC0000870_c1 |
|  | Cf_saccharide | 40586 | Salinomycin | 6 | BGC0000144_c1 |
|  | Cf_putative | 25444 | Herbimycin | 6 | BGC0000074_c1 |
|  | Cf_putative | 12786 | Mycinamicin | 9 | BGC0000102_c1 |

Suppl. Table 6: Proteins involved in plant growth promotion, resistance and cell wall degradation of strains P1-5, P1-18, P9-22 and P9-64 determined by functional protein annotation based on the eggNOG database.

|  |  | Copy number | | | |  |  |
| --- | --- | --- | --- | --- | --- | --- | --- |
| Category | Protein annotation | Myco. P1-5 | Myco. P1-18 | Myco. P9-22 | Myco. P9-64 | KEGG KOs | Predicted gene name |
| Siderophore | Siderophore-interacting protein | 2 | 3 | 3 | 3 |  | *viu*B, SCLAV_0150 |
| Phosphate solubilization | Pyrroloquinoline quinone biosynthesis (PQQ) protein E | 1 | 1 | 1 | 3 | K06139 | *pqq*E |
|  | Coenzyme PQQ synthesis |  |  | 1 | 1 | K06137, K06138 |  |
| ACC deaminase | 1-aminocyclopropane-1-carboxylate deaminase |  | 1 |  | 1 | K01505, K05396 | *acd*S |
| Antibiotic resistance | Multidrug resistance protein | 2 | 3 | 3 | 6 | K03297, K11741 | *sug*E |
| Antibiotic production | Linocin M18 / bacteriocin protein | 1 | 1 | 1 | 1 |  | *cfp*29 |
|  | Type IV pilus (Peptidase A24A, prepilin) | 1 | 1 | 1 | 1 | K02654 | MT2628 |
|  | Antibiotic biosynthesis monooxygenase | 3 | 6 | 4 | 7 | K09932, K14632, K11530 | Rv2749, *mhu*D, MT1149, Pden_1305 |
|  | Aminoglycoside hydroxyurea antibiotic resistance kinase | 1 | 1 | 1 | 1 | K04343 |  |
| Polyketides | Polyketide synthase | 4 | 3 | 5 | 4 | K15670, K12437, K04791 | *pks*13, *mbt*D |
|  | Polyketide cyclase / dehydrase | 12 | 14 | 15 | 19 |  | Rv2574, Rv1883c |
|  | SnoaL-like polyketide cyclase | 2 | 9 | 8 | 1 |  |  |
| Phenazine biosynthesis | Phenazine biosynthesis PhzC PhzF protein | 1 | 1 | 1 | 2 | K06998 | *phz*F |
| Resistance to oxidative stress | Glutathione S-transferase | 1 | 1 | 1 | 1 | K07393 | *gto*2 |
|  | Rhodanese domain protein | 2 | 3 | 4 | 4 |  | Rv0390, MT1096 |
| Nitrilase | Nitrilase/ cyanide hydratase | 3 | 6 | 6 | 7 | K01501,K12251, K01455, K01431 | *agu*B, *mtn*U |
| Cellulose degradation | Cellulase | 1 |  | 1 | 1 | K05991 |  |
| Lignin degradation | Phenolic acid decarboxylase |  | 1 |  |  | K13727 | *pad*C |
